# Metabolic Soft Spot and Pharmacokinetics: Functionalization of C-3 Position of an Eph–Ephrin Antagonist Featuring a Bile Acid Core as an Effective Strategy to Obtain Oral Bioavailability in Mice

**DOI:** 10.3390/ph15010041

**Published:** 2021-12-28

**Authors:** Francesca Ferlenghi, Carmine Giorgio, Matteo Incerti, Lorenzo Guidetti, Paola Chiodelli, Marco Rusnati, Massimiliano Tognolini, Federica Vacondio, Marco Mor, Alessio Lodola

**Affiliations:** 1 Food and Drug Department, University of Parma, Viale delle Scienze 27/A, 43124 Parma, Italy; francesca.ferlenghi@unipr.it (F.F.); carmine.giorgio@unipr.it (C.G.); matteo.incerti@unipr.it (M.I.); lorenzo.guidetti@unipr.it (L.G.); massimiliano.tognolini@unipr.it (M.T.); alessio.lodola@unipr.it (A.L.); 2 Experimental Oncology and Immunology, Department of Molecular and Translational Medicine, University of Brescia, 25123 Brescia, Italy; paola.chiodelli@unibs.it (P.C.); marco.rusnati@unibs.it (M.R.)

**Keywords:** Eph–ephrin system, UniPR129, UniPR500, metabolite ID, in vivo PK, high-resolution mass spectrometry (HR-MS)

## Abstract

UniPR129, an L-β-homotryptophan conjugate of the secondary bile acid lithocholic acid (LCA), acts as an effective protein-protein interaction (PPI) inhibitor of the Eph–ephrin system but suffers from a poor oral bioavailability in mice. To improve UniPR129 bioavailability, a metabolic soft spot, i.e., the 3α-hydroxyl group on the LCA steroidal ring, was functionalized to 3-hydroxyimine. In vitro metabolism of UniPR129 and 3-hydroxyimine derivative UniPR500 was compared in mouse liver subcellular fractions, and main metabolites were profiled by high resolution (HR-MS) and tandem (MS/MS) mass spectrometry. In mouse liver microsomes (MLM), UniPR129 was converted into several metabolites: M1 derived from the oxidation of the 3-hydroxy group to 3-oxo, M2–M7, mono-hydroxylated metabolites, M8–M10, di-hydroxylated metabolites, and M11, a mono-hydroxylated metabolite of M1. Phase II reactions were only minor routes of in vitro biotransformation. UniPR500 shared several metabolic pathways with parent UniPR129, but it showed higher stability in MLM, with a half-life (*t*_1/2_) of 60.4 min, if compared to a *t*_1/2_ = 16.8 min for UniPR129. When orally administered to mice at the same dose, UniPR500 showed an increased systemic exposure, maintaining an in vitro valuable pharmacological profile as an EphA2 receptor antagonist and an overall improvement in its physico-chemical profile (solubility, lipophilicity), if compared to UniPR129. The present work highlights an effective strategy for the pharmacokinetic optimization of aminoacid conjugates of bile acids as small molecule Eph–ephrin antagonists.

## 1. Introduction

The erythropoietin-producing hepatocellular carcinoma (Eph) receptors are a large family of receptor tyrosine kinases (RTK), classified in the A- and B- subclasses based on sequence homology of the extracellular domain and affinity for their ligands, the ephrins [1]. A unique feature of the Eph–ephrin system is the bidirectional “forward” and “reverse” signaling activated by the interaction of the Eph receptor with its ephrin ligands, which is involved in the modulation of several pathophysiological processes. During the embryonic stage, the Eph–ephrin system is involved in the morphogenesis, while in adults, it helps to maintain the architecture and homeostasis of various epithelial tissues as well as their regeneration [2]. 

Eph receptors and their ligands play a major role in carcinogenesis, and inhibition of the EphA2 receptor subtype has been described as a promising strategy to stop the growth of pancreatic, breast, and lung cancers and to reduce the insurgence of metastases, at least in animal models [3]. Furthermore, it has been shown that EphA2 and EphA3 receptors are highly expressed in glioblastoma multiforme (GBM), where they control angiogenesis and sustain GBM stem cells by renewal of tumor-propagating cells that show stem-like features [4]. 

EphA receptors and ephrin-A ligands are also expressed in the human and rodent pancreas, and Eph–ephrin bidirectional signaling is a transduction mechanism employed by pancreatic β cells to modulate insulin secretion [5]. EphA5 forward signal works as a brake for insulin release whilst ephrin-A reverse signal stimulates it [5]. Thus, the Eph–ephrin system has emerged as a promising target for the development of new chemical entities (NCEs) as anti-angiogenetic and anti-tumor agents as well as novel hypoglycemic agents endowed with a novel mechanism of action [6].

In the last decade, we have designed and synthesized different series of aminoacid conjugates of the secondary bile acids 3α-hydroxy-5β-cholan-24-oic acid, or lithocholic acid (LCA), and of its bioisoster 3β-hydroxy-Δ^5^-cholenic acid, or cholenic acid (CA) [7,8,9,10], which have been characterized in binding and functional in vitro assays as inhibitors of Eph–ephrin protein-protein interaction (PPI). 

**Figure 1:**
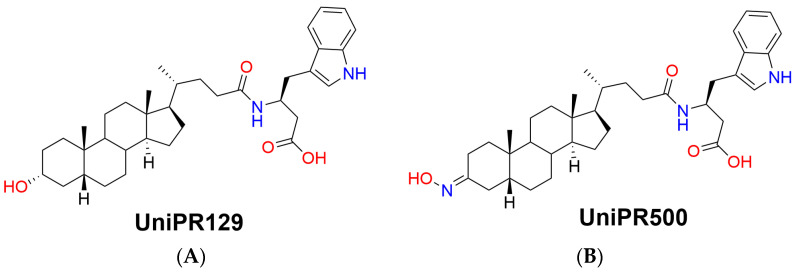
Chemical structures of (**A**) parent Eph–ephrin PPI inhibitor UniPR129 and (**B**) its 3-hydroxyimino derivative UniPR500.

The first potent PPI inhibitor reported by us was UniPR129, the conjugate of LCA with L-β-homotryptophan (Figure 1A), acting as an antagonist at the extracellular domain of the EphA receptors [10]. 

This compound had a promising in vitro pharmacological profile: (i) a submicromolar inhibitory potency on preventing the interaction of ephrin-A1 with the EphA kinase, (ii) anti-angiogenic activity blocking EphA2 phosphorylation in human umbilical vein endothelial cells (HUVEC), and (iii) low cytotoxicity; as a drawback, it showed off-target activity towards bile acid-modulated receptors, such as the G protein-coupled bile acid receptor 1 (GPBAR1, TGR5) and the pregnane X receptor (PXR) and, added to that, when the pharmacokinetic (PK) profile was studied in mice after oral administration of a 30 mg/kg dose, plasma levels of UniPR129 were only detected in the low nanomolar range [7]. Physico-chemical (lipophilicity, solubility) and in vitro ADME (plasma stability, plasma protein binding) properties were first measured to explain the observed behavior. UniPR129 had experimental lipophilicity, expressed as Log *D*_oct,7.4_, equal to 4.90 ± 0.15, and a kinetic solubility in PBS buffer at pH 7.4 of 18.3 ± 2.4 μg/mL [7]. 

It was stable in mouse plasma in vitro since 98.3 ± 9.5% was recovered after 24 h of incubation at 37 °C but tightly bound (%bound = 98%) to plasma proteins. All these in vitro properties could partially justify the in vivo PK data since UniPR129, highly lipophilic, sparingly soluble, highly plasma protein bound, could potentially suffer from an in vivo reduced exposure [7,11].

Within this context, the aim of the present work is to present a pharmacokinetic optimization strategy starting from UniPR129 by chemical modulation of an identified metabolic soft spot, i.e., the 3α-hydroxyl group on the lithocholic acid core ring. UniPR129 structure was modulated by substitution of the 3α-hydroxyl group with a 3-hydroxyimino group, giving rise to the compound UniPR500 (Figure 1B) [11]. 

First, we aimed to investigate the factors determining the low bioavailability of UniPR129 in mice; besides the above-cited unfavorable physico-chemical properties, the low systemic exposure could also depend on UniPR129 liver metabolism, on which we had not gained enough information so far. We thus evaluated herein the in vitro metabolism of UniPR129 in the presence of mouse liver microsomes and S_9_ fraction for phase I and phase II metabolism. A metabolite identification (Met ID) workflow implementing ion mobility (IM)-enabled data acquisition was employed to gain further insight into the structure-metabolism relationships of UniPR129 and of its 3-hydroxyimino derivative UniPR500. Second, we report novel data on UniPR500 interaction with the EphA2 receptor. 

## 2. Results

### 2.1. In Vitro Metabolism of UniPR129

**Figure 2:**
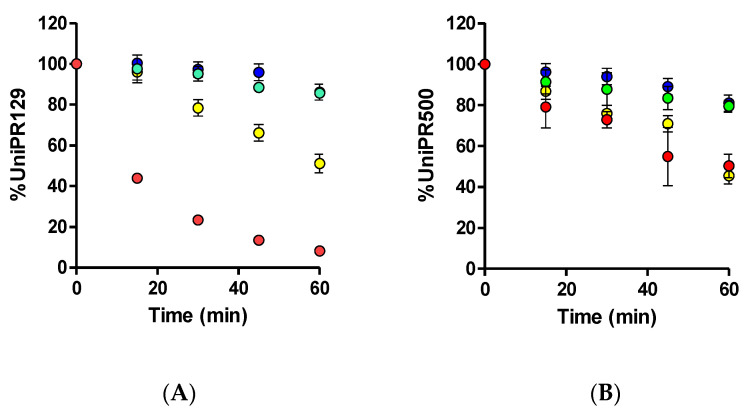
Metabolic stability of (**A**) 1 μM UniPR129 and (**B**) 1 μM UniPR500 to phase I metabolism in MLM (red dots), phase II glucuronidation in MLM (green dots), phase II sulfonation in mouse (blue dots), and human S9 liver fractions (yellow dots).

UniPR129 was first incubated in mouse liver microsomes (MLM) in the presence of an NADPH-generating system to activate CYP450-mediated phase I reactions. UniPR129 was efficiently metabolized by MLM with a corresponding in vitro half-life (*t*_1/2_) of 16.8 ± 1.5 min (Mean ± S.D., *n* = 3, Figure 2A).

This half-life value was in line with those of two known phase I CYP substrates (i.e., verapamil and diclofenac), which showed *t*_1/2_ of ~12 and 6 min [12,13]. No decrease in UniPR129 concentration was observed in the absence of the NADPH-generating system. Stability to phase II glucuronidation and sulfonation was carried out in MLM for the former and in MLS9 and HLS9 fractions for conjugation with active sulphate since sulfonation had already been reported by us as an activated pathway in HLS9 fraction for another Eph–ephrin antagonist [12]. 

UniPR129 was stable to glucuronidation in MLM, with a percentage at *t* = 60 min of incubation equal to 85.7 ± 2.7% (Mean ± S.D., *n* = 3, Figure 2A). 

About sulfonation, UniPR129 showed good stability in MLS9 (Figure 2A), with a percentage at *t* = 60 min equal to 86.2 ± 5.3%, while in HLS9, UniPR129 had an in vitro *t*_1/2_ = 61.1 ± 4.3 min (Mean ± S.D., *n* = 3). This finding is consistent with what is reported in the literature for bile acid metabolism. As compared with humans, sulfonation is a minor phase II pathway in rodents [14,15,16], species that can carry out more extensive phase I hydroxylation of bile acids [16]. 

Since the employed concentration of UniPR compounds in stability assays (i.e., 1 μM) should not lead in principle to CYP450 enzyme saturation [17], we could provide an estimate of phase I metabolism intrinsic clearance values (CL’int) by normalizing the obtained results for incubation volumes and protein content in samples [18]. UniPR129 CL’int was equal to 41.4 μL/min∙mg protein. Classification bands could be used to categorize compounds in terms of in vitro phase I intrinsic clearance and, accordingly, UniPR129 could be classified as a high clearance compound [19].

The in vitro metabolism data needed to be complemented by the in vitro profiling of major UniPR129 metabolites. For Met ID workflow, we employed a full scan MS-experiment for accurate *m/z* data and a parallel ion mobility-enabled data acquisition in which *m/z* data were filtered based on ion mobility drift times to provide higher confidence in structural and fragmentation assignments from high energy MS (MS^E^) spectra.

### 2.2. Profiling of UniPR129 in Vitro Phase I Metabolites 

**Figure 3:**
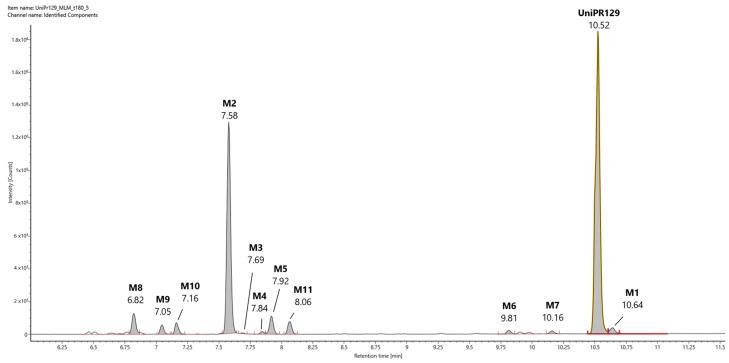
Representative UHPLC-HR-MS chromatogram in positive electrospray ionization (ESI^+^) of a MLM incubation of UniPR129 (100 μM) (*t* = 180 min).

In Figure 3, a representative UHPLC-HR-MS chromatogram in positive (ESI^+^) electrospray ionization of an MLM incubation of UniPR129 (100 μM) at *t* = 180 min is reported. 

UniPR129 was eluted at RT = 10.52 min, with an associated accurate mass of 577.4000 [M + H]^+^ and mass error of 0.1 ppm (Table 1). 

High-energy MS^E^ spectrum of UniPR129 showed a base peak at *m/z* = 559.3893 due to the loss of water, and other peaks at *m/z* = 202.0859, 184.0753, and 156.0804 derived from the cleavage and sequential fragmentation of the L-β-homotryptophan portion of the molecule (Supplementary Material, Figure S1). 

UniPR129 phase I metabolites could be classified as reported in Table 1: M1, deriving from oxidation of the hydroxyl at C-3 to a keto group (−2 Da from parent compound); M2–M7, six metabolites deriving from the addition of one oxygen atom (+16 Da) with a major (M2) and five minor ones (M3–M7); M8–M10, resulting from a double hydroxylation (+32 Da); M11, a second-generation metabolite possibly derived from the oxidation of M1 (+14 Da). 

Their chromatographic and mass spectrometry characterization is detailed below. All the reported *m/z* values refer to experimental masses. 

#### 2.2.1. Metabolite M1

M1 (RT = 10.64 min, Figure 3) had an *m/z* value of 575.3841 [M + H]^+^, 2 Da less than UniPR129, and a mass error of −0.4 ppm. Its high-energy MS^E^ spectrum showed a base peak at *m/z* = 184.0751 and other peaks at *m/z* = 156.0803 and 202.0858 due to the cleavage and sequential fragmentation of the L-β-homotryptophan portion of the molecule as observed for parent compound UniPR129 (Supplementary Material, Figure S2).

#### 2.2.2. Metabolite M2

M2 (RT = 7.58 min, Figure 3) had an *m/z* value of 593.3944 [M + H]^+^, +16 Da with respect to UniPR129, and a mass error of −0.9 ppm. According to full scan analysis of MLM incubation of UniPR129 (Figure 3), at *t* = 180 min M2 was the most abundant of all the mono-hydroxylated metabolites of UniPR129. High-energy MS^E^ spectrum of M2 showed the same cluster of peaks at *m/z* = 202.0857, 184.0752, and 156.0804 due to the sequential fragmentation of the L-β- homotryptophan portion of the molecule observed for UniPR129 and for M1 (Supplementary Material, Figure S3).

#### 2.2.3. Metabolites M3-M7

A careful analysis of the full scan traces in ESI^+^ allowed the identification of at least five other metabolite peaks whose accurate masses differed from that of UniPR129 by +16 Da. The structure of UniPR129 presents multiple positions that could be targeted by a mono-hydroxylation: (i) the steroidal ring, (ii) the alkyl side chain of core lithocholic acid, (iii) the side chain, and (iv) the indole ring of the L-β-homotryptophan.

Indeed, it has been reported that oxidative metabolism on the core structure of C24 bile acids leads to the formation of different hydroxylated metabolites at positions 1, 6, 12, and 16 of the steroidal core structure and position 23 on the side chain [16]. 

Metabolite M3 was not chromatographically resolved from M2 by the 20-min linear gradient, and it eluted very close to it at 7.69 min in the ESI^+^ trace. It corresponded to the elemental composition reported in Table 1 with a mass error of −1.1 ppm. The high-energy MS^E^ spectrum of M3 showed the same cluster of peaks (*m/z* = 202.0856, 184.0754, and 156.0805) of UniPR129, M1, and M2 (Supplementary Material, Figure S4). 

Regarding other peaks (M4−M7) corresponding to the *m/z* value of 593.39, they (i) had lower relative intensities with respect to M2 and (ii) their RT were clustered in two groups (RT = 7.84–7.92 min vs. RT = 9.82–10.16 min) with metabolites M6–M7 showing higher RT on the RP-LC C18 column. Corresponding extracted ion chromatograms, accurate mass values, and high-energy MS^E^ spectra with tentative fragmentation patterns are reported in the Supplementary Material, Figures S5–S8 (M4–M7). 

An ion mobility-enabled metabolite identification workflow was employed to increase the discriminating capacity among the different hydroxylated metabolite isomers. As a principle, the collisional cross-section (CCS) value, the physico-chemical parameter derived from the drift time required by each metabolite ion to go through the ion mobility cell, should consider the different 3D molecular shapes of the metabolites, allowing to gain additional information on metabolite isomers. According to repeated injections of UniPR129 standard, CCS values showed good repeatability, with an RSD < 2%. Based on this tolerance value, only metabolites M3 and M7 did show a significant difference in CCS values with respect to that of UniPR129. Nevertheless, to correlate these differences in CCS to the exact hydroxylation site on the UniPR129 structure, we would still require the synthesis of several mono-hydroxylated derivatives. 

Regarding the MS^E^ fragmentation schemes, metabolites M4–M5 shared the same peaks at *m/z* = 184.0750 (M4) and at *m/z* = 156.0804, 184.0753, 202.0860 already identified for UniPR129 and related to the L-β-homotryptophan portion. Therefore, we might infer that hydroxylation occurred on the other portion of the molecule. 

MS^E^ fragmentation of metabolites M6–M7, instead, was different. MS^E^ spectrum of M6 showed a cluster of peaks at *m/z* = 199.0865 and 217.0916 (Figure S7), which were also shared by M7, also showing peaks at *m/z* = 157.0760 and *m/z* = 172.0755. Both peaks at *m/z* = 199 and 217 differ +15 Da from the previous peaks at *m/z* = 184 and 202, suggesting that hydroxylation might occur on the L-β-homotryptophan portion, possibly involving a hydrogen-bond formation which could increase the overall lipophilicity of the metabolites and, thus, their RT. 

#### 2.2.4. Metabolites M8−M10

Three metabolite peaks M8–M10 differing by +32 Da could also be identified. As mentioned before, the UniPR129 structure presents multiple positions that could be subjected to hydroxylation, making a double hydroxylation also highly probable. M8 (RT = 6.82 min), M9 (RT = 7.05 min), and M10 (RT = 7.16 min) had MS^E^ spectra which shared fragments at *m/z* = 591.4 (loss of H_2_O) and at *m/z* = 573.4 (loss of 2H_2_O). Moreover, MS^E^ spectra of M8 and M10 share a fragment at *m/z* = 235, which could account for double oxidation on the L-β-homotryptophan portion (Supplementary Material, Figures S9–S11). 

#### 2.2.5. Metabolite M11

M11 (RT = 8.06 min, *m/z* = 591.3782 [M + H]^+^, +14 Da than UniPR129) could correspond to a second-generation metabolite of M1. The peak at *m/z* = 573.3682 in the MS^E^ spectrum is indeed due to the loss of water from M1+O [M + H]^+^ ion (Supplementary Material, Figure S12).

### 2.3. Profiling of UniPR129 in Vitro Metabolites: Phase II Metabolism

Liver subcellular fractions (MLM and HLS9 fraction) were then employed for profiling phase II metabolites after conjugation reaction with activated glucuronic acid (UDPGA) and sulphate (PAPS). In MLM incubations, different HPLC conditions and ESI^−^ ionization mode (see Supplementary Material S5) were employed to reveal the presence of two glucuronic acid conjugates of UniPR129, at RT = 23.33 and 25.09 min, which could correspond to the ether glucuronide at the hydroxyl group at C-3 and the acyl glucuronide at the carboxylic acid group of the homotryptophan. GA-conjugates showed a mass shift of +176 Da with respect to UniPR129 with a calculated mass of 751.4175 in ESI^−^ Errors in accurate mass were equal to 0.44 and −0.21 ppm, for M12 and M13, respectively (Table 2 and Figures S13 and S14). 

**Figure 4:**
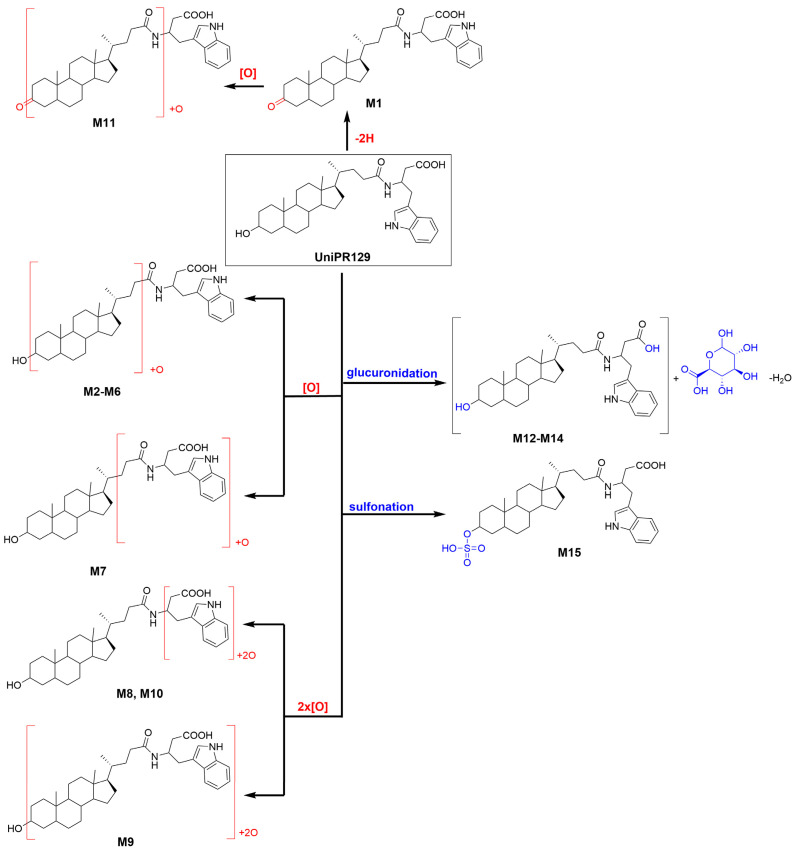
Metabolic tree of UniPR129 plausible phase I and II in vitro biotransformations.

About activated sulphate (PAPS) conjugates, in human liver S9 fraction at RT = 34.88 min, one peak was found having a mass shift of +79 Da and an *m/z* = 655.3411 (mass error = −0.22 ppm) (Table 2 and Figure S15). Sulfonation was supposed to occur at position 3 on the steroidal ring (M14), as supported by literature data on bile acids metabolism [15,16]. The metabolic tree summarizing UniPR129 phase I and II in vitro biotransformations is depicted in Figure 4. 

### 2.4. Design and in Vitro Characterization of UniPR500

**Figure 5:**
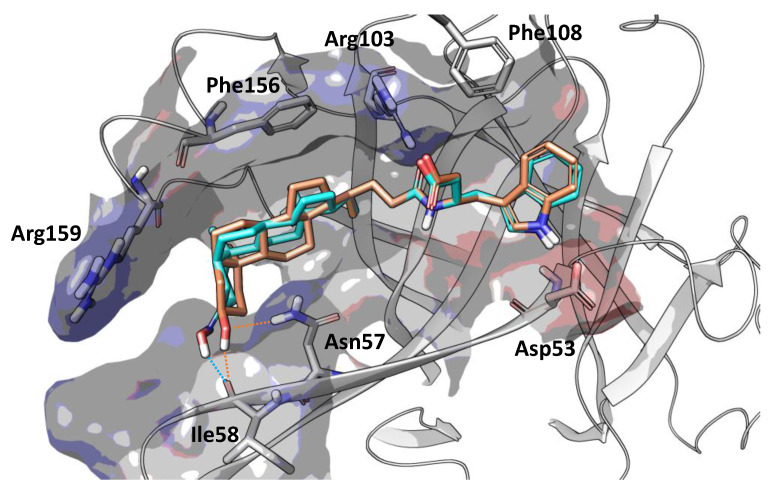
Binding conformation of UniPR129 (orange carbon atoms) and UniPR500 (cyan carbon atoms) within the EphA2 ligand-binding domain (white ribbons). The binding site is represented by Van der Waals surface colored according to the computed electrostatic potential. Key residues of EphA2 binding domain are labeled. Polar interactions between the substituent emerging from position 3 of the steroidal core of the studied antagonists and residues of EphA2 ligand-binding domain are highlighted with dotted orange (UniPR129) or cyan (UniPR500) lines.

Position 3 on the lithocholic acid moiety was identified as one metabolic soft spot of UniPR129. Docking studies using the X-ray structure of EphA2 and previously reported free-energy simulations [9] indicate that polar groups other than 3α-hydroxyl of UniPR129 can form productive interactions with the EphA2 ligand-binding domain (Figure 5). In detail, position 3 of UniPR129 displayed a certain tolerance versus groups able to form hydrogen bonds with the EphA2 receptor as in the case of 3α-(alkylcarbamoyl)oxy substituents, which were reported to prevent ephrinA1 binding to EphA2 in the low micromolar range [20].

UniPR129 structure was therefore modulated by substituting the 3α-hydroxyl group on the lithocholic acid core with a bulkier (and planar) 3-hydroxyimino group, and the resulting compound, UniPR500, was characterized as a new potential EphA2-ephrinA1 antagonist.

This compound was evaluated for its ability to displace ephrin-A1 from EphA2 by using an ELISA-like binding assay. UniPR500 dose-dependently reduced binding of biotinylated ephrin-A1 to EphA2 with an IC50 value of 1.1 μM. Saturation curves describing the binding of ephrin-A1 to EphA2 in the presence of different concentrations of UniPR500 indicate that this compound acts as a competitive antagonist with a Ki for the EphA2 receptor of 0.78 μM. Parent compound UniPR129 had previously revealed a similar in vitro pharmacological profile since it dose-dependently reduced EphA2-ephrin-A1 interactions with an IC_50_ equal to 0.94 μM and the analysis of Schild plots revealed a competitive antagonism behavior with a Ki value for the EphA2 receptor of 0.37 μM [10].

The computational predictions described above, along with the results from the ELISA assay and our previous findings on the effective capacity of lithocholic acid derivatives to bind the EphA2 receptor [7], prompted us to exploit surface plasmon resonance (SPR) to verify and characterize the UniPR500 binding to EphA2 receptor.

**Figure 6:**
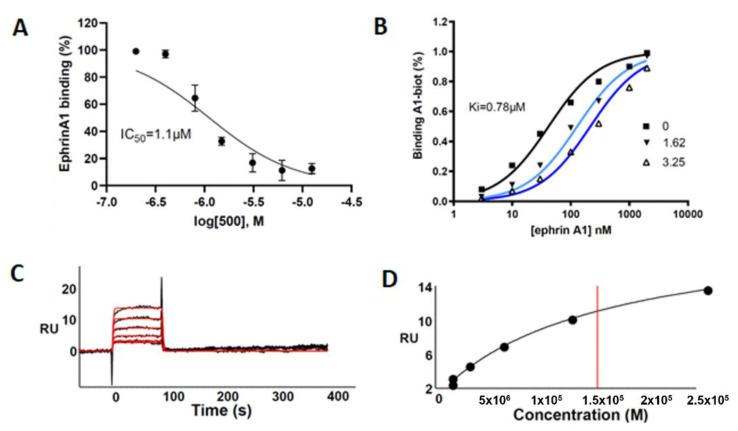
(**A**) UniPR500 dose-dependently displaces biotinylated ephrin-A1 from immobilized EphA2 receptor. (**B**) Saturation curves of biotinylated ephrin-A1-Fc on EphA2 in the presence of increasing concentration of UniPR500. The inhibition constant (Ki) was calculated using Prism non-linear regression analysis. (**C**) SPR sensorgrams for the binding of UniPR500 (25.0, 12.5, 6.25, 3.12, and 1.56 μM from top to bottom) to sensorchip-immobilized EphA2-Fc. Black lines represent the experimental data; red lines represent the fits. (**D**) Steady-state analysis for the interaction of UniPR500 with sensorchip-immobilized EphA2 - Fc. The redline identifies the Kd.

As shown in Figure 6A–D, UniPR500 effectively bound surface-immobilized EphA2 in a concentration-dependent way with saturation binding reached at concentrations around 25 μM. UniPR500/EphA2 binding was very fast and reversible as they readily dissociate, restoring the baseline signal. Accordingly, fast association (k_on_) and dissociation rates (k_off_) were generated by kinetic analysis (1.0 × 10^4^ s^−1^ and 0.59 M^−1^·s^−1^, respectively) that, in turn, provided a dissociation constant (Kd) (measured as koff/kon) equal to 57.2 μM. 

This value is in the same order of magnitude of the corresponding Kd value of 14.9 μM measured from steady-state analysis obtained by plotting the relative binding at equilibrium vs. the ligand concentration and considering the Kd equal to the concentration yielding 50% of the maximum response. Overall, this analysis indicates that the ability of UniPR500 to disrupt the EphA2-ephrin-A1 complex originates from a specific targeting of the EphA2 receptor and that UniPR500 can be regarded as an effective EphA2 binder.

In terms of physicochemical properties, UniPR500 also showed an improved profile, with a measured Log *D*_oct,7.4_ = 4.23 ± 0.11, approximately 0.7 log unit less than UniPR129, and a kinetic solubility of 30.6 ± 2.6 μg/mL. 

To explore how a single, small modulation from a 3-hydroxyl to a 3-hydroxyimino substituent could lead to such marked changes in PK profiles in mice, we next proceeded our investigation focusing on the in vitro metabolism of UniPR500 in mouse subcellular liver fractions to compare its profile with that of UniPR129. 

### 2.5. Profiling of UniPR500 in Vitro Phase I Metabolites in Mouse Liver Microsomes

**Figure 7:**
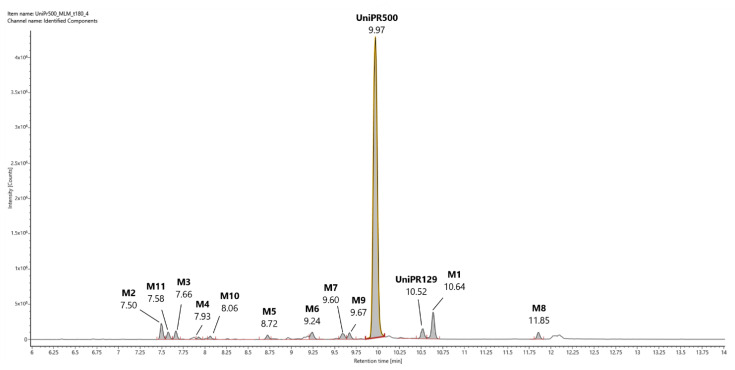
Representative UHPLC-HR-MS chromatogram in positive electrospray ionization (ESI^+^) of a MLM incubation of UniPR500 (100 μM) (*t* = 180 min).

In Figure 7 we provide a representative UHPLC-HR-MS full scan chromatogram in ESI^+^ of an incubation of UniPR500 (100 μM) in MLM (*t* = 180 min). In ESI^+^ trace, UniPR500 was eluted at RT = 9.97 min with an accurate mass of 590.3950 [M + H]^+^ and a mass error of −0.50 ppm. High-energy MS^E^ spectrum had several peaks at: *m/z* = 572.3842 (-H_2_O); 554.3737 (-2H_2_O); 389.3156 (loss of the 4-(indol-3-yl)-butanoyl group, ionized as *m/z* = 202.0857); 372.2899 (break of the amide bond); 354.2786 (*m/z* = 372-H_2_O) and the same cluster of peaks at *m/z* = 202.0857, 184.0752, and 156.0804, already seen in UniPR129, due to fragmentation of the L-β-homotryptophan portion of the molecule (Supplementary Material, Figure S16). 

UniPR500 phase I metabolites are classified in Table 3. M1, deriving from the oxidation of the 3-hydroxyimino group to a 3-keto group (−15 Da from parent compound); UniPR129, deriving from the reduction of M1; M2–M8, a set of metabolites deriving from the addition of one oxygen atom (+16 Da); M9, a double-hydroxylated metabolite, and M10, a second-generation metabolite derived from further oxidation of M1 (+1 Da with respect to UniPR500). Their chromatographic and mass spectrometry features are reported in the following subsections.

#### 2.5.1. Metabolites M1 and UniPR129

M1 (RT = 10.64 min, *m/z* = 575.3838 [M + H]^+^, −15 Da with respect to UniPR500) corresponded to the elemental composition in Table 3 with a mass error of −0.90 ppm. High-energy MS^E^ fragmentation peaks and RT are superimposable with those of metabolite M1 derived from UniPR129. However, in the case of UniPR500, we expect that M1 is generated by the oxidative cleavage of the 3-hydroxyimino group. Interestingly, UniPR129 was also generated from UniPR500 in MLM. The corresponding accurate mass and MS^E^ fragmentation confirmed the identity of UniPR129, reasonably derived from M1 by a reductive pathway. Additionally, CCS values for both M1 and UniPR129 reported in Table 3 were not significantly different (bias < 2%) from those obtained from UniPR129 incubation (Table 1), further proving the identity of the two metabolites (see also Supplementary Material, Figures S17 and S18). 

#### 2.5.2. Mono-Hydroxylated Metabolites M2–M8

Several peaks corresponding to mono-hydroxylated metabolites of UniPR500 could be observed in MLM incubations (Figure 7 and Table 3). Differently from UniPR129, however, all peaks of M+O metabolites showed approximately the same intensities, with no predominance of one over the others. The steroid core of lithocholic acid, as well as the side chain and indole ring of the L-β-homotryptophan portion, were all potential sites of oxidative biotransformation [21]. In the Supplementary Material, Figures S19–S25, we reported the extracted ion chromatograms in ESI^+^, accurate masses, and high-energy (MS^E^) fragmentation spectra for metabolites M2–M8. Regarding the hydroxylation position, for M5 and M7, the shift of the MS^E^ fragmentation peaks observed in UniPR500 (*m/z* = 156.07; 184.07; 202.08) to the corresponding M+16 (*m/z* = 172.07; 200.07; 218.08) supported the hypothesis that hydroxylation occurred on the L-β-homotryptophan portion of the molecule (Figures S22 and S24). In Table 3, we also report the CCS values calculated for each mono-hydroxylated metabolite ion from drift times through the ion mobility cell. However, their uncertainty was within the accepted tolerance in the CCS measurements (RSD ± 2%), thus not sufficient to discriminate among the different isomers.

#### 2.5.3. Di-Hydroxylated Metabolite M9

In line with the higher metabolic stability of UniPR500, we found only one di-hydroxylated metabolite (i.e., M9, RT = 9.68 min, *m/z* = 622.3848 [M + H]^+^, Supplementary Material, Figure S26).

#### 2.5.4. Second-Generation Metabolites Derived from M1 and UniPR129 

We also observed two mono-hydroxylated derivatives of M1 (i.e., M10) and UniPR129 (i.e., M11). M10 (RT = 8.06 min, *m/z* = 591.3785 [M + H]^+^) and M11 (RT = 7.58 min, *m/z* = 593.3942 [M + H]^+^) according to high-energy MS^E^ fragments, shared the hydroxylation pattern on the steroidal core (Supplementary Material, Figure S27 and S28). 

### 2.6. Profiling of UniPR500 in Vitro Phase II Metabolites in MLM and HLS9 Fraction

In Table 4, we report the phase II metabolites of UniPR500 observed in liver subcellular fractions at *t* = 180 min (microsomes and S9 fraction). 

In MLM incubations, the HPLC-ESI-HR-MS traces revealed the presence of three glucuronic acid conjugates, one major (i.e., M12) eluting at RT = 22.77 min and two minor ones (i.e., M13 and M14) eluting at 22.41 and 24.10 min, respectively. In analogy to what had been previously observed for UniPR129, two isomers could correspond to the ether glucuronide at the hydroxyl group of the 3-hydroxyimine and the acyl glucuronide at the carboxylic acid group of the L-β-homotryptophan. Given the presence of a third isomer, we could hypothesize that the attack of the UDPGA to the planar 3-hydroxyimino group could occur on both faces, with one preferred over the other, generating two isomeric GA-conjugates at C-3, which could be partially separated by liquid chromatography. All GA-conjugates had a calculated mass of 764.4128 in ESI^−^ with related mass errors < 0.6 ppm (Table 4). Isotopic distributions and related comparisons with calculated values are reported in Figure S29.

**Figure 8:**
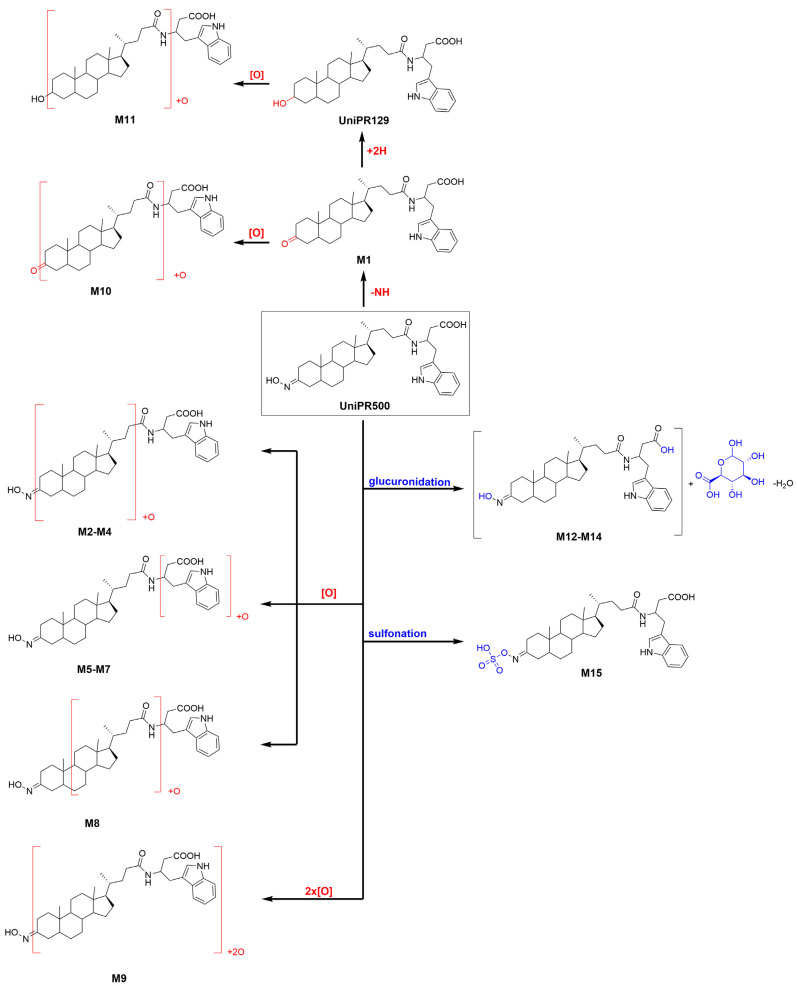
Metabolic tree of Unipr500 plausible phase I and II in vitro biotransformations.

One peak of an active sulphate conjugate (M15) was retrieved in the human liver S9 fraction, with sulfonation occurring at position 3, as already reported for UniPR129 (Supplementary Material, Figure S30). The metabolic tree summarizing UniPR500 phase I and II in vitro biotransformations is depicted in Figure 8.

### 2.7. In Vitro Metabolic stability of UniPR500

UniPR500 was also incubated in the presence of mouse liver subcellular fractions under metabolic stability assay conditions (1 μM, +NADP^+^ for phase I, +UDPGA or +PAPS for phase II, 60 min). As shown in Figure 2B, UniPR500 had an in vitro phase I half-life (*t*_1/2_) of 60.4 ± 12.3 min (Mean ± S.D., *n* = 3), thus being significantly more stable than UniPR129. On the opposite, phase II metabolic stability had a comparable trend with that of UniPR129. UniPR500 was stable to glucuronidation, with a percentage of the intact compound at *t* = 60 min equal to 79.5 ± 2.9% (Mean ± S.D., *n* = 3, Figure 2B). In sulfonation assays, UniPR500 showed good stability in mouse liver S9 fraction, with 81.0 ± 4.0% remaining, and in human liver S9, UniPR500 had an in vitro *t*_1/2_ = 58.6 ± 6.1 min, not significantly different to that of UniPR129.

### 2.8. In Vivo Pharmacokinetics of UniPR129 and UniPR500 in Mice

**Figure 9:**
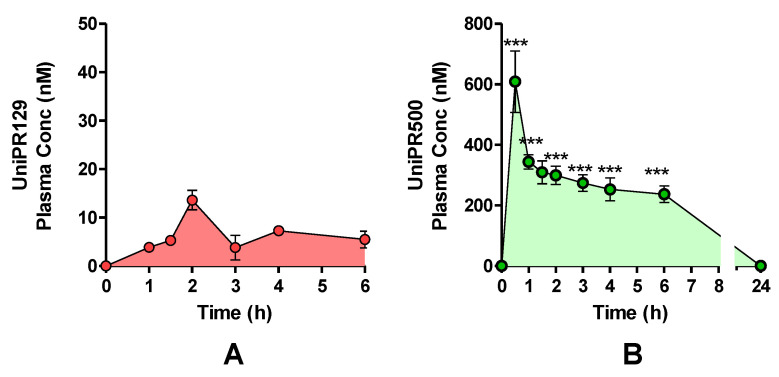
Plasma concentrations after a single UniPR p.o. dose of 30 mg/kg to mice. (mean ± S.E.M.; *n* = 4). (**A**) UniPR129. (**B**) UniPR500. Statistical significance set at ***: *p* < 0.001 by two-tailed Student’s *t* test vs. UniPR129 time points.

One cohort of mice (*n* = 4) was treated with a single oral (p.o.) dose of UniPR129 (30 mg/kg), and plasma levels were monitored by HPLC-MS/MS for a total period of 6 h (Figure 9A). The highest UniPR129 concentration (Cmax) after p.o. administration occurred in plasma at 1 h post-dose (Tmax), and it was equal to 13.6 ± 1.2 nM [7,11]. 

On the contrary, when UniPR500 was orally administered at 30 mg/kg (Figure 9B), at 30 min post-dose (Tmax), it reached a maximal plasma concentration of 609 ± 102 nM (Cmax; mean ± S.E.M., *n* = 4). At 1 h post-dose, UniPR500 concentration was 344 ± 29 nM, while at 6 h post-dose it corresponded to 237 ± 24 nM (mean ± S.E.M., *n* = 4). The area under the curve (AUC) measured for UniPR129 in the 0–6 h period was equal to 40.6 nmol·h/L, while corresponding AUC (0–6 h) for UniPR500 was equal to 1746.9 nmol·h/L, with a 43-fold increase with respect to that of UniPR129.

## 3. Discussion

In the present work, we evaluated the impact of modulating the C-3 position on the lithocholic acid (LCA) steroid core of the Eph–ephrin antagonist UniPR129 on in vitro phase I and II liver metabolism and on in vivo pharmacokinetics in mice. 

Indeed, considering LCA metabolic pathways, the product of oxidation of the 3α-hydroxyl group of LCA, 3-keto-5β-cholanic acid (3-KCA), has been described as one of the three most abundant metabolites in rat liver microsomes [22] and as the major LCA metabolite formed by human recombinant CYP3A4 [23]. 3-KCA has also been proposed as an intermediate in the epimerization of the 3α-hydroxyl group of LCA with the formation of ILCA, a non-P450-mediated reaction, catalyzed by hepatic hydroxysteroid dehydrogenase and steroid oxidoreductase enzymes [24]. 

We found that UniPR129 was converted into several metabolites in MLM: (i) the 3-keto derivative (M1); (ii) six mono-hydroxylated isomers (M2–M7), with M2 (Figure 3, RT = 7.58 min) being the major; (iii) three di-hydroxylated metabolites (M8–M10); and (iv) M11, a second-generation hydroxylated metabolite of a keto one, possibly M1.

Hydroxylation is also described as a major pathway of detoxification of LCA and of other hydrophobic bile acids both in rodents and humans [16,21,25]. LCA is either converted into more hydrophilic bile acids, already belonging to the bile acid pool of the organism or into a bile acid with an atypical hydroxylation pattern, which can then fuel further conjugation reactions and rapid clearance [26]. LCA hydroxylation reactions occur predominantly at position 6 of the steroid, with 6β-hydroxylation to murideoxycholic acid (3α,6β-diOH, MDCA) being a major pathway in rodents [22,27] and 6α-hydroxylation to hyodeoxycholic acid (3α,6α-diOH, HDCA), being a predominant one in humans [25,28]. 

In mouse liver microsomes (MLM), we identified a total of six mono-hydroxylated metabolites of UniPR129, a major one (M2) and five minor ones (M3–M7). We employed an ion mobility-enabled HR-MS workflow to introduce another dimension of separation among the different UniPR129 metabolites and to evaluate its discriminating capacity among the hydroxylated isomers. However, the relative standard deviation (RSD) calculated considering the CCS values of metabolites M2–M7 was 3.4% and, thus, at the same uncertainty level of CCS measurement, which stands at approx. 2–3%. Indeed, the subtle difference in the three-dimensional shape and/or size among hydroxyl isomers could require either a strict calibration with authentic standards for each isomer to improve the precision of determined drift times by ion mobility [29] or chemical derivatization of hydroxyl groups to confer a distinct shape and CCS value on each isomer of hydroxylated metabolites [30]. 

On the other hand, high-resolution, high-energy MS^E^ spectra allowed the generation of diagnostic fragment peaks by high-energy collision-induced dissociations. In particular, the shift towards higher *m/z* values of a cluster of MS^E^ signals corresponding to the sequential fragmentation of the L-β-homotryptophan portion of the molecule in metabolites M6–M7 suggested that hydroxylation could occur in that region of the molecule. 

Within LCA metabolic pathways, di-hydroxylation to α-muricholic acid (3α,6β,7α-triOH, α–CA) or to β–muricholic acid (3α,6β,7β-triOH, β-MCA) has also been reported as a minor pathway both in vitro and in vivo. MCA is produced in both mouse and rat liver, but it is not formed at significant levels in the human liver [26,31]. 

Additionally, in MLM incubations, we have observed the formation of different di-hydroxylated metabolites (i.e., M8–M10, Table 1). 

Moreover, another important pathway of metabolism and detoxification of LCA is conjugation with active sulphate and glucuronic acid. Both conjugation reactions have been described in mammals, even if their relative importance seems to be species dependent. In humans, conjugation of the 3α-hydroxyl group with sulphate seems to be prevalent [16,32], while glucuronide conjugates represent up to 10% of the bile acid circulating pool. Their formation involves the 3α-hydroxyl group or the 24-carboxyl group for the respective formation of the ether 3-glucuronide or the ester 24-glucuronide [33]. Additionally, in the case of UniPR129, phase II conjugation reactions led to the formation of one sulphate metabolite (i.e., 3-sulphate, M12) and two glucuronides, probably an ether at C-3 and an ester on the carboxyl group of the L-β-homotryptophan (M13–M14). In vitro phase II metabolism led, however, to a slower clearance if compared to phase I oxidative biotransformation. 

The shift from the 3α-hydroxyl group of UniPR129 to the more polar 3-hydroxyimino group of UniPR500 had a series of significant consequences on (i) the physico-chemical profile, (ii) in vitro metabolism, and (iii) in vivo mouse pharmacokinetics. The physico-chemical profile of UniPR129 was evaluated with that of UniPR500 in a previous paper [9]. The lipophilicity of UniPR129, expressed as the distribution coefficient in n-octanol/buffer at pH 7.4, diminished from Log *D*_oct,7.4_ = 4.90 to Log *D*_oct,7.4_ = 4.23. This shift is relevant in the attempt to improve the drug-like properties within this class of Eph–ephrin PPI inhibitors. In fact, while for Log *D* values > 5 we may expect to face absorption and bioavailability issues, due to very low solubility, in the 3 < Log *D* < 5 range, a better permeability and absorption might be expected, also sustained by a higher solubility [17]. In drug discovery settings, the following solubility ranges have been proposed to classify new chemical entities: low solubility: < 10 μg/mL; moderate solubility: 10–60 μg/mL; high solubility: > 60 μg/mL [17]. We previously measured kinetic solubility of both compounds, and we reported a value of 18 μg/mL for UniPR129, while for UniPR500, the value almost doubled to reach 30 μg/mL. 

Regarding in vitro metabolism of UniPR500 and comparing it to that of UniPR129, we could not observe significant differences in the number and type of metabolites generated by mouse liver subcellular fractions. 

However, in the case of UniPR500, the 3-keto metabolite M1 was generated together with a small amount of UniPR129. It has been previously reported that hydroxyimine moieties are rather stable to hydrolytic metabolism in plasma and that they are instead efficiently converted to the corresponding keto groups via a CYP450-catalyzed reaction [34,35]. In the case of our MLM incubations, however, metabolite M1 (Figure 7) was not a major in vitro metabolite. The most relevant difference was instead reported in the metabolic stability half-life of UniPR500 in comparison to UniPR129, in a condition at which both compounds were incubated in MLM at non-saturating concentrations. The half-life almost tripled, shifting from 17 min to 60 min. 

Taken together, the slight improvements in physico-chemical profile and the increase in the in vitro metabolic stability led to a very significant increase in in vivo exposure in mice if compared to UniPR129. The introduction of hydroxyimino group at the 3-position also contributed to hampering the recognition by the enzymes of the LCA metabolic pathways, significantly impacting UniPR500 oral bioavailability [15,21].

## 4. Materials and Methods

### 4.1. Chemicals and Reagents

UniPR129 (N-(3α-hydroxy-5β-cholan-24-oyl)-L-β-homotryptophan), UniPR500 (N-(3-hydroxyimino-cholan-24-oyl)-L-β-homotryptophan), UniPR126, and UniPR141, employed as internal standards, were synthesized in our labs as previously described [8,9,11]. Mouse liver (MLM, from CD1 mice, pooled male) microsomes for phase I metabolism and human (pooled male and female) liver S9 fraction (HLS9) and mouse liver S9 fraction (MLS9 from CD1 mice, pooled male) for phase II metabolism were obtained from Xenotech, LLC (Cambridge, Kansas City, KS, USA). Glucose-6-phosphate (G6P), oxidized nicotinamide-adenine-dinucleotide phosphate (NADP^+^), Uridin-di-phosphoglucuronic acid (UDPGA), 3-phosphoadenosin-5-phosphate (PAPS), magnesium chloride (MgCl_2_), dithiothreitol (DTT), and glucose-6-phosphate-dehydrogenase (G6PDH) were supplied by Sigma Aldrich (Milan, Italy). 85% *v/v* formic acid was provided by ACEF Spa (Piacenza, Italy); HPLC-grade acetonitrile (ACN) and dimethyl sulfoxide (DMSO), >99.9% purity, were supplied by Sigma Aldrich (Milan, Italy) and VWR Chemicals (Radnor, PE, USA), respectively. Ultra-pure Millipore water (Darmstadt, Germany) was employed for HPLC mobile phase and sample preparations. 

### 4.2. Docking Simulations

Docking studies were performed with Glid using the atomic coordinates of the EphA2 receptor (pdb code: 3HEI) taken from an equilibrated complex (by plain molecular dynamics simulations) of its ligand-binding domain with UniPR129 [9]. The docking grid was centered in the channel of the EphA2 receptor delimited by Arg103, Phe108, Phe156, and Arg159. Dimensions of enclosing and bounding boxes were set to 20 and 10 Å on each side, respectively. The structure of UniPR500 was built in Maestro and then energy-minimized with MacroModel applying the OPLS3e force field to an energy gradient of 0.01 kcal/(mol·Å). Docking simulations were performed with Glide in Standard Precision mode. The best pose (according to the Gscore) is reported here. 

### 4.3. ELISA Binding Assay

96-well ELISA plates (Costar #2592) were coated with 100 µL/well of 1 µg mL^−1^ EphA2-Fc (R&D Systems, Minneapolis, MN, USA) and incubated overnight at 4 °C. The wells were washed 3 times and blocked for 1 h at 37 °C with a solution containing PBS + 0.5% BSA. The wells were washed again, and UniPR500 was added at proper concentrations in 1% DMSO and incubated at 37 °C for 1 h. Afterward, biotinylated ephrin-A1-Fc (BT602, R&D Systems, USA) was added at its K_D_ value for displacement studies or in a range from 1 to 2000 ng mL^−1^ for saturation studies. The binding of biotinylated ephrin-A1-Fc to EphA2 was detected after 4 h through a standard Streptavidin-HRP-tetramethylbenzidine reaction and read in an ELISA plate reader (Sunrise, TECAN, Switzerland) at 450 nm. IC50 and Ki values were determined using one-site competition non-linear regression analysis with Prism software v. 6.01 (GraphPad Software Inc., San Diego, CA, USA). 

### 4.4. Surface Plasmon Resonance (SPR) Assay

SPR measurements were performed on a BIAcore X100 instrument (GE-Healthcare, Milwaukee, WI, USA), using research-grade CM4 carboxyl-methyl-dextran-coated sensorchips (GE-Healthcare). SPR was exploited to measure changes in refractive index caused by the binding of UniPR500 to surface-immobilized EphA2. To this aim, EphA2-Fc or Fc alone (here used as a negative control and for blank subtraction) were resuspended at 20 μg/mL in 10 mM sodium acetate pH 4.0 and allowed to react with two separate flow cells of a CM4 sensorchip, pre-activated with 50 mL 0.2 M N-ethyl-N-(3-diethylaminopropyl) carbodiimide hydrochloride and 0.05 M N-hydroxysuccinimide, leading to the immobilization of 3000 and 900 RU for EphA2-Fc and Fc fragment, respectively (equal to approximately 40 fmol/mm^2^ for both the proteins). Increasing concentrations of UniPR500 in PBS, 0.05% surfactant P20 and 5% DMSO, pH 7.4 were injected over the EphA2 or Fc surfaces for 90 s and then washed until dissociation was observed. Binding parameters were calculated by the non-linear curve fitting software package BIA evaluation 3.2 using a single site model with correction for mass transfer. The dissociation constant (Kd) was either derived from the dissociation rate (koff)/association rate (kon) ratio (kinetics) or by steady-state analysis (being fitted with the proper form of Scatchard’s equation for the plot of the bound RU at equilibrium vs. the ligand concentration in solution).

### 4.5. In Vitro Phase I and II Liver Metabolism: Clearance and Metabolite ID 

Phase I metabolic stability assays on UniPR compounds were carried out in the presence of MLM (final protein concentration: 1 mg/mL) following published procedures [12]. MLM were also employed in phase II glucuronidation assays employing UDPGA as activating co-factor. 

Phase II sulfonation assays were carried out in the presence of HLS9 or MLS9 fractions, as previously reported [12]. The co-factor PAPS was incubated together with MgCl2, DTT, and UniPR compounds. The detailed experimental protocols for in vitro phase I and II metabolic stability assays are reported in Sections S.1–S.3 of the Supplementary Material. For phase I and II metabolite identification (Met ID workflow), UniPR129 and UniPR500 were incubated in samples at the concentration of 100 µM. A 100 µL aliquot of the reaction mixture was extracted at the beginning of the experiment (*t* = 0) and at *t* = 180 min and processed as for the metabolic stability samples.

### 4.6. In Vivo Dosing of UniPR129 and UniPR500 in Mouse Plasma

Male mice (breed: C57BL/6J) were supplied by Charles River Labs, Milan, Italy [7]. Mice were submitted to 12 h light-dark cycles under standard temperature and humidity conditions, with free access to water and chow. All experimental procedures were carried out following the European Community Council Directive 2010/63/UE, Italian regulations (DL 26/2014), and the ethical committee guidelines for animal research. Two different experimental groups of male mice were administered: (i) 30 mg/kg UniPR129 p.o. as a suspension in 0.5% carboxymethylcellulose (CMC) and (ii) 30 mg/kg UniPR500 p.o. as a suspension in 0.5% CMC. Blood aliquots were withdrawn by tail puncture into heparinized tubes, centrifuged to collect plasma (2000× *g*, 10 min, 4 °C), and stored at −80 °C until analysis by HPLC-ESI-MS/MS. Details on the employed bioanalytical method and HPLC-ESI-MS/MS analytical conditions are given in Section S.4 of the Supplementary Material. 

### 4.7. IM-Enabled Data Acquisition for Phase I Metabolite ID

A Waters Acquity UHPLC I-Class (Waters Corp, Milford, MA, USA) coupled to a Waters Vion IMS QToF was employed for ion mobility-enabled acquisitions in Met ID workflow. The Vion IMS QToF was calibrated daily employing Waters Major Mix (Waters, Milford, MA, USA), and system performance was checked daily for accurate mass and Collisional Cross Section (CCS) accuracy and precision by injecting system suitability test (SST) mixture composed of nine QC standards. For UHPLC separation, mobile phases A and B were ACN and ultra-pure water, both added with 0.1% *v*/*v* formic acid and column was an Acquity UHPLC CSH C18 (2.1 × 100 mm, 1.7 µm; Waters, Milford, MA, USA). The linear gradient was: 0 min: 5% A; 0–15 min: 5–95% A; 15–16 min: 95% A; 16–17 min: 95–5% A; 17–20 min: 5% A. Total run time: 20 min. Flow rate: 0.40 mL/min; injection volume: 5 µL; column temperature: 50 °C. Acquisition range: *m/z* = 100–800 amu. Instrumental parameters were set as follows: source temperature: 120 °C; desolvation temperature: 500 °C; source gas flow: 20 L/h; desolvation gas flow: 800 L/h; capillary voltage: 0.8 kV (ESI^+^) and 1.0 kV (ESI^−^); cone voltage: 20 V (ESI^+^ and ESI^−^); collision energy: low energy: 4 eV; high energy: 10–45 eV; reference mass: leucine enkephalin [M + H]^+^ *m/z* = 556.2766. The software UNIFI v.1.8.2 (Waters, Milford, MA, USA) was employed for data acquisition and processing. The Met ID workflow implemented in the software UNIFI was employed (i) to predict potential phase I metabolites starting from the molecular structure of UniPR129 and UniPR500 and (ii) to search for matches in the low energy full scan mass spectra. Manual check was required to discard false attributions, which were also detected in control incubations without UniPR compounds.

### 4.8. HPLC-ESI-HR-MS Analytical Conditions for Phase II Metabolite ID

A configuration composed of a Dionex HPLC system (Thermo, Waltham, MA, USA) coupled with a Thermo LTQ-Orbitrap HR mass analyzer with a heated electrospray (H-ESI) ion source (Thermo, USA) was employed for phase II metabolite identification. Instrumental settings and details on the analytical method are reported in Section S.5 of the Supplementary Material. 

### 4.9. Data Analysis 

In vitro half-life (*t*_1/2_) in MLM and MLS9 fraction was calculated from the slope (k, Equation (1)) of the linear regression model obtained by fitting the log percentage UniPR compound remaining vs. time [18].

in vitro *t*_1/2_ = −0.693/k (1)

In vitro intrinsic clearance (CL’int) was expressed as in Equation (2):

CL’int (µL/min·mg protein) = (Volume of incubation·ln2)/(mg protein in the incubation·*t*_1/2_) (2)

Microsoft Excel 365 (Microsoft Corp., Redmond, WA, USA) was employed for data analysis. GraphPad Prism v. 6.01 (GraphPad Software Inc., La Jolla, CA, USA) was employed for plotting graphs. The freeware software PCModFit V.6.7 (Add-on for Microsoft Excel) was employed for AUC calculations by non-compartmental analysis (NCA).

## 5. Conclusions

The chemical modulation at the C-3 of the LCA steroid portion of the Eph–ephrin PPI inhibitor UniPR129 has allowed us to explore its in vitro structure–metabolism relationships in phase I and II metabolic reactions. The substitution of the 3α-hydroxyl with the 3-hydroxyimino group in UniPR500 has proven to be a successful strategy to improve the overall physico-chemical profile as well as to ameliorate the oral bioavailability in mice, without a loss in activity and selectivity. The knowledge acquired from these data will be further utilized in the design and synthesis of novel analogs endowed with optimized pharmacokinetic profiles.

## Figures and Tables

**Table 1:** Phase I putative metabolites of UniPR129 in mouse liver microsomes.

Compound	Ion	RT (min)	Calculated Mass	Experimental Mass	Δm (ppm)	Observed CCS (Å²)	Chemical Formula	Mass Shift
UniPR129	[M + H]^+^	10.52	577.4000	577.4000	0.1	228.89	C36H52N2O4	--
M1	[M + H]^+^	10.64	575.3843	575.3841	−0.4	228.93	C36H50N2O4	−2H
M2	[M + H]^+^	7.58	593.3949	593.3944	−0.9	231.09	C36H52N2O5	+O
M3	[M + H]^+^	7.69	593.3949	593.3942	−1.1	250.25	C36H52N2O5	+O
M4	[M + H]^+^	7.84	593.3949	593.3942	−1.1	230.52	C36H52N2O5	+O
M5	[M + H]^+^	7.92	593.3949	593.3946	−0.4	231.13	C36H52N2O5	+O
M6	[M + H]^+^	9.81	593.3949	593.3934	−2.6	234.45	C36H52N2O5	+O
M7	[M + H]^+^	10.16	593.3949	593.3934	−2.5	243.15	C36H52N2O5	+O
M8	[M + H]^+^	6.82	609.3898	609.3893	−0.8	237.80	C36H52N2O6	+2O
M9	[M + H]^+^	7.05	609.3898	609.3895	−0.5	233.19	C36H52N2O6	+2O
M10	[M + H]^+^	7.16	609.3898	609.3893	−0.7	248.46	C36H52N2O6	+2O

**Table 2:** Phase II putative metabolites of UniPR129 in liver microsomes and S9 fraction.

Compound	Ion	RT (min)	Calculated Mass	Experimental Mass	Δm (ppm)	Chemical Formula	Mass Shift
M12	[M − H]^−^	25.09	751.4175	751.4178	0.44	C_42_H_60_N_2_O_10_	+176
M13	[M − H]^−^	23.33	751.4175	751.4174	−0.21	C_42_H_60_N_2_O_10_	+176
M14	[M − H]^−^	34.88	655.3421	655.3411	−0.22	C_36_H_52_N_2_O_7_S	+79

**Table 3:** Phase I putative metabolites of UniPR500 in mouse liver microsomes.

Compound	Ion	RT (min)	Calculated Mass	Experimental Mass	Δm (ppm)	Observed CCS (Å²)	Chemical Formula	Mass Shift
UniPR500	[M + H]^+^	9.97	590.3952	590.3950	−0.5	235.54	C36H51N3O4	--
M1	[M + H]^+^	10.64	575.3843	575.3838	−0.9	230.52	C36H50N2O4	−NH
UniPR129	[M + H]^+^	10.52	577.4000	577.3988	−2.1	228.92	C36H52N2O4	−N + H
M2	[M + H]^+^	7.50	606.3901	606.3890	−1.9	239.09	C36H51N3O5	+O
M3	[M + H]^+^	7.66	606.3901	606.3892	−1.6	237.17	C36H51N3O5	+O
M4	[M + H]^+^	7.93	606.3901	606.3886	−2.5	235.81	C36H51N3O5	+O
M5	[M + H]^+^	8.72	606.3901	606.3889	−2.1	239.62	C36H51N3O5	+O
M6	[M + H]^+^	9.24	606.3901	606.3886	−2.5	235.55	C36H51N3O5	+O
M7	[M + H]^+^	9.59	606.3901	606.3891	−1.7	239.24	C36H51N3O5	+O
M8	[M + H]^+^	11.85	606.3901	606.3894	−1.2	235.89	C36H51N3O5	+O
M9	[M + H]^+^	9.68	622.3851	622.3848	−0.4	237.29	C36H51N3O6	+2O
M10	[M + H]^+^	8.06	591.3792	591.3785	−1.4	233.68	C36H50N2O5	−NH + O
M11	[M + H]^+^	7.58	593.3949	593.3942	−1.2	232.71	C_36_H_52_N_2_O_5_	− N + OH

**Table 4:** Phase II putative metabolites of UniPR500 in liver microsomes and S9 fraction.

Compound	Ion	RT (min)	Calculated Mass	Experimental Mass	Δm (ppm)	Chemical Formula	Mass Shift
M12	[M − H]	22.77	764.4128	764.4132	0.58	C_42_H_59_N_3_O_10_	+176
M13	[M − H]	22.41	764.4128	764.4128	0.01	C_42_H_59_N_3_O_10_	+176
M14	[M − H]	24.10	764.4128	764.4130	0.33	C_42_H_59_N_3_O_10_	+176
M15	[M − H]	32.88	668.3375	668.3372	0.21	C_36_H_51_N_3_O_7_S	+78

## Data Availability

Data is contained in the article and Supplementary Materials.

## Supplementary Materials

The following are available online at https://www.mdpi.com/article/10.3390/ph15010041/s1. Experimental procedures for in vitro metabolic stability assays. LC-HRMS and MS^E^ mass spectra of UniPR129 and UniPR500 metabolites.
