# Supplementary material for: Metabolic Soft Spot and Pharmacokinetics: Functionalization of C-3 Position of an Eph–Ephrin Antagonist Featuring a Bile Acid Core as an Effective Strategy to Obtain Oral Bioavailability in Mice
Source: Pharmaceuticals (Basel). 2021 Dec 28;15(1):41. doi: 10.3390/ph15010041 (PMC8779995; doi:10.3390/ph15010041)

Metabolic soft spot and pharmacokinetics: functionalization of C-3 position of an Eph-ephrin antagonist featuring a bile acid core as an effective strategy to obtain oral bioavailability in mice

Francesca Ferlenghi[1], Carmine Giorgio[1], Matteo Incerti[1], Lorenzo Guidetti[1], Paola Chiodelli[2], Marco Rusnati[2], Massimiliano Tognolini[1], Federica Vacondio[1,*] Marco Mor[1,*] and Alessio Lodola[1]

[1] Food and Drug Department, University of Parma, Viale delle Scienze 27/A, 43124 Parma, Italy.

[2] Experimental Oncology and Immunology, Department of Molecular and Translational Medicine, University of Brescia, 25123 Brescia, Italy.

Supplementary Material

Sections and Figures

S.1 *In vitro* phase I liver metabolism in MLM 4

S.2 *In vitro* phase II liver metabolism: glucuronidation in MLM........ 4

S.3 *In vitro* phase II liver metabolism: sulfonation in HLS_9 and MLS_9 fractions........ 4

S.4 *In vivo* dosing of UniPR129 and UniPR500 in mouse plasma: HPLC-ESI-MS/MS method....... 4

S.5 *In vitro* phase II metabolite profiling: HPLC-HR-MS method 5

S.6 UniPR129 *In vitro* phase I metabolite profiling........ 6

Figure S1. a-c. Parent compound UniPR129 in MLM 6

Figure S2. a-d. Metabolite **M1** in MLM. 8

Figure S3. a-d. Metabolite **M2** in MLM. 10

Figure S4. a-d. Metabolite **M3** in MLM. 13

Figure S5. a-d. Metabolite **M4** in MLM. 15

Figure S6. a-d. Metabolite **M5** in MLM. 17

Figure S7. a-d. Metabolite **M6** in MLM. 19

Figure S8. a-d. Metabolite **M7** in MLM. 22

Figure S9. a-c. Metabolite **M8** in MLM. 24

Figure S10. a-c. Metabolite **M9** in MLM. 26

Figure S11. a-c. Metabolite **M10** in MLM. 28

Figure S12. a-d. Metabolite **M11** in MLM. 30

S.7 UniPR129 *In vitro* phase II metabolite profiling........ 33

Figure S13. a-b. Metabolite **M12** in MLS_9 fraction. 33

Figure S14. a-b. Metabolite **M13** in MLS_9 fraction. 34

Figure S15. a-b. Metabolite **M14** in HLS_9 fraction. 35

S.8 UniPR500 *In vitro* phase I metabolite profiling........ 36

Figure S16. a-c. Parent compound UniPR500 in MLM. 36

Figure S17. a-d. Metabolite **M1** in MLM. 38

Figure S18. a-c. UniPR129 derived from UniPR500 in MLM. 40

Figure S19. a-d. Metabolite **M2** in MLM. 42

Figure S20. a-d. Metabolite **M3** in MLM. 44

Figure S21. a-d. Metabolite **M4** in MLM. 48

Figure S22. a-d. Metabolite **M5** in MLM. 50

Figure S23. a-d. Metabolite **M6** in MLM. 52

Figure S24. a-d. Metabolite **M7** in MLM. 54

Figure S25. a-d. Metabolite **M8** in MLM. 56

Figure S26. a-c. Metabolite **M9** in MLM. 58
Figure S27. a-d. Metabolite **M10** in MLM. 60
Figure S28. a-d. Metabolite **M11** in MLM. 62
S.9 UniPR500 *In vitro* phase II metabolite profiling 65
Figure S29. a-d. Metabolites **M12-M14** in MLS_9 fraction. 65
Figure S30. a-b. Metabolite **M15** in HLS_9 fraction. 67

S.1 *In vitro* phase I liver metabolism in MLM

In phase I metabolic stability assays, samples were prepared in 100 mM phosphate buffered saline (PBS), pH 7.4, containing a NADPH-generating system (5 mM $MgCl_2$, 10 mM glucose-6-phosphate, 2 mM $NADP^+$, 1.5 U/mL of glucose-6-phosphate dehydrogenase) and 1 mg/mL mouse liver microsomes. After incubation under stirring (5 min, 37°C), 100 µM UniPR129 and UniPR500 stock solutions in DMSO were added to start the reaction. Final DMSO percentage in samples was kept at 1%. At fixed time points (t = 0, 15, 30, 45, 60 min), single aliquots of reaction mixture were withdrawn, processed with two vol. of ACN containing 100 nM UniPR126, centrifuged (16000 g, 10 min, 4 °C) and the supernatant was directly injected in the HPLC-ESI-MS/MS system.

Results were reported as percentage (%) of compound remaining *vs.* time. We verified the absence of unspecific binding to MLM proteins by means of control samples in which UniPR compounds were incubated in the absence of a NADPH generating system.

S.2 *In vitro* phase II liver metabolism: glucuronidation in MLM

8 mM Uridin-di-phosphoglucuronic acid (UDPGA), 100 µM saccharic-acid-1,4-lactone, 10 mM magnesium chloride (MgCl2), 1 mM ethylenediamino-tetracetic acid (EDTA) and 500 µM 3-[(3-cholamidopropyl)dimethylammonium]-1-propanesulfonate (CHAPS) solutions were prepared independently in 100 mM TRIS buffer, pH = 7.7. Assay samples contained: (i) 15 µl of MLM (final protein concentration = 1 mg/ml); (ii) 30 µl of each co-factor solution; (iii) 132 µL of TRIS buffer. After pre-incubation for 5 min at 37 °C, UniPR129 and UniPR500 stock solutions in DMSO was added to each sample. Aliquot sampling, quenching of reaction and centrifugation steps occurred as detailed in section S.1.

S.3 *In vitro* phase II liver metabolism: sulfonation in HLS_9 and MLS_9 fractions

5 mM 3-phosphoadenosin-5-phosphate (PAPS), 2.5 mM $MgCl_2$ and 1 mM dithiothreitol (DTT) solutions were prepared separately in 100 mM PBS, pH = 7.4. Assay samples contained: (i) 15 µl of HLS_9 or MLS_9 to reach a final protein concentration of 1 mg/ml (ii) 50 µl of $MgCl_2$ and (iii) 50 µl of DTT solution in PBS. UniPR compounds stock solution in DMSO was spiked into the samples (final compound concentration: 1 µM). 15 µl of PAPS solution were also added and each sample was incubated at 37 °C. Aliquot sampling and sample processing steps occurred as detailed in section S.1.

S.4 *In vivo* dosing of UniPR129 and UniPR500 in mouse plasma: HPLC-ESI-MS/MS method

Calibration standards (CS, concentration range: 1000-2 nmol/L) were prepared by spiking blank mouse plasma with stock solutions of UniPR129 and UniPR500 in DMSO (final DMSO concentration = 1%). Specificity of the method was checked comparing HPLC-MS/MS chromatograms of blank plasma samples to those of UniPR129 and UniPR500 at the Lowest Limit of quantification (LLOQ). Within-run accuracy and precision were checked employing QC samples representative of the whole calibration range (HQC = 800 nmol/l; MQC = 400 nmol/l; LQC = 5 nmol/l) with n = 3 replicates at each QC concentration level. Between-run accuracy and precision were checked employing QC samples at n=3 concentration levels in n = 3 analytical runs over three days. The acceptance criteria for accuracy were to get back-calculated concentrations ± 15% of the nominal ones, except for the LLOQ, which had to be ± 20%. The acceptance criteria for precision, expressed as relative standard

deviation (%RSD), was not to exceed 15%, except for the LLOQ, where it had to be not more than 20%. All plasma samples (CS, QC and unknown) from the fast PK experiments were all processed by addition of a double volume of ACN containing 100 nM UniPR126 as internal standard, centrifuged (16000 g, 10 min, 4 °C) and the supernatant was analyzed by HPLC-ESI-MS/MS. Recovery and Matrix effect were measured on LQC and HQC samples (n=3). Matrix effect was determined by comparison of the analyte/internal standard ratios in QC samples prepared as pure standards in buffered solution, processed by two vol of ACN with the analyte/IS ratios in QC samples prepared in biological matrix (mouse plasma), processed by ACN addition before the spiking with the analyte. Recovery was determined by comparison of the analyte/IS ratios in QC samples prepared by spiking the analyte in mouse plasma samples before ACN addition with the analyte/IS ratios in QC samples prepared in biological matrix (mouse plasma), pre-processed by ACN addition.

Chromatographic traces were acquired on a Thermo Accela UHPLC system (Thermo, USA), employing a Synergy Fusion C_{18} 80 Å RP-column (2.0 x 100 mm, 4 µm: Phenomenex, USA) in gradient elution. Gradient was as follows: eluent A: ACN; eluent B: MilliQ water, both added with 0.1% v/v HCOOH. t = 0 min: 5%A; t = 0-3 min: 5-95%A; t = 3-6 min: 95%A; t = 6-7 min: 95-5%A; t = 7-10 min: 5%A. Total run time: 10 min. Flow rate: 0.35 ml/min; injection volume: 10 µl. The UHPLC system was coupled to a Thermo TSQ Quantum Access Max Triple Quadrupole mass spectrometer (Thermo, USA), with a heated electrospray (H-ESI) ion source. Ion source voltage was set at 3500 V; Capillary temperature at 270 °C; sheath gas (N_2) at a pressure of 35 psi and auxiliary gas (N_2) at a pressure of 15 psi; Argon was employed as collision gas at a pressure of 1.5 mtorr. Mass spectrometer operated in positive ion (ESI^+) and in Multiple Reaction Monitoring (MRM) mode following the parent-product transitions of UniPR compounds. Xcalibur software v. 2.2 (Thermo, USA) was employed for both acquisition and processing of HPLC-MS/MS traces. Tube lens voltages (TL) and collision energies (CE) for each transition were optimized by Flow Injection Analysis (FIA). UniPR129: *m/z* = 577.4 $[M+H]^+$→ *m/z* = 358.2, 202.1, 184.1 (TL = 111.9 V; CE = 26, 29, 31 eV, respectively); UniPR500: *m/z* = 590.4 $[M+H]^+$→ *m/z* = 572.6, 389.4, 184.1 (TL = 99.4 V; CE = 21, 22, 36 eV); UniPR126 (IS): *m/z* = 563.4 $[M+H]^+$→ *m/z* = 545.8, 187.9, 146.1 (TL = 81.8 V; CE = 20, 25, 48 eV); UniPR141 (IS): *m/z* = 586.4 $[M-H]^-$→ *m/z* = 374.3, 228.1, 167.1 (TL = 134 V; CE = 42, 37, 40 eV, respectively); verapamil: *m/z* = 455.3 $[M+H]^+$→ *m/z* = 303.2, 165.1, 150.1 (TL = 85 V; CE = 26, 27, 34 eV); diclofenac sodium salt: *m/z* = 294.0 $[M-H]^-$→ *m/z* = 250.0, 214.1, 178.1 (TL = 34 V; CE = 15, 24, 34 eV).

S.5 *In vitro* phase II metabolite profiling: HPLC-HR-MS method

The Thermo LTQ-Orbitrap mass spectrometer was weekly calibrated in ESI^+ mode by Pierce ESI Positive Ion Calibration solution (Thermo, USA) and in ESI^- mode by Pierce ESI Negative Ion Calibration solution (Thermo, USA) following manufacturer instructions. Tolerance for accurate mass measurements was set at 3 ppm in the mass range *m/z* = 50-2000 amu. A linear gradient elution on a Phenomenex Synergy Fusion C_{18} 80 Å RP-column (2.0 x 100 mm, 4 µm; Phenomenex, USA) was chosen for phase II metabolite profiling. Eluent A was ACN; eluent B was Milli-Q water. Both phases were acidified by 0.2% HCOOH. Gradient was as follows: t = 0-3 min: 5%A; t = 3-33 min: 5-95%A; t = 33-38 min: 95%A; t = 38-39 min: 95-5%A; t = 39-45 min: 5%A. Total run time: 45 min. Flow rate: 0.20 ml/min; injection volume: 5 µL. HR-MS scans were performed in Full Scan mode (*m/z* = 100-1600 amu). Capillary temperature was set at 275 °C; sheath gas pressure was 20 psi; auxiliary gas pressure

was 5 psi; sweep gas pressure was 5 psi; ESI source voltages were set at 3000 V in ESI$^+$ and 2100 V in ESI$^-$; capillary voltage was set at 13 V in ESI$^+$ and 35 V in ESI$^-$; tube lens voltage was set at 85 V in ESI$^+$ and -110 V in ESI$^-$. In Full Scan mode, resolution was set at 100 000.

S.6 UniPR129 *In vitro* phase I metabolite profiling

Figure S1. *a-c. Parent compound UniPR129 in MLM*

a. Extracted ion chromatogram (XIC) in ESI$^+$ at *m/z* = 577.40 [M+H]$^+$ and RT = 10.52 min.

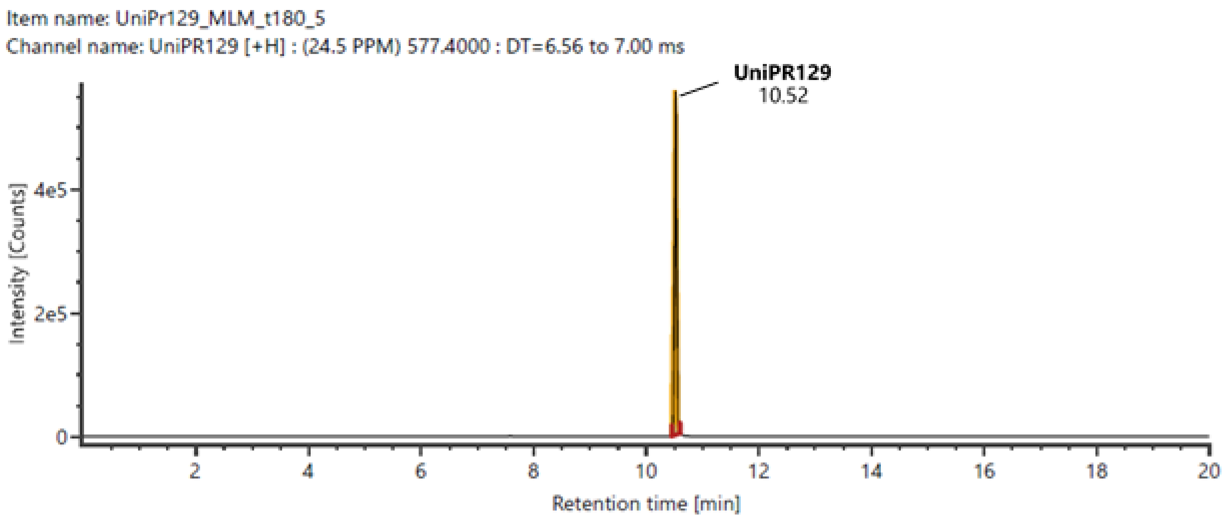

b. Experimental high resolution mass value in ESI$^+$ for parent compound UniPR129.

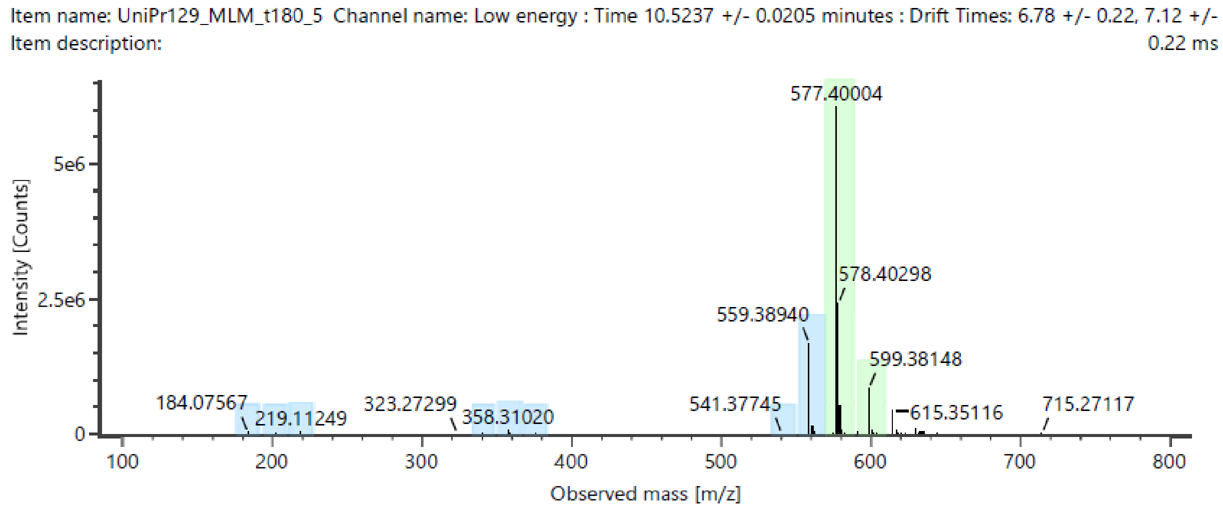

c. High resolution high energy (MSE) MS spectrum in ESI+ of UniPR129 together with tentative fragmentation pattern. The base peak at *m/z* = 559.4 corresponds to the loss of water (-18). Fragment ion at *m/z* = 202.1 is compatible with the 4-(indol-3-yl)butanoic acid fragment, while product ions at *m/z* = 184.1 and 156.1 could account for consecutive loss of water (-18 with respect to 202.1) and carbonyl (-28 with respect to 156.1) group. One or more of these three ion products are in the MS/MS spectrum of UniPR129 metabolites M1-5, M9 and M11.

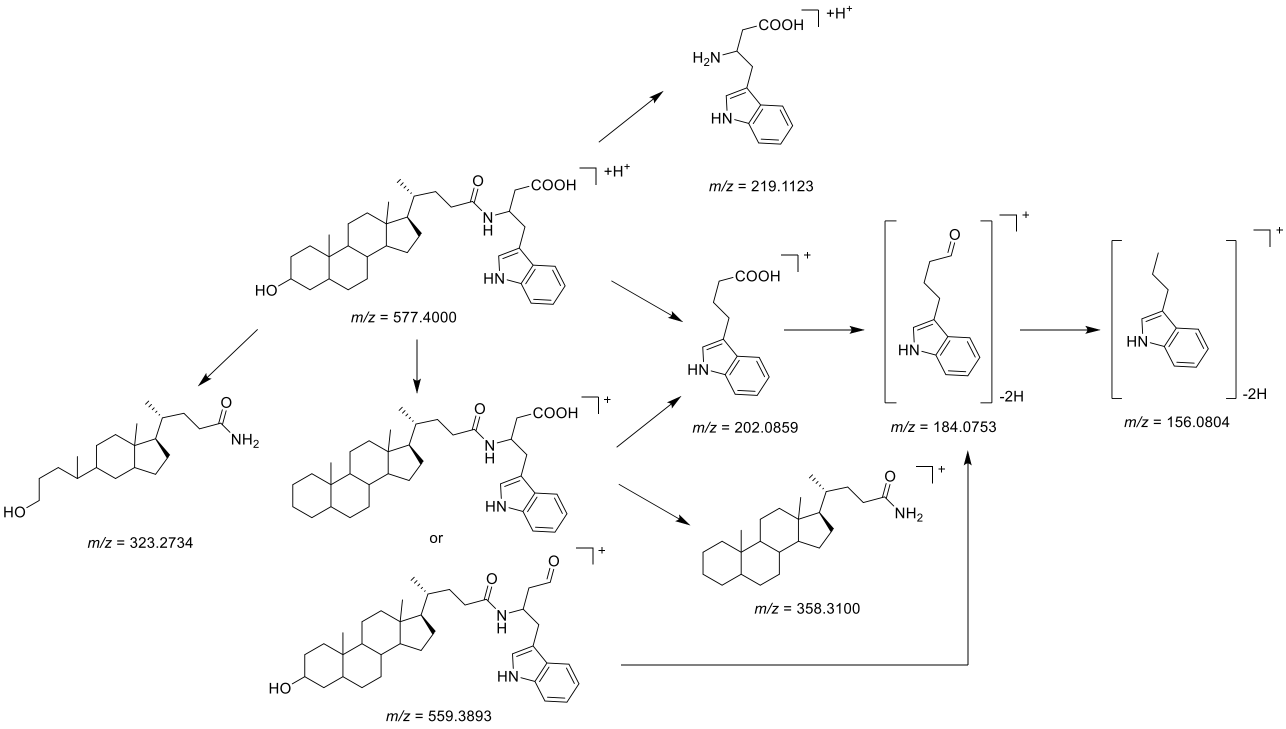

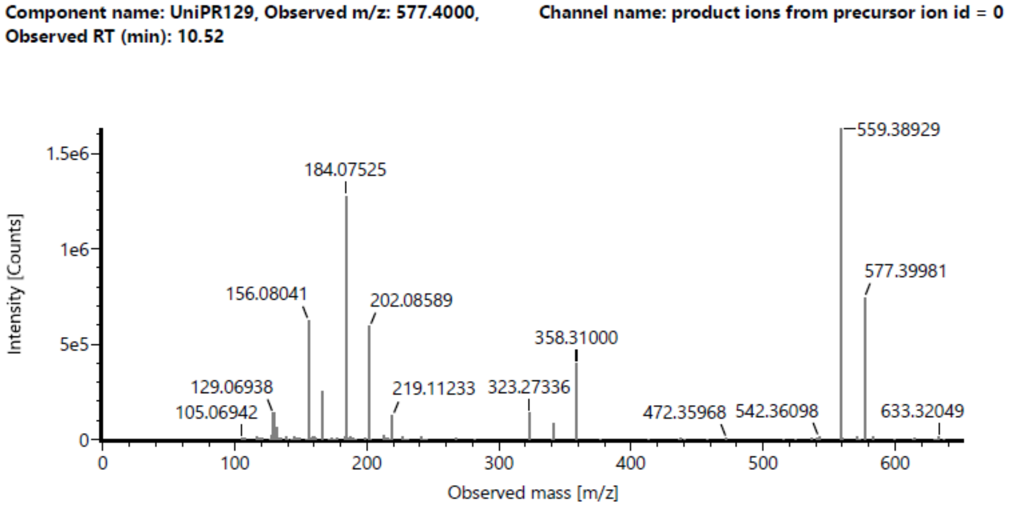

__Figure S2.__ a-d. Metabolite __M1__ in MLM.

a. Extracted ion chromatogram (XIC) in ESI$^+$ at m/z = 575.38 $[M+H]^+$ and RT = 10.64 min.

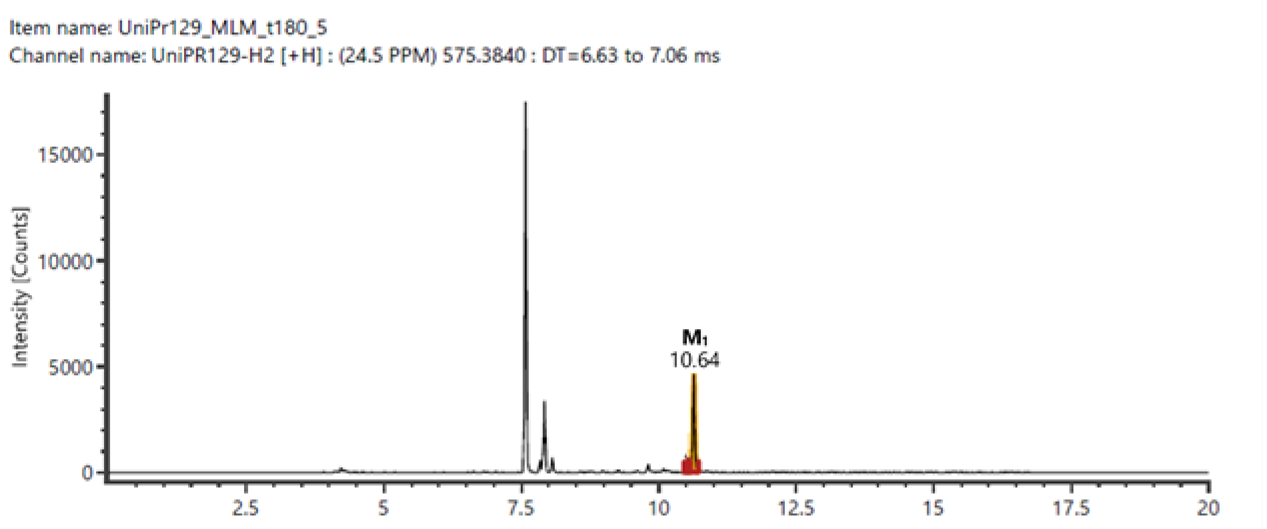

b. Experimental high resolution mass value for metabolite **M1** in ESI$^+$.

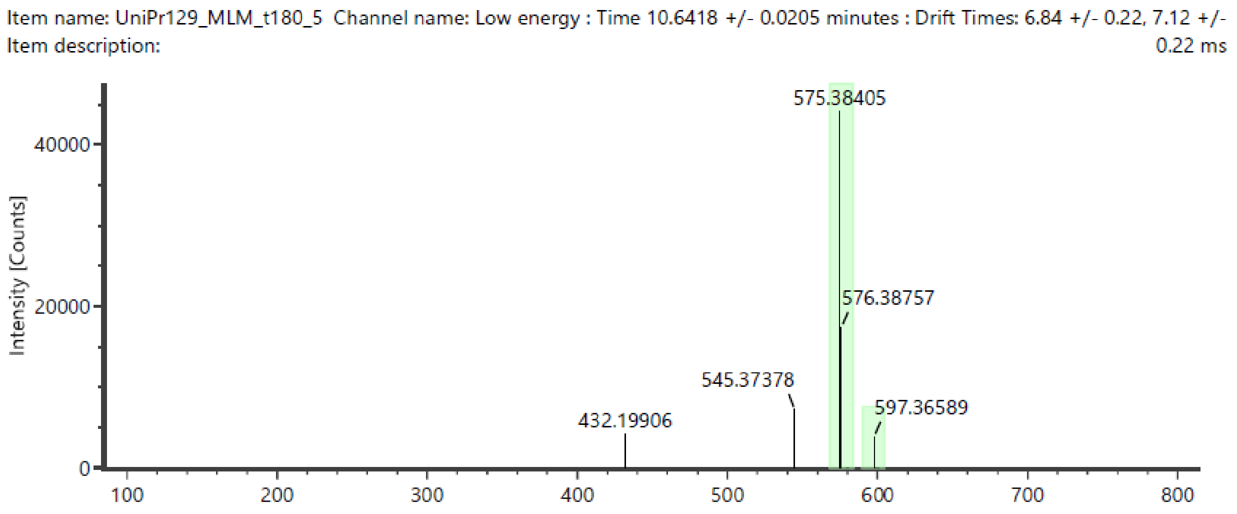

c. High resolution high energy (MS^E) MS spectrum in ESI$^+$ of **M1** together with tentative fragmentation pattern. In the MS/MS spectrum, peculiar fragments are present at *m/z* = 428.2, which is compatible with the loss of the 4-(indol-3-yl)-butanoyl fragment, and at *m/z* = 339.3, which likely derived from the further loss of the 3-aminopropanoyl group and the loss of the 3-keto group.

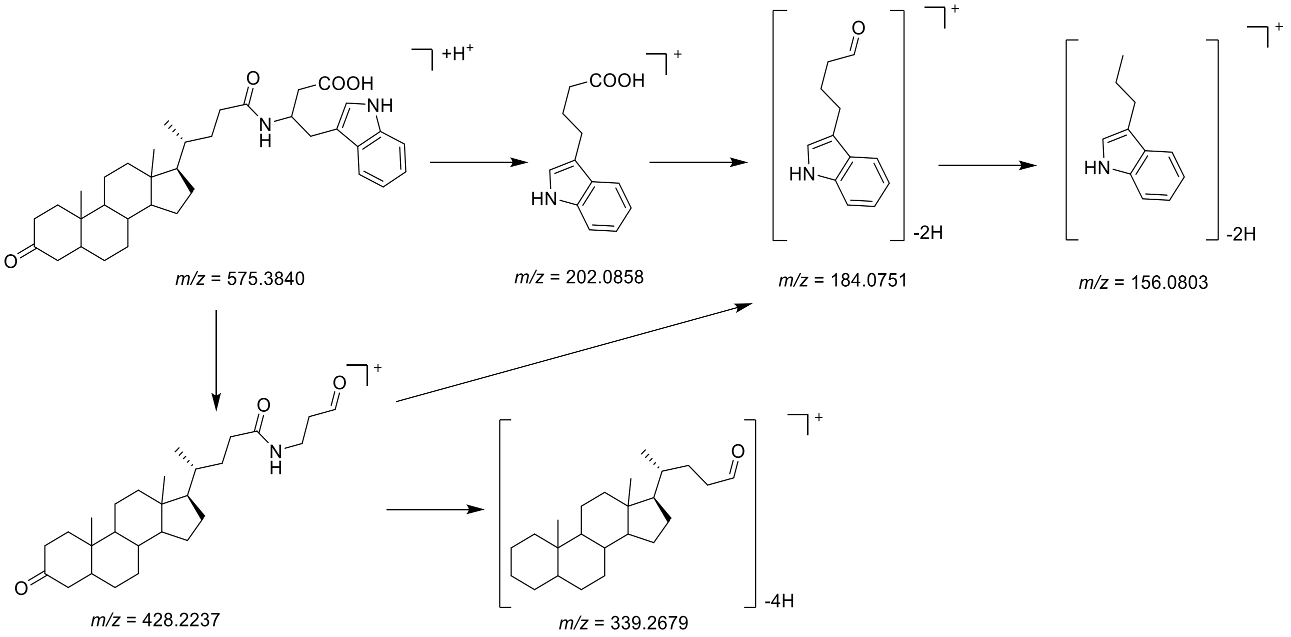

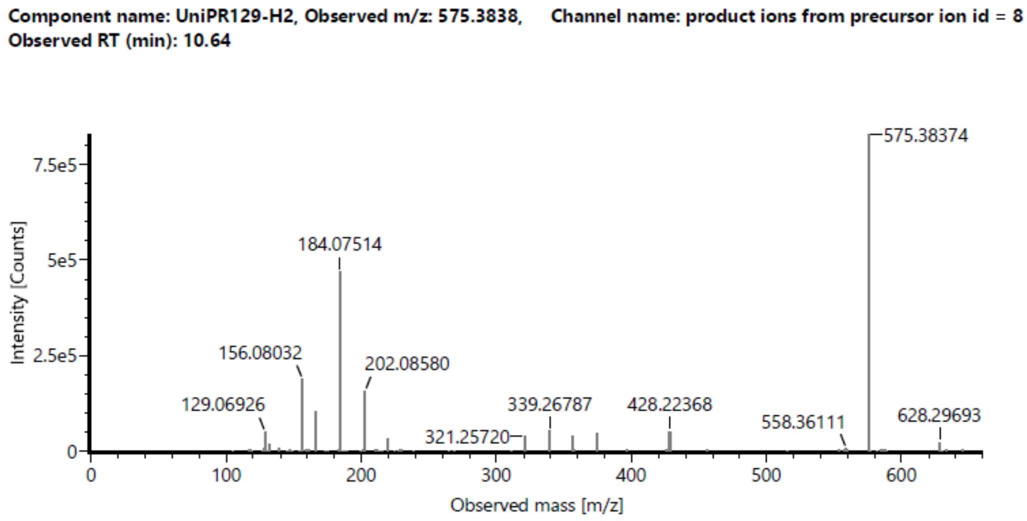

d. Metabolic soft spots on UniPR129 reported with a color-scale: the most probable sites of modification are highlighted in bright green. Based on the MS/MS spectrum of **M1**, UNIFI software returns as the most likely soft spots C-1-21 of the steroidal moiety; in particular, the oxidation of the 3-hydroxyl to a 3-keto group could be regarded as the most reasonable transformation.

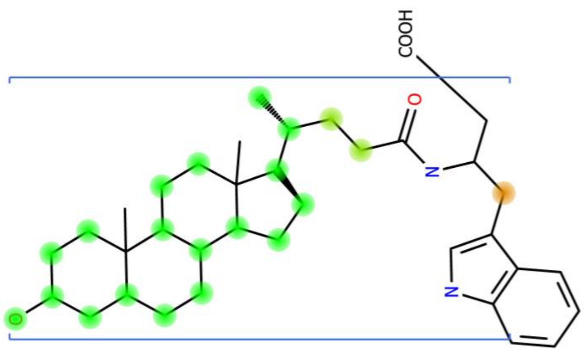

Figure S3. *a-d. Metabolite* ***M2*** *in MLM.*

a. Extracted ion chromatogram (XIC) in ESI+ at *m/z* = 593.39 [M+H]+ and RT = 7.58 min.

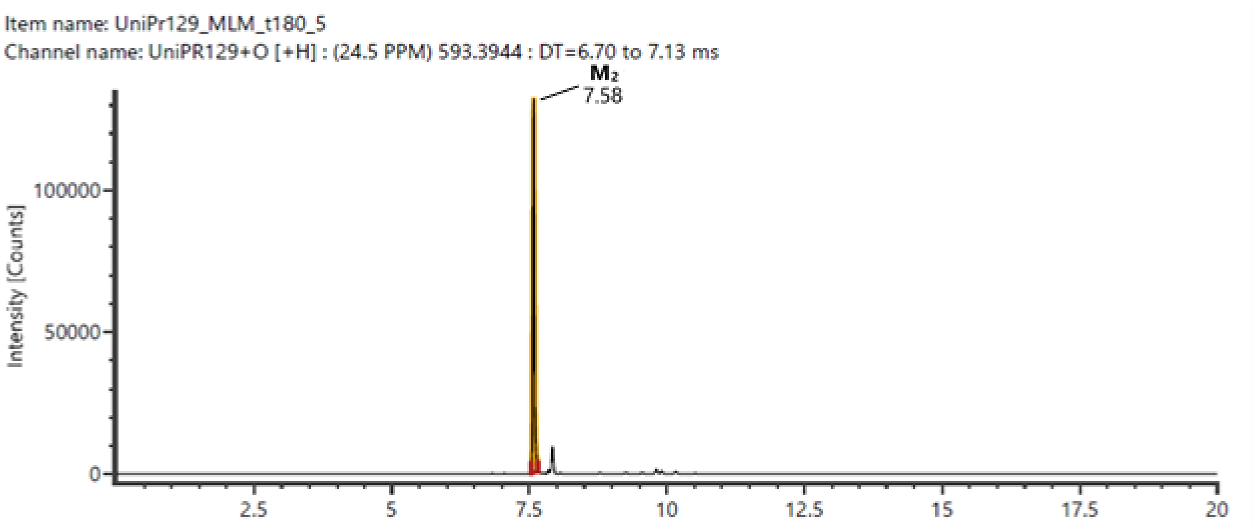

b. Experimental high resolution mass value for metabolite **M2** in ESI$^+$.

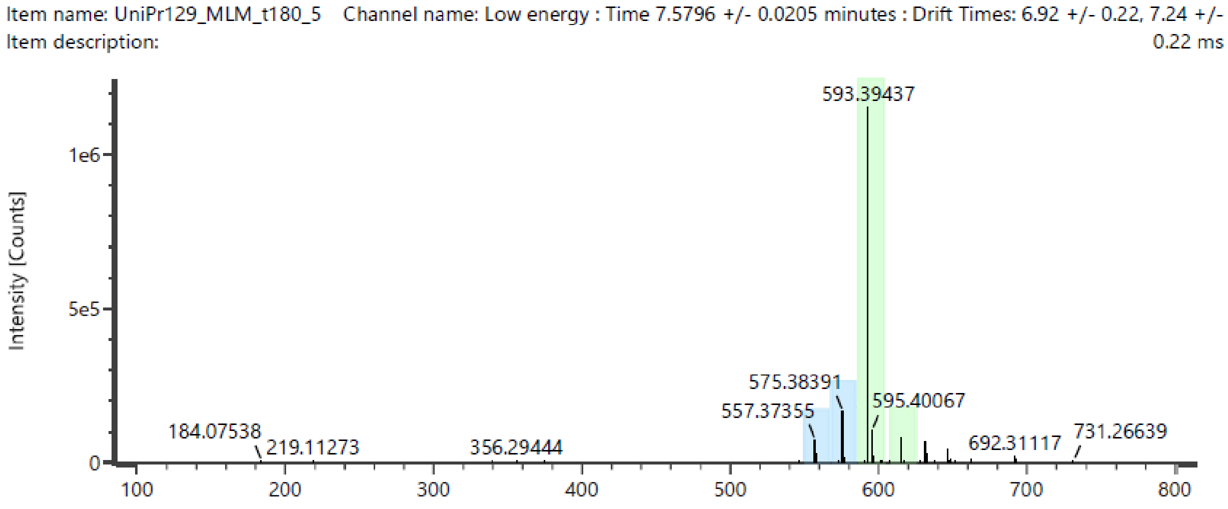

c. High resolution high energy (MS^E) MS spectrum in ESI$^+$ of **M2** together with tentative fragmentation pattern. The characteristic fragment ions are *m/z* = 575.4 and 557.4, which derived from sequential loss of a hydroxyl group (-16 with respect to 593.4) and water (-18 with respect to 575.4), respectively.

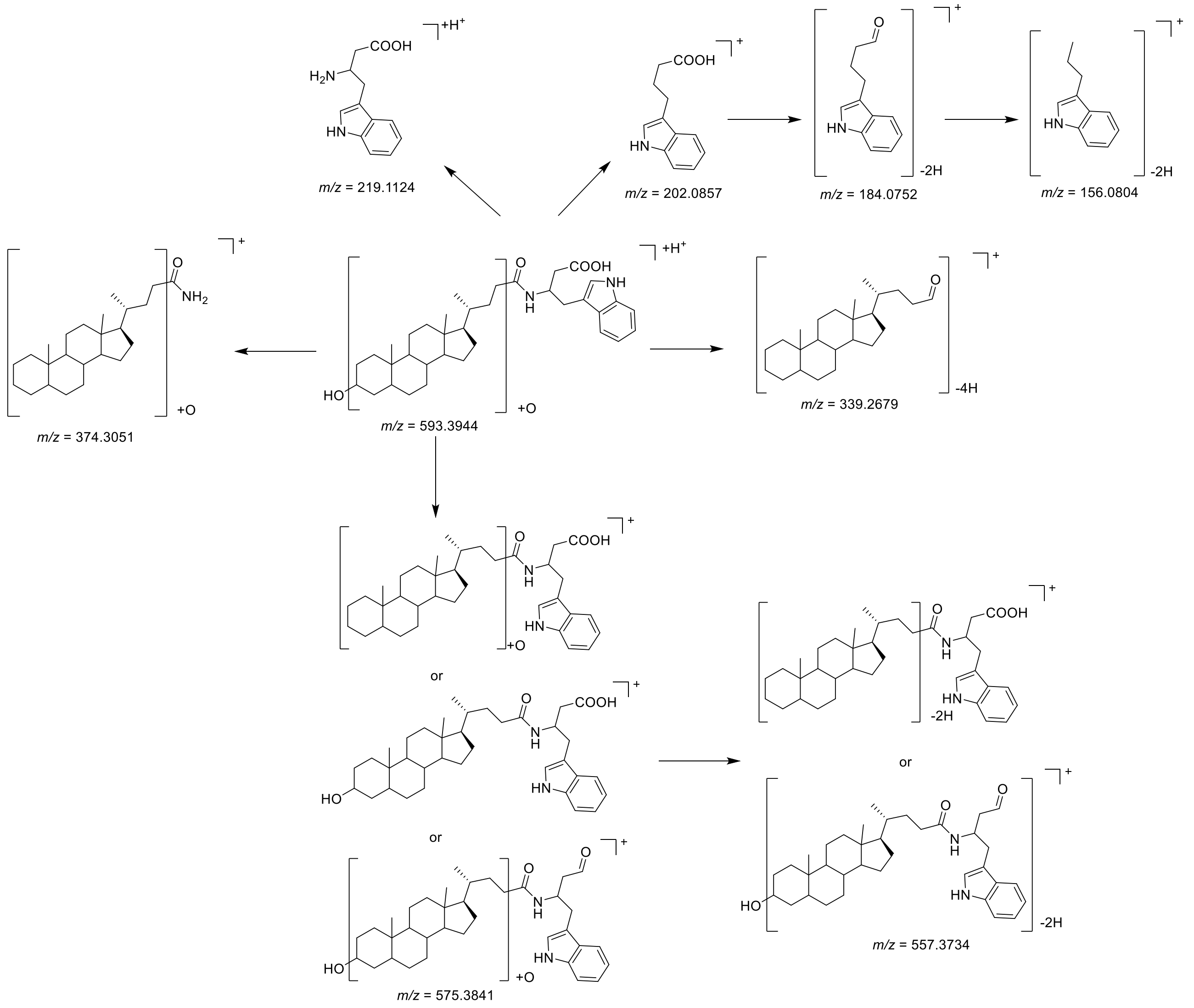

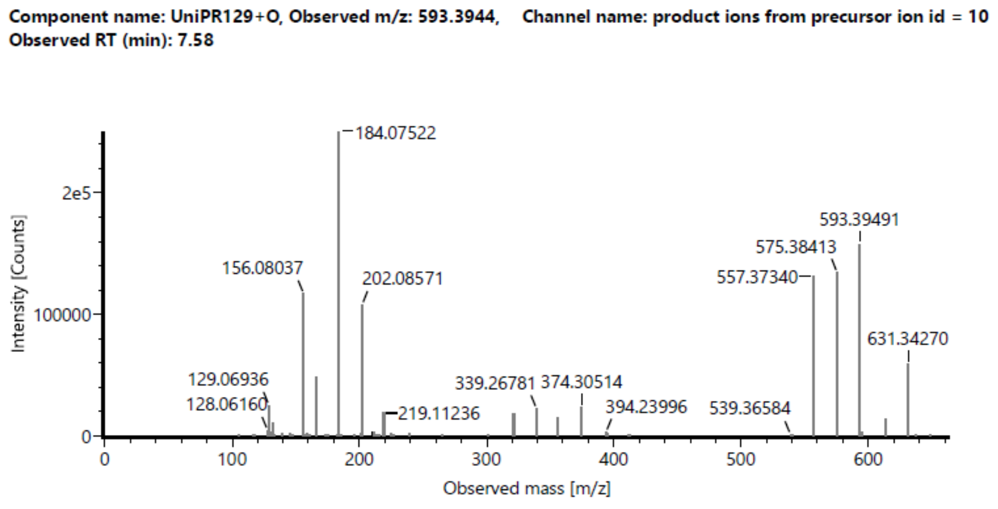

d. Metabolic soft spots on UniPR129 reported with a color-scale: the most probable sites of modification are highlighted in bright green. Based on the MS/MS spectrum of **M2**, UNIFI software returns as the most likely soft spots the C-atoms in the whole steroidal moiety except for C-4-7.

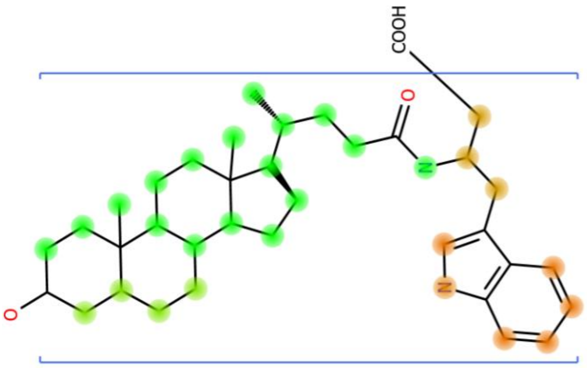

Figure S4. *a-d. Metabolite* ***M3*** *in MLM.*

a. Extracted ion chromatogram (XIC) in ESI$^+$ at m/z = 593.39 [M+H]$^+$ and RT = 7.69 min.

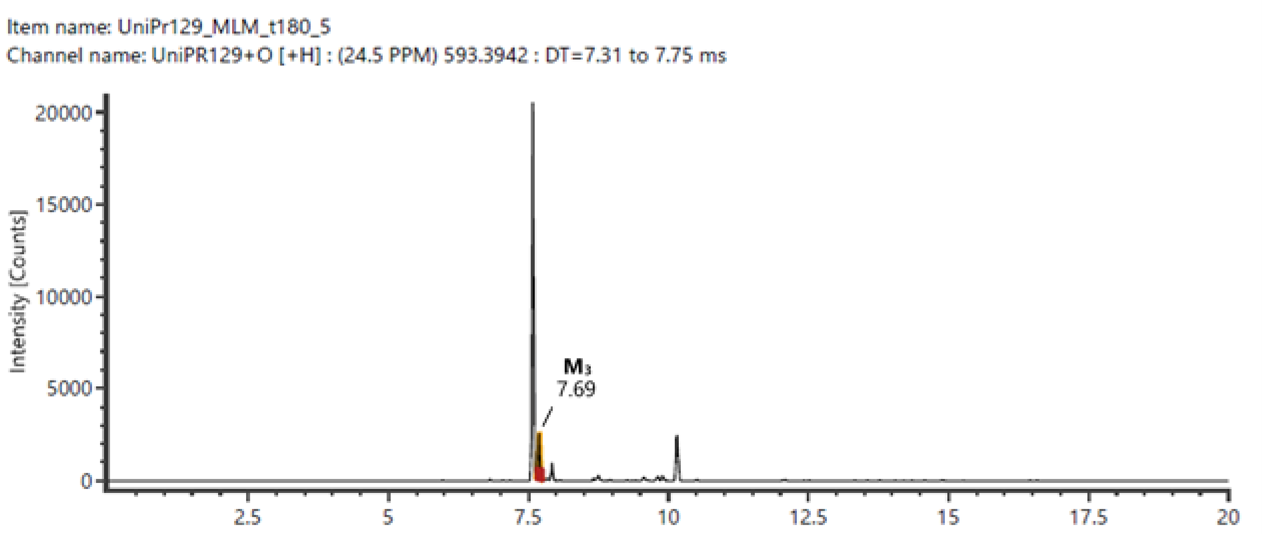

b. Experimental high resolution mass value for metabolite **M3** in ESI+.

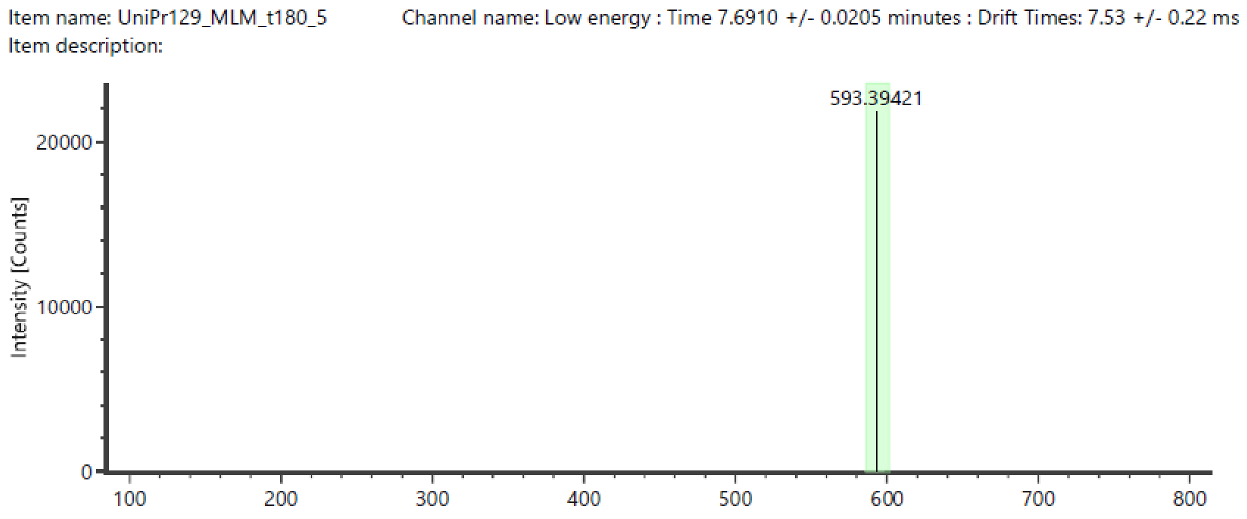

c. High resolution high energy (MSE) MS spectrum in ESI+ of **M3** together with tentative fragmentation pattern. **M3** shares the main product ions in the MS/MS spectrum (*m/z* = 557.4, 202.1, 184.1 and 156.1) with **M2**.

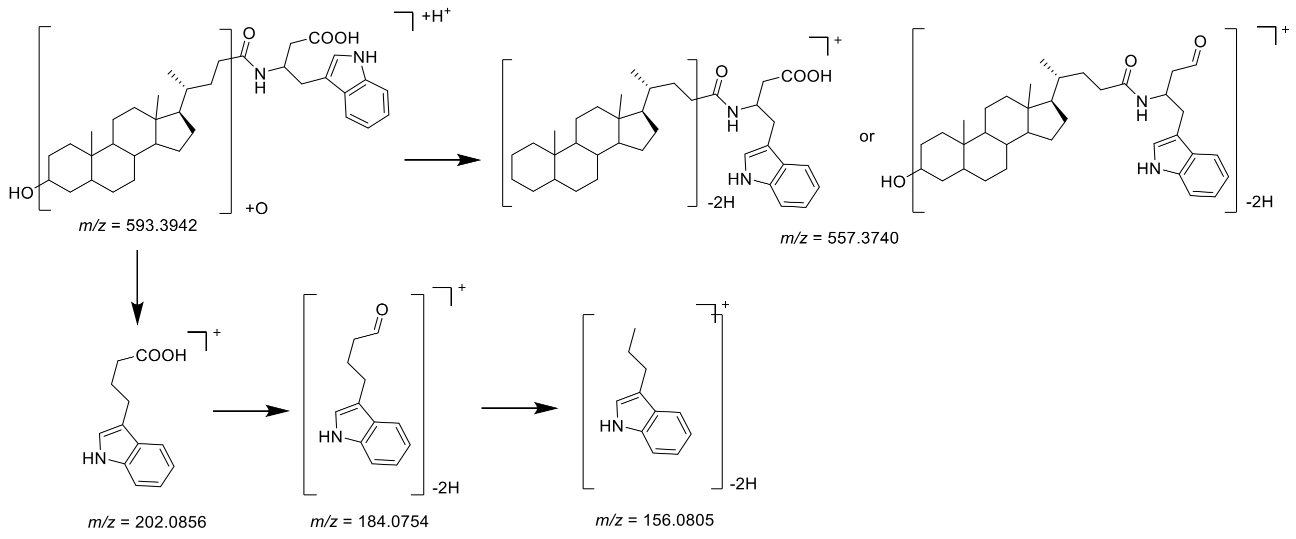

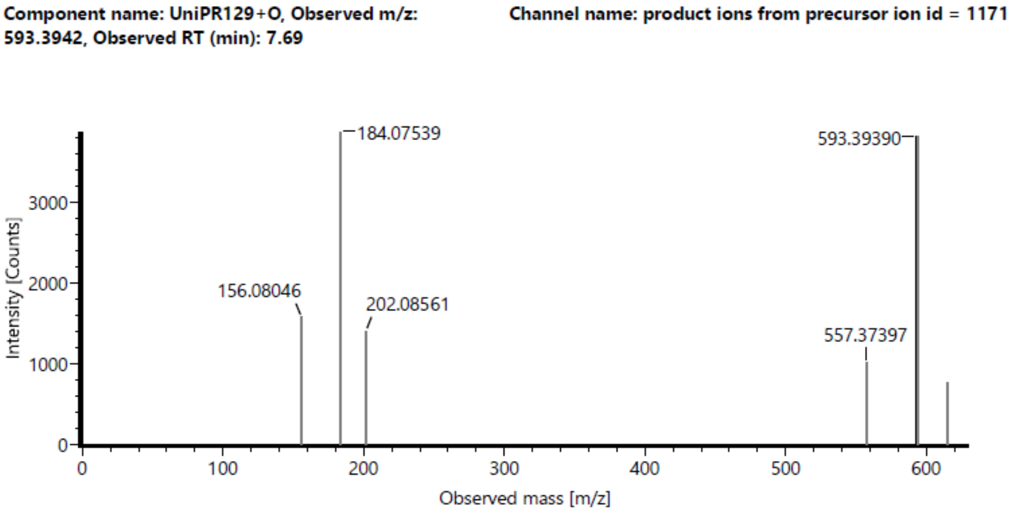

d. Metabolic soft spots on UniPR129 reported with a color-scale: the most probable sites of modification are highlighted in bright green. Based on the MS/MS spectrum of **M3**, UNIFI software returns as the most likely soft spots the C-atoms in the whole steroidal moiety.

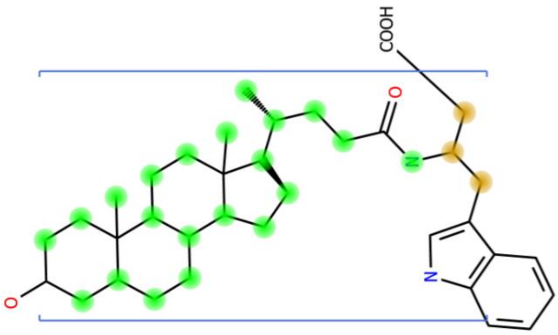

Figure S5. *a-d. Metabolite* ***M4*** *in MLM.*

a. Extracted ion chromatogram (XIC) in ESI$^+$ at m/z = 593.39 $[M+H]^+$ and RT = 7.84 min.

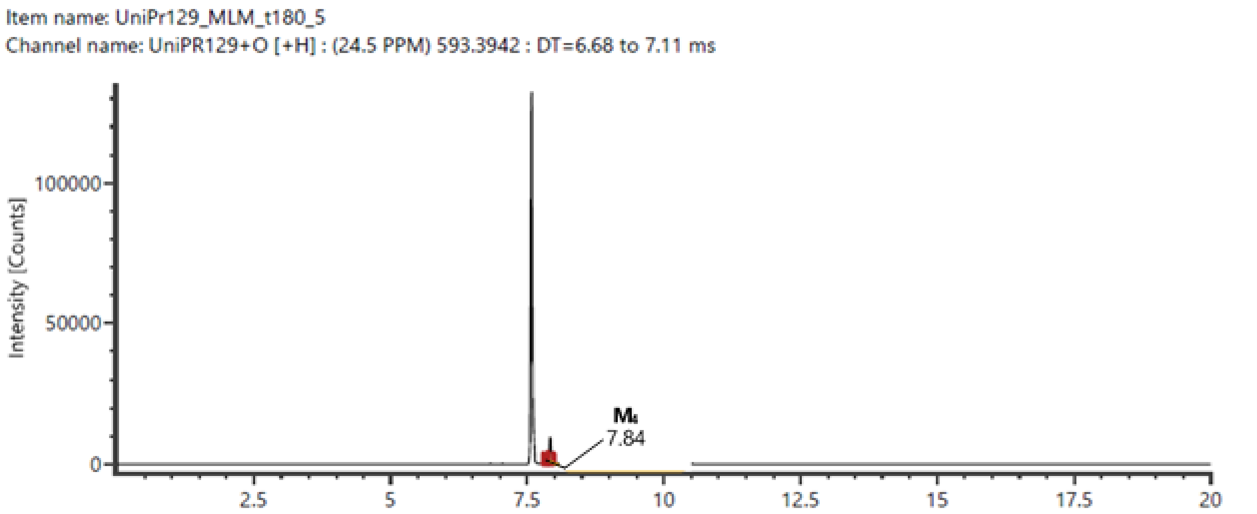

b. Experimental high resolution mass value for metabolite **M4** in ESI+.

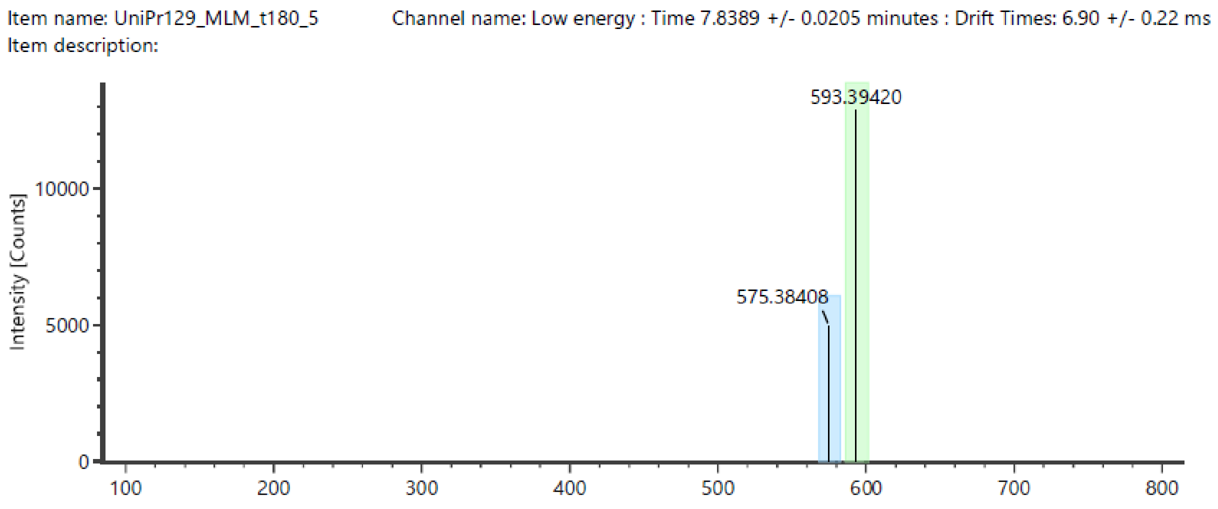

c. High resolution high energy (MS^E) MS spectrum in ESI+ of **M4** together with tentative fragmentation pattern. Product ions at *m*/*z* = 557.4 and 184.1 are the same reported for **M3** and **M2**.

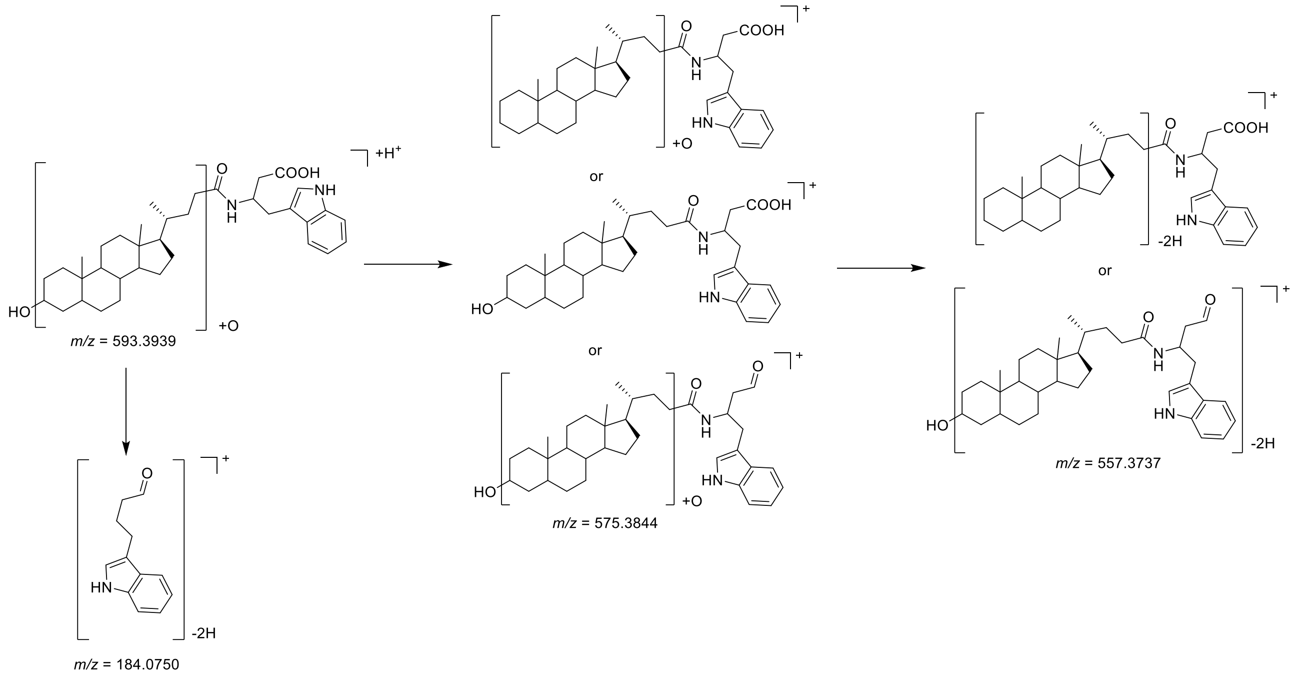

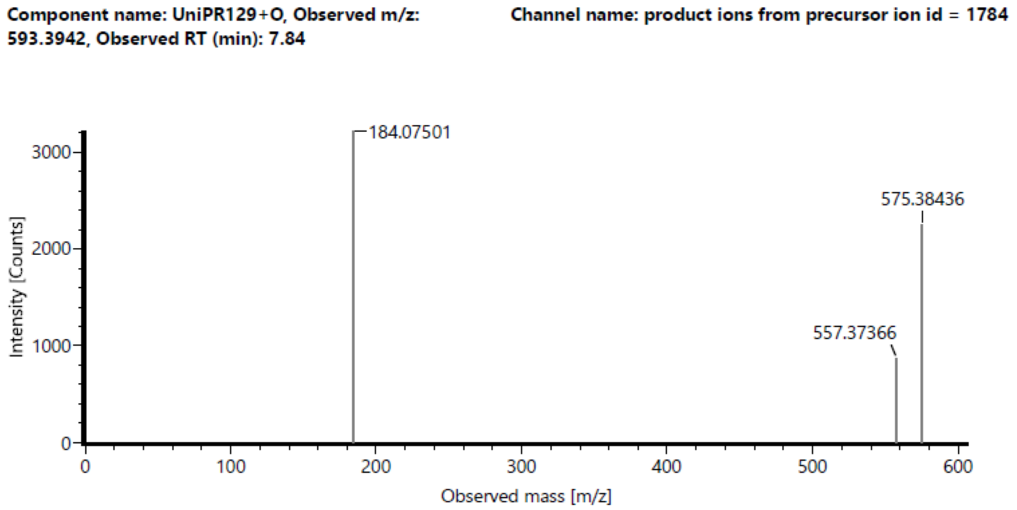

d. Metabolic soft spots on UniPR129 reported with a color-scale: the most probable sites of modification are highlighted in bright green. Based on the MS/MS spectrum of **M4**, UNIFI software returns as the most likely soft spots the C-atoms in the whole steroidal moiety, as for **M3**.

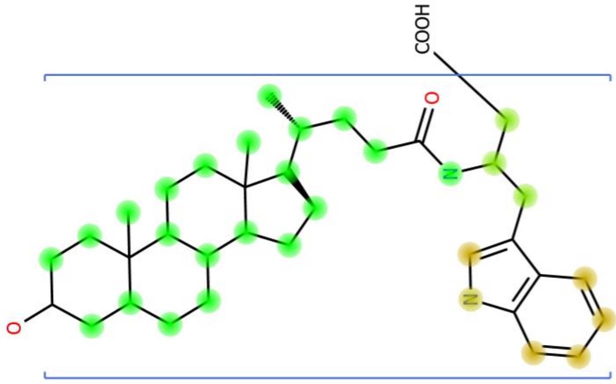

Figure S6. *a-d. Metabolite* ***M5*** *in MLM.*

a. Phase I metabolite **M5** in mouse liver microsomes. Extracted ion chromatogram (XIC) in ESI$^+$ at m/z = 593.39 [M+H]$^+$ and RT = 7.92 min.

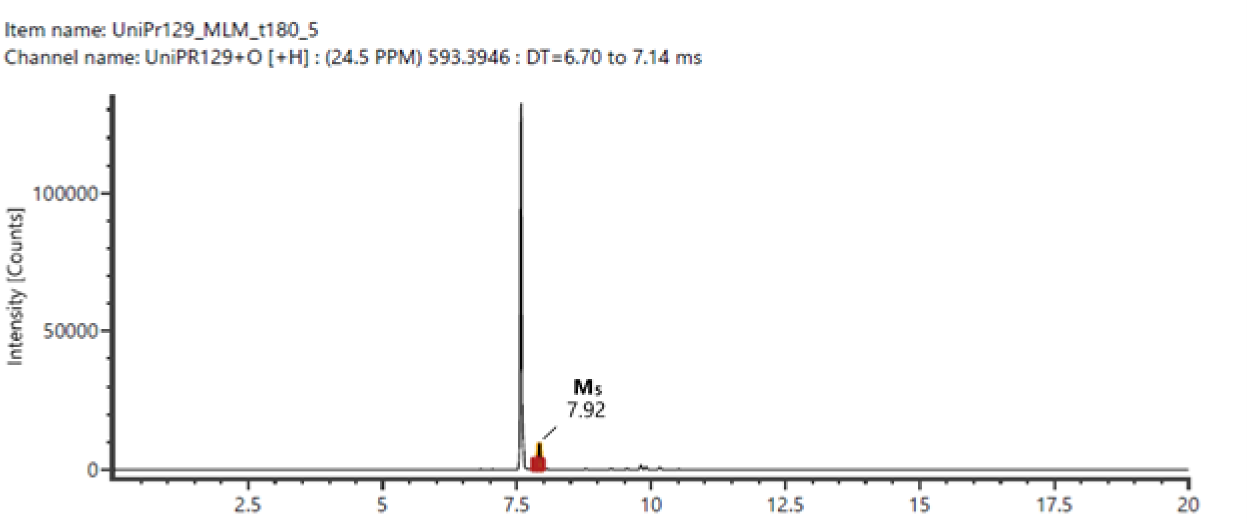

b. Experimental high resolution mass value for metabolite **M5** in ESI+.

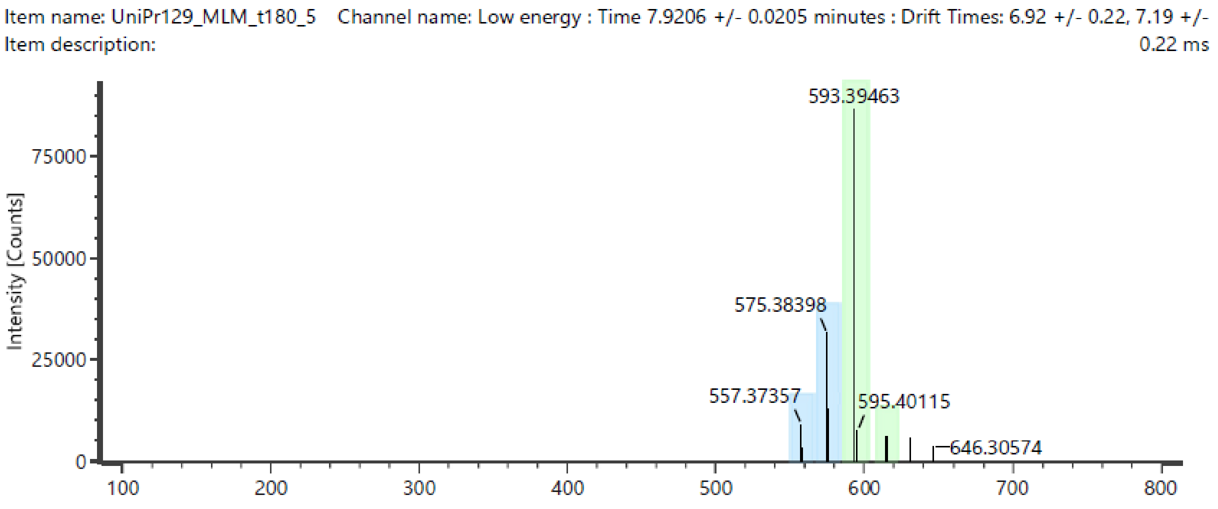

c. High resolution high energy (MSE) MS spectrum in ESI+ of **M5** together with tentative fragmentation pattern. All fragment ions in the MS/MS spectra have already been reported for **M2**.

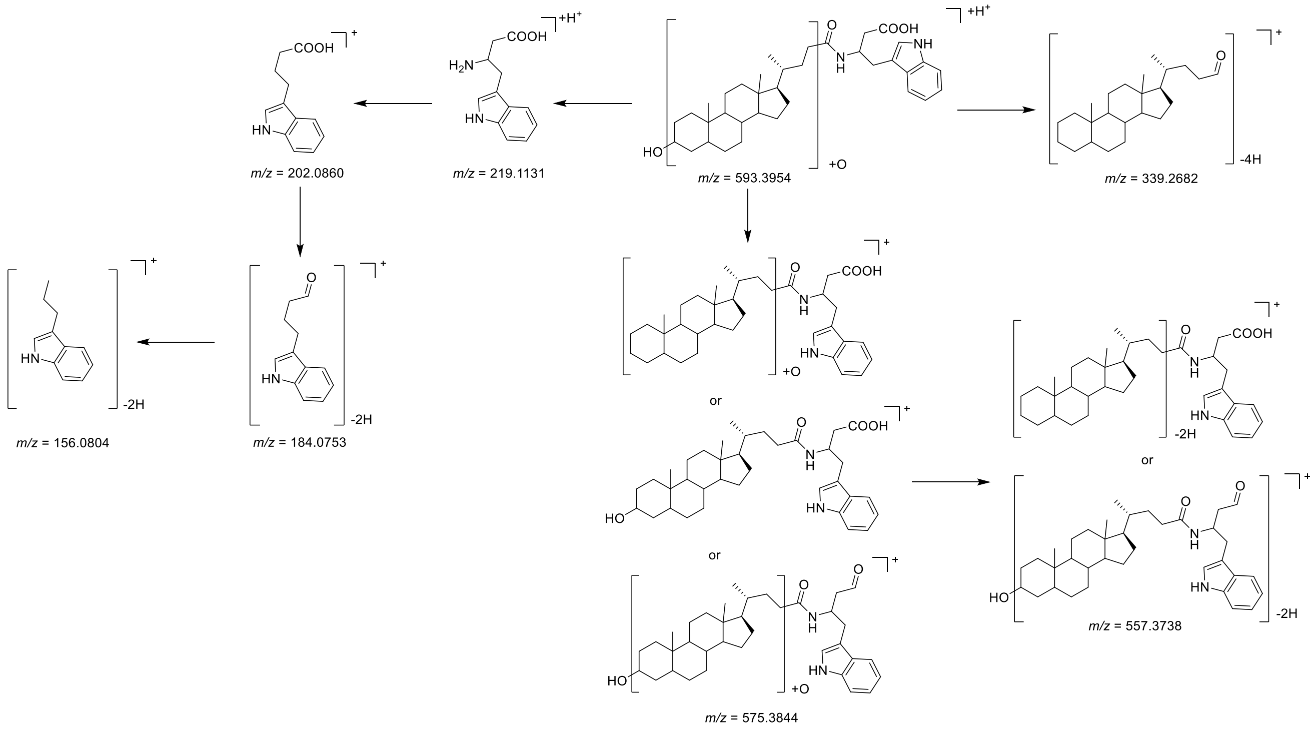

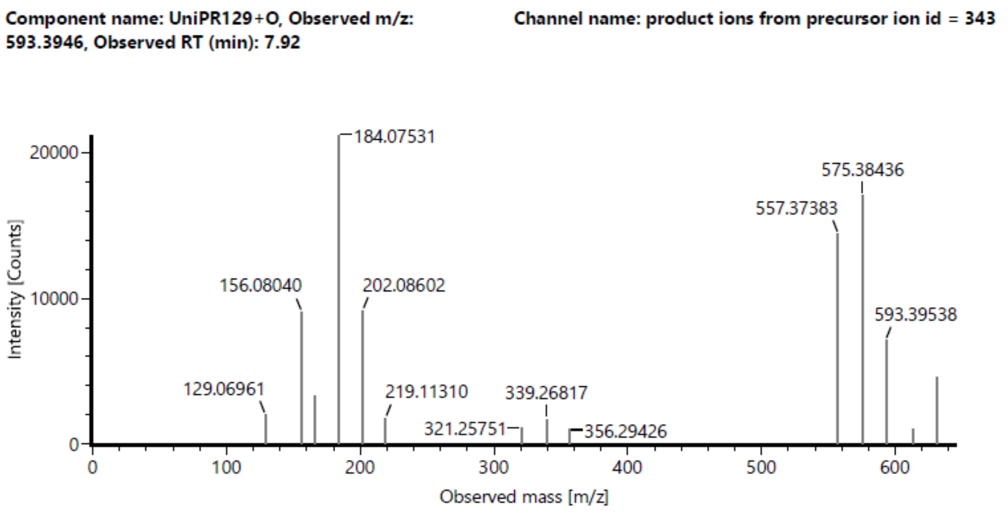

d. Metabolic soft spots on UniPR129 reported with a color-scale: the most probable sites of modification are highlighted in bright green. Based on the MS/MS spectrum of **M2**, UNIFI software returns as the most likely soft spots C-atoms in the whole steroidal moiety with the exception of C-4-7, C-22 and C-23.

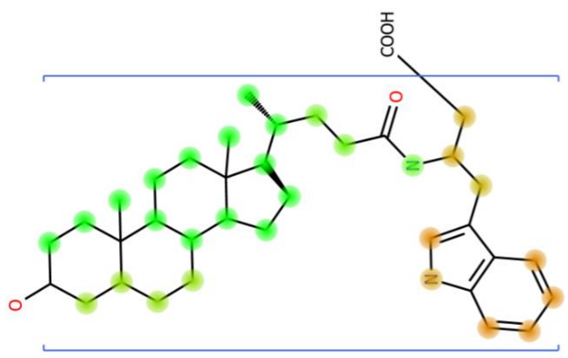

Figure S7. *a-d. Metabolite **M6** in MLM.*

a. Phase I metabolite **M6** in mouse liver microsomes. Extracted ion chromatogram (XIC) in ESI$^+$ at m/z = 593.39 [M+H]$^+$ and RT = 9.81 min.

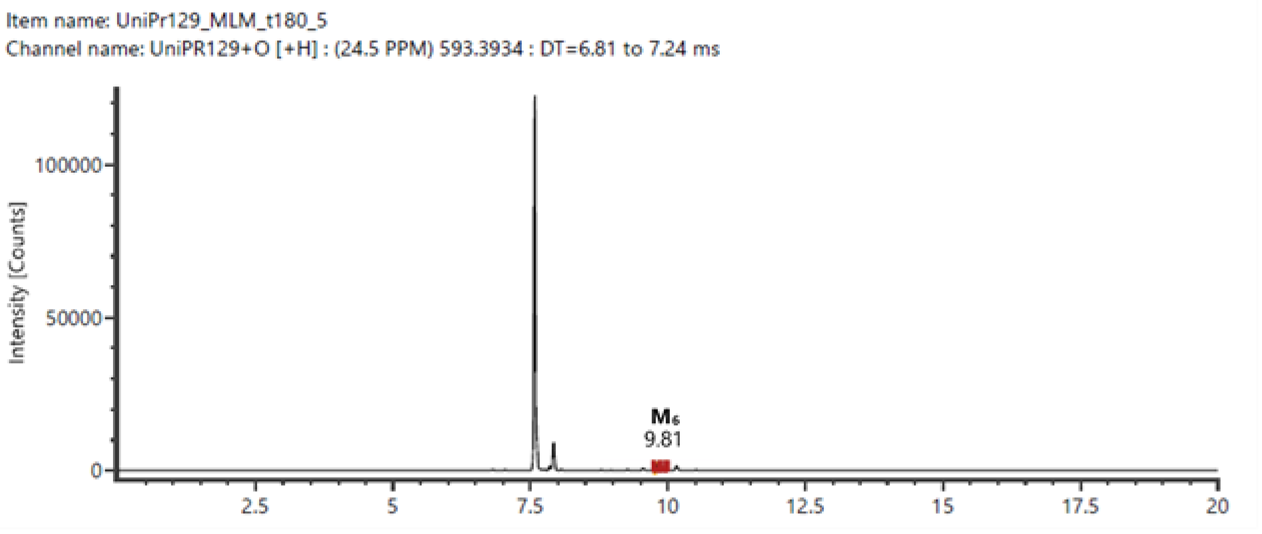

b. Experimental high resolution mass value for metabolite **M6** in ESI⁺.

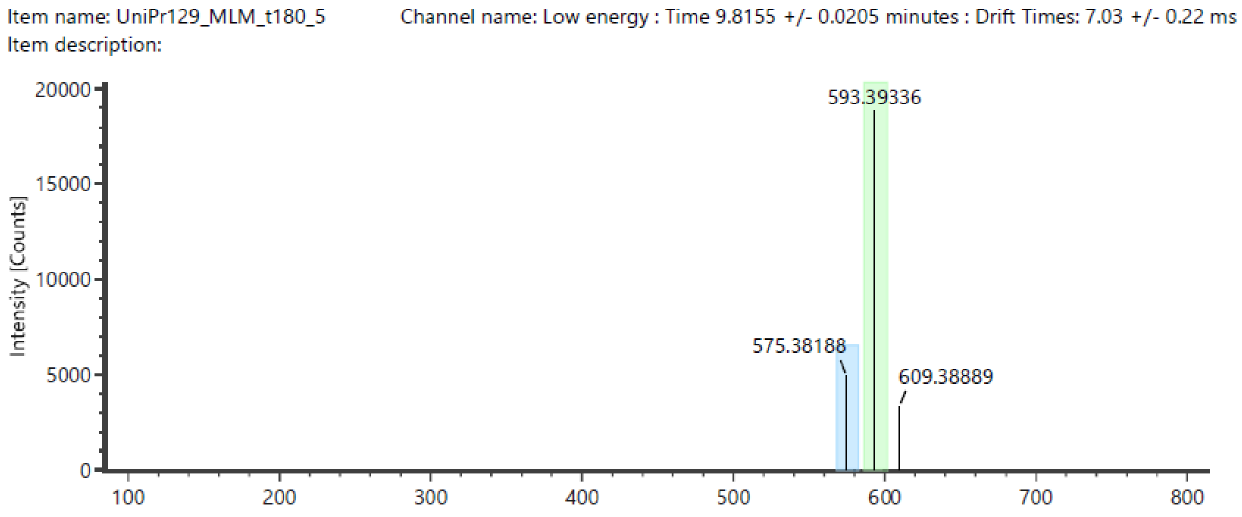

c. High resolution high energy (MS^E) MS spectrum in ESI⁺ of **M6** together with tentative fragmentation pattern. In addition to the product ion at *m/z* = 557.4, *m/z* = 217.1 consist of the L-β-homotryptophan moiety and *m/z* = 199.1 derived from the previous ion after loss of water (-18).

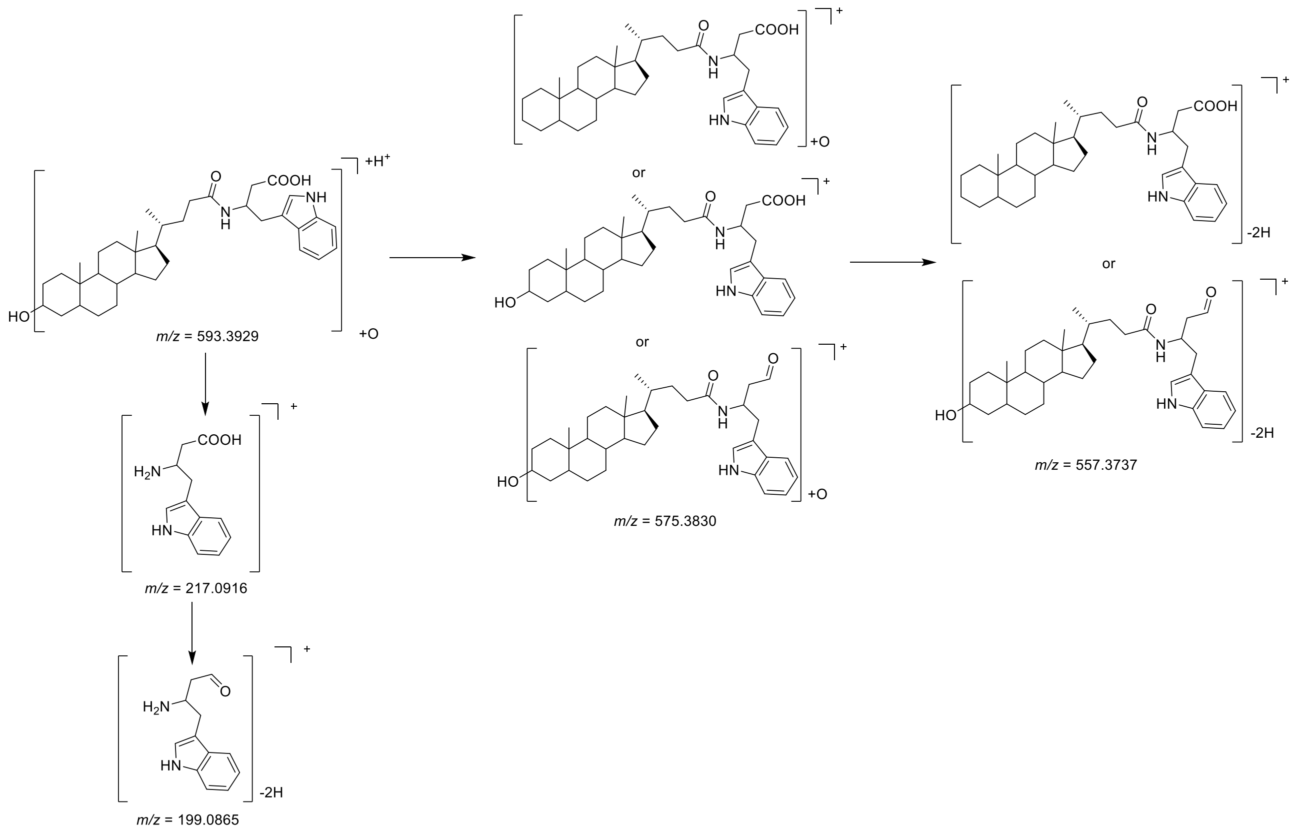

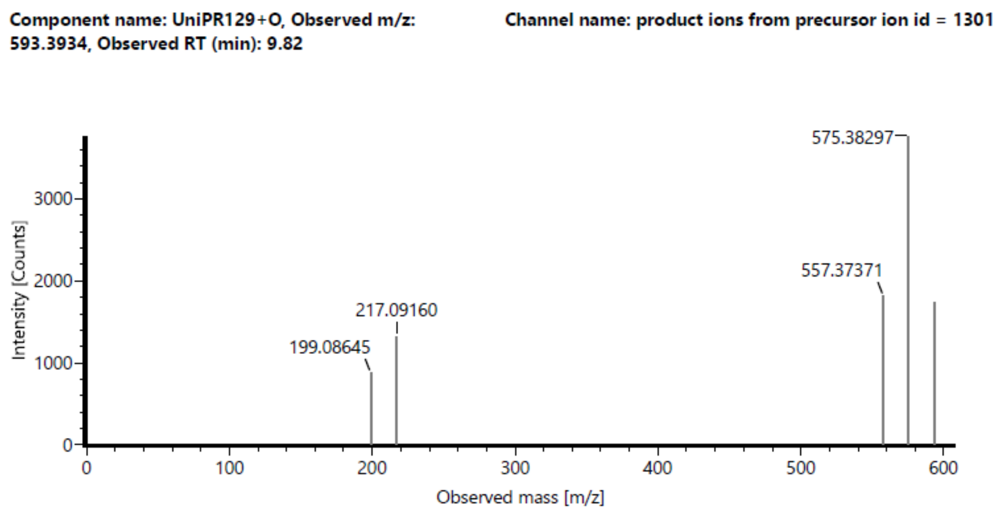

d. Metabolic soft spots on UniPR129 reported with a color-scale: the most probable sites of modification are highlighted in bright green. Based on the MS/MS spectrum of **M6**, UNIFI software returns as the most likely soft spots the same C-atoms reported for **M3** and **M4**.

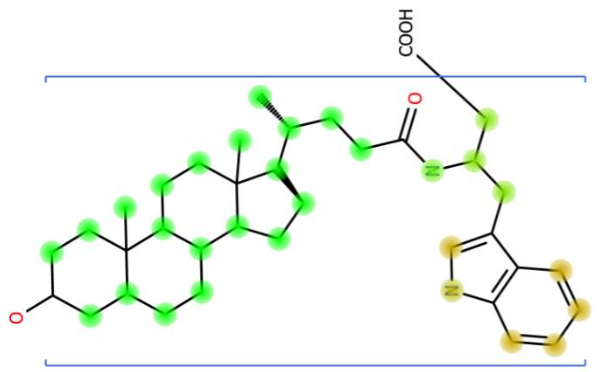

Figure S8. a-d. Metabolite ***M7*** *in MLM.*

a. Phase I metabolite **M7** in mouse liver microsomes. Extracted ion chromatogram (XIC) in ESI$^+$ at *m/z* = 593.39 $[M+H]^+$ and RT = 10.16 min.

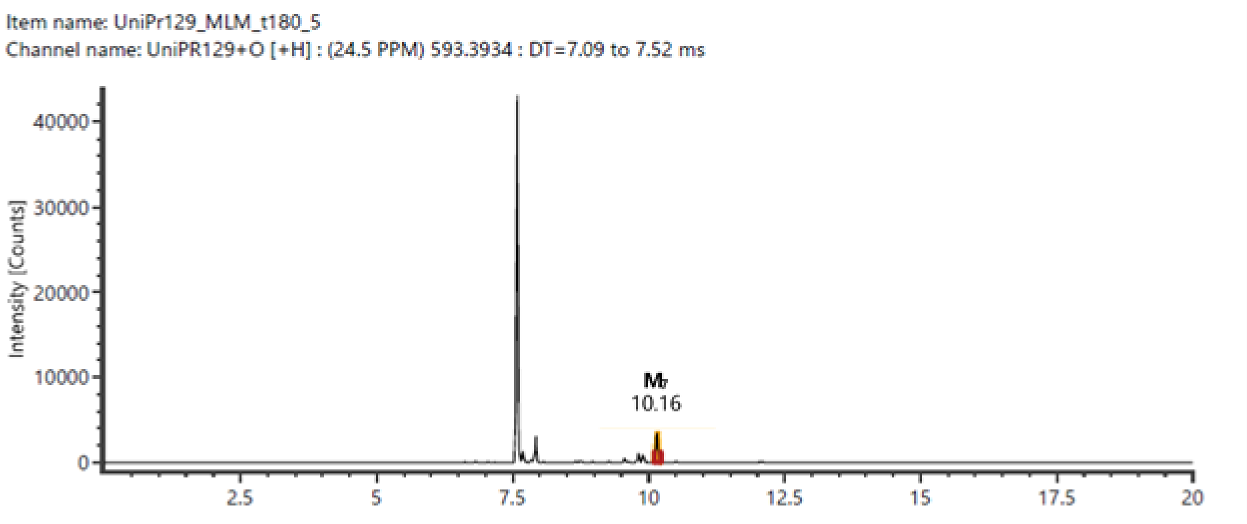

b. Experimental high resolution mass value for metabolite **M7** in ESI$^+$.

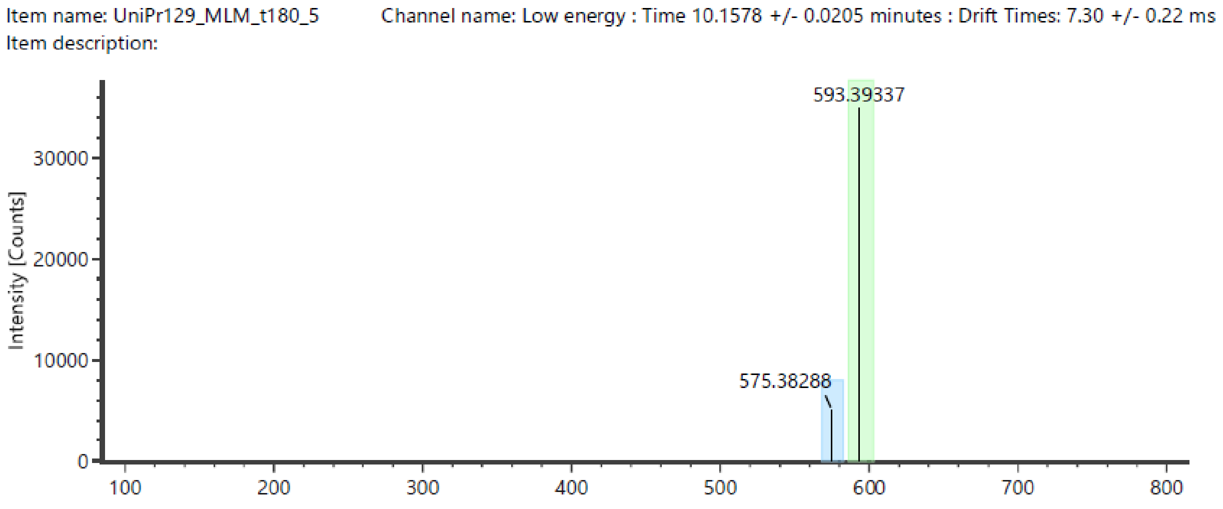

c. High resolution high energy (MS^E) MS spectrum in ESI^+ of **M7** together with tentative fragmentation pattern. Ion fragment at *m/z* = 557.4 has been found in all previously reported M+O metabolites and ion product at *m/z* = 217.1 is the same reported for **M6**. A peculiar peak at *m/z* = 172.1, however, could account for the oxidation of the propyl-indolic moiety.

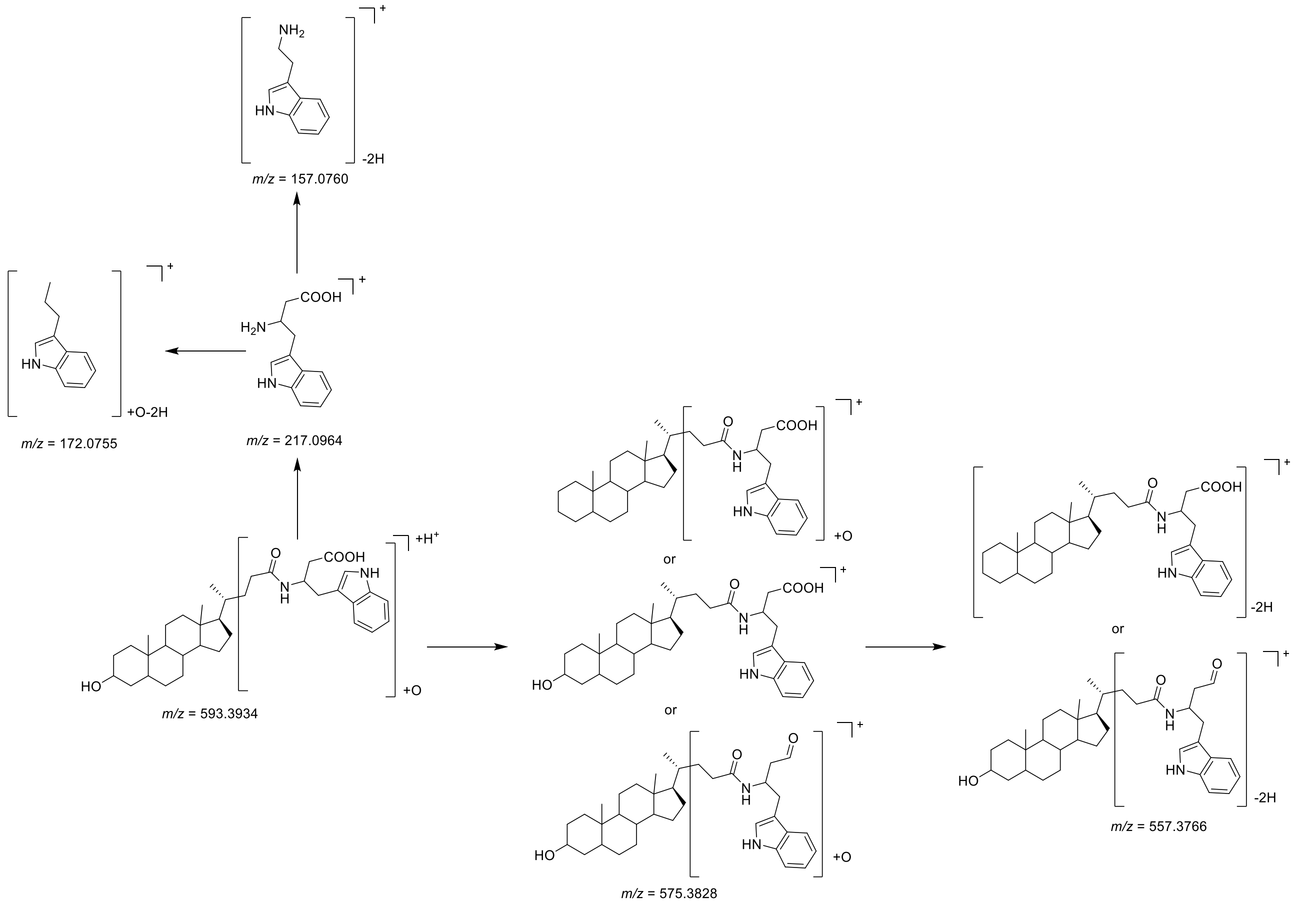

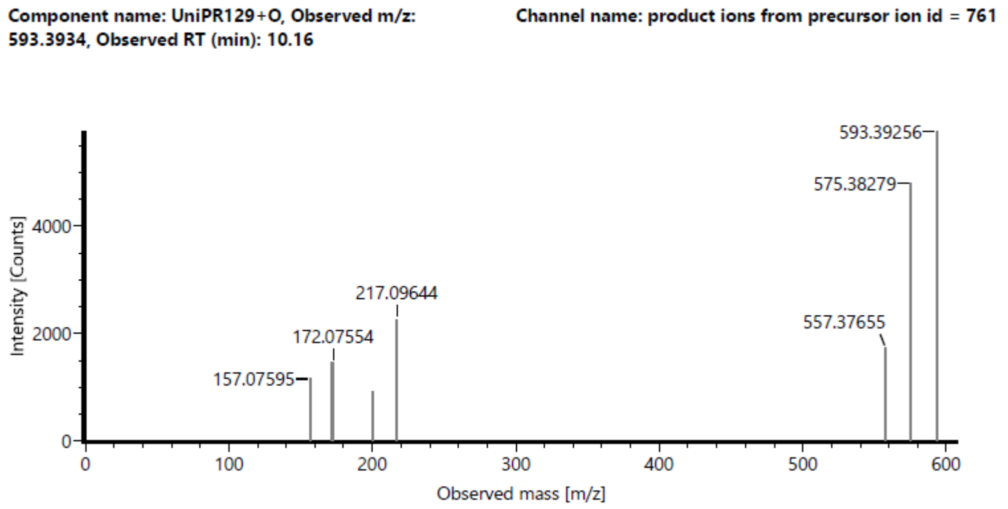

d. Metabolic soft spots on UniPR129 reported with a color-scale: the most probable sites of modification are highlighted in bright green. Based on the MS/MS spectrum of **M7**, UNIFI software returns as the most likely soft spot the C-atom of the propyl-indolic moiety of UniPR129.

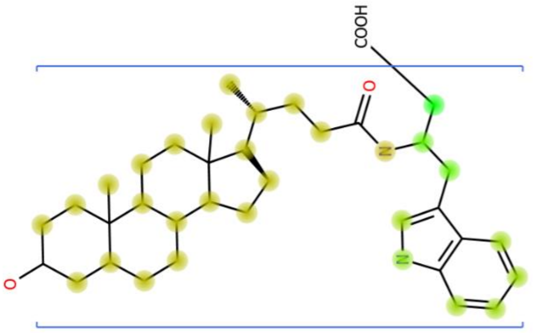

Figure S9. *a-c. Metabolite **M8** in MLM.*

a. Phase I metabolite **M8** in mouse liver microsomes. Extracted ion chromatogram (XIC) in ESI$^+$ at m/z = 609.39 [M+H]$^+$ and RT = 6.82 min.

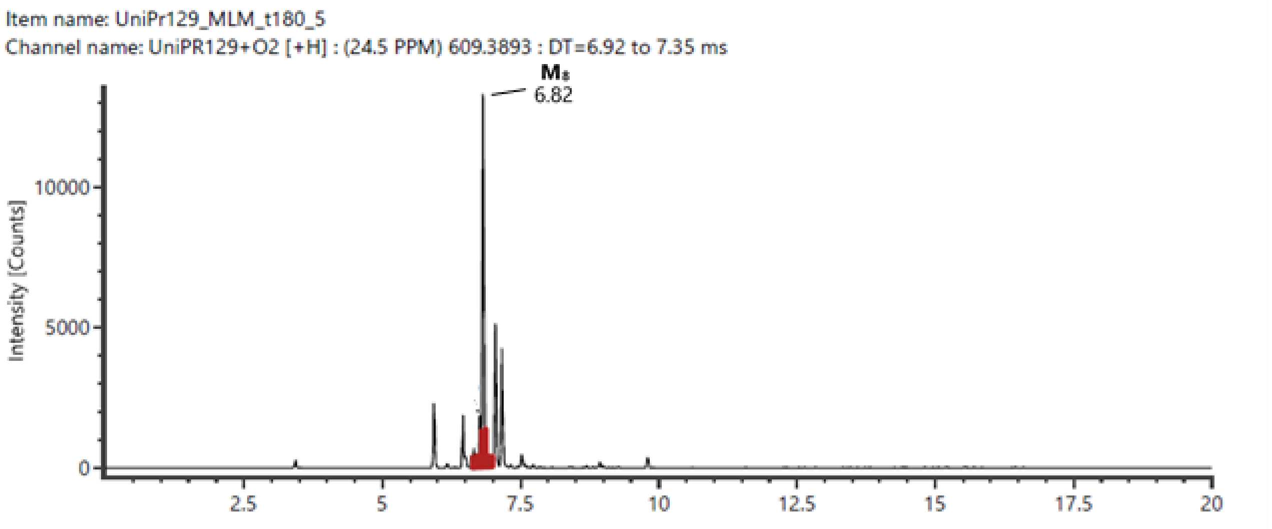

b. Experimental high resolution mass value for metabolite **M8** in ESI+.

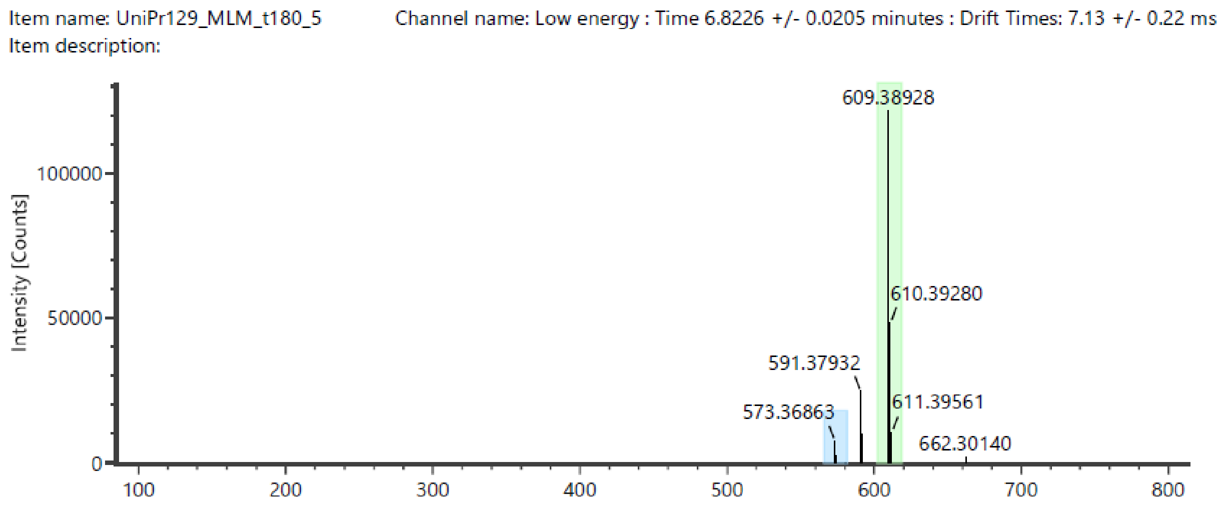

c. High resolution high energy (MS^E) MS spectrum in ESI+ of **M8** together with tentative fragmentation pattern. MS/MS spectrum shows a base peak at *m/z* = 591.4, compatible with the loss of water (-18); other characteristic peaks are at *m/z* = 573.4 (further dehydration, -18) and at *m/z* = 555.36 (loss of three water molecules with respect to *m/z* = 609.36, -54). Fragment ions at *m/z* = 172.1 and at *m/z* = 235.1 could account for a double oxidation on the L-β-homotryptophan; however, UNIFI software could not predict likely sites for oxidation.

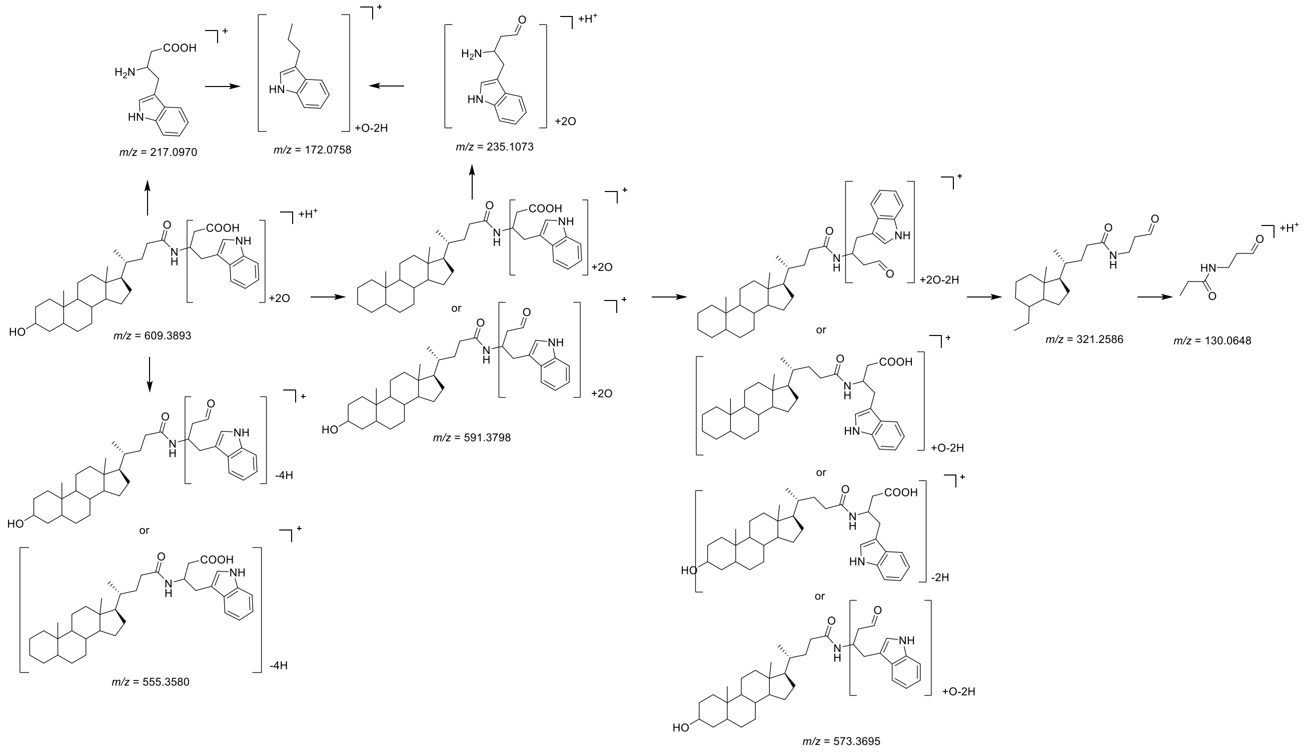

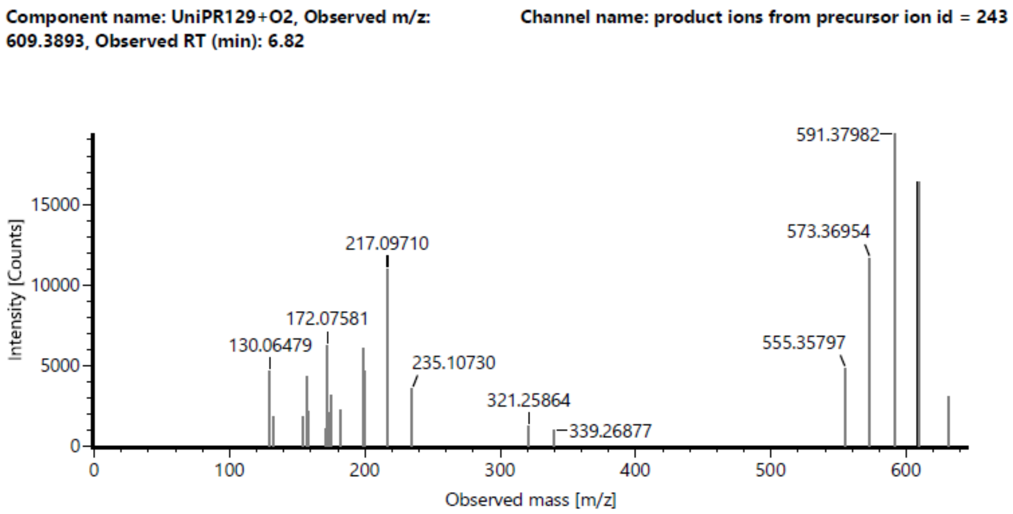

Figure S10. *a-c. Metabolite* ***M9*** *in MLM.*

a. Phase I metabolite **M9** in mouse liver microsomes. Extracted ion chromatogram (XIC) in ESI$^+$ at *m/z* = 609.39 $[M+H]^+$ and RT = 7.05 min.

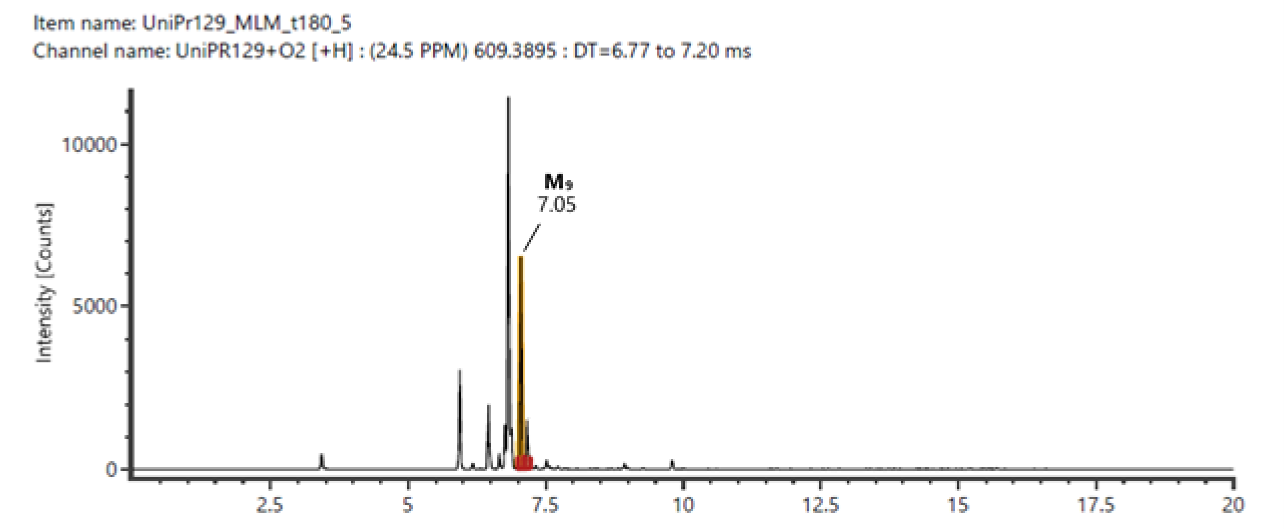

b. Experimental high resolution mass value for metabolite **M9** in ESI+.

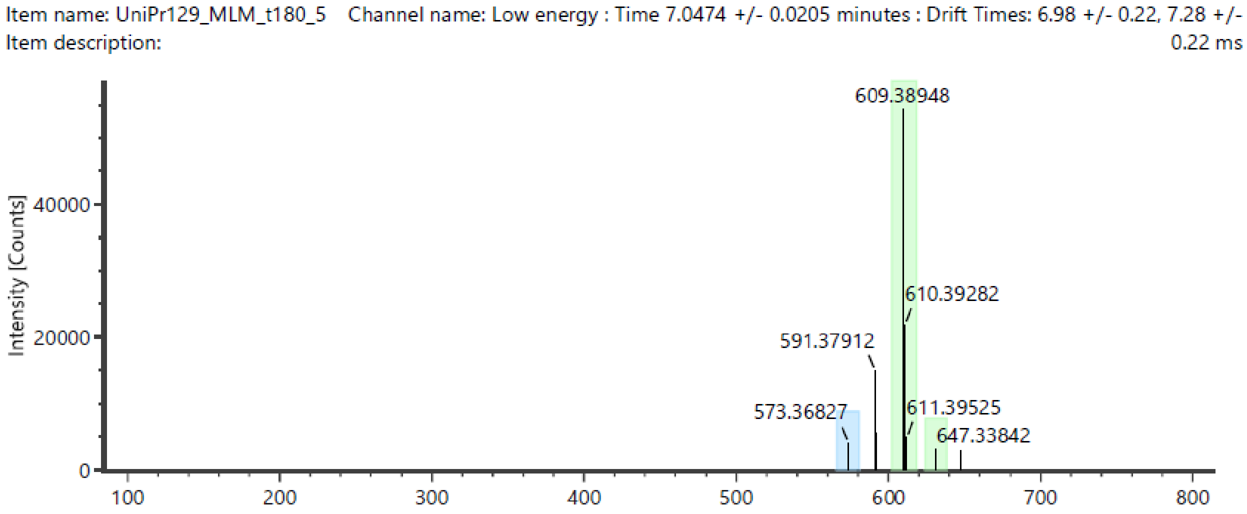

c. High resolution high energy (MS^E) MS spectrum in ESI+ of **M9** together with tentative fragmentation pattern. In the MS/MS spectrum, product ions at *m/z* = 573.4 and 555.4 are compatible with the loss of two and three water molecules, respectively. Despite no fragment suggests oxidation to occur on the L-β-homotryptohan portion, in contrast with **M8**, UNIFI software cannot return the most likely sites of oxidation on the steroidal moiety.

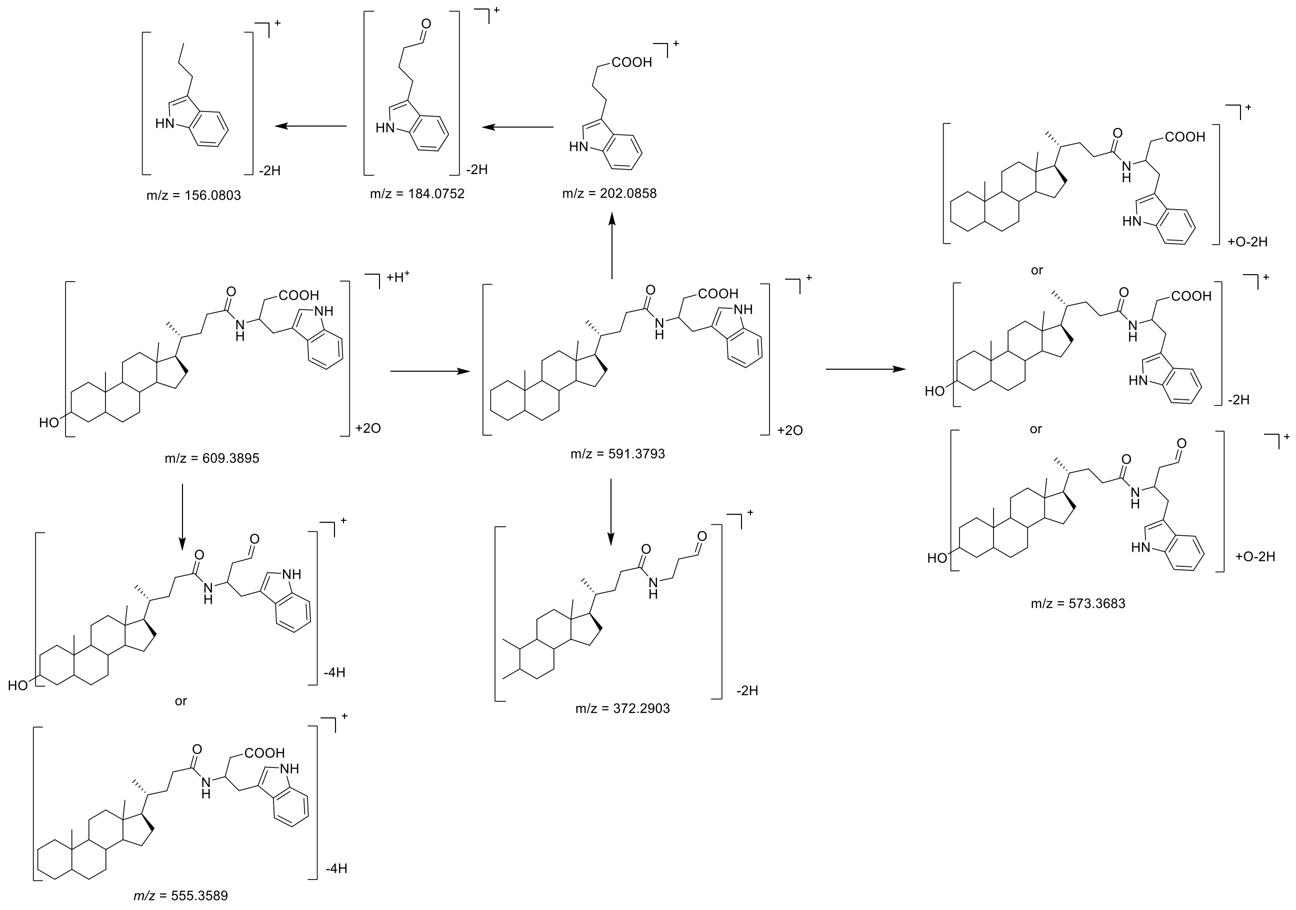

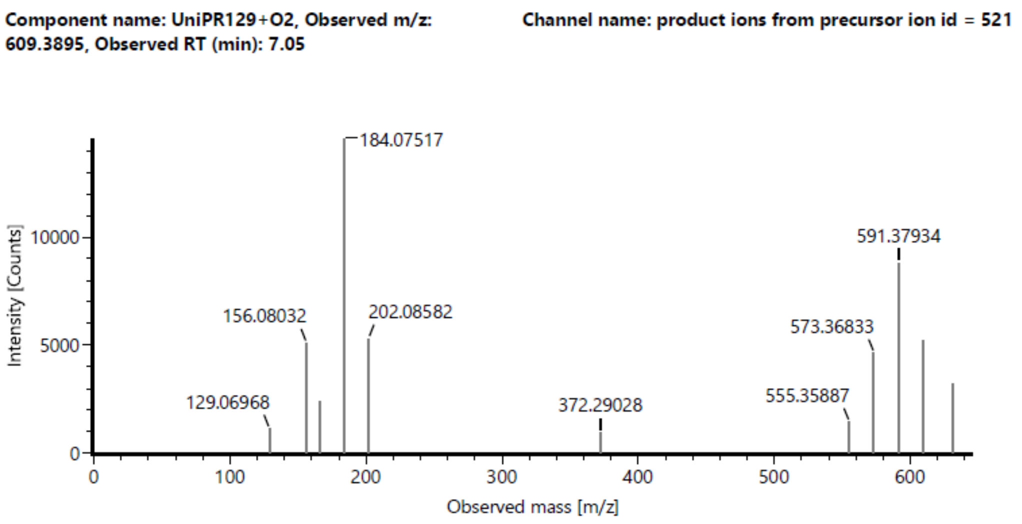

Figure S11. *a-c. Metabolite* ***M10*** *in MLM.*

a. Phase I metabolite **M10** in mouse liver microsomes. Extracted ion chromatogram (XIC) in ESI$^+$ at *m/z* = 609.39 [M+H]$^+$ and RT = 7.16 min.

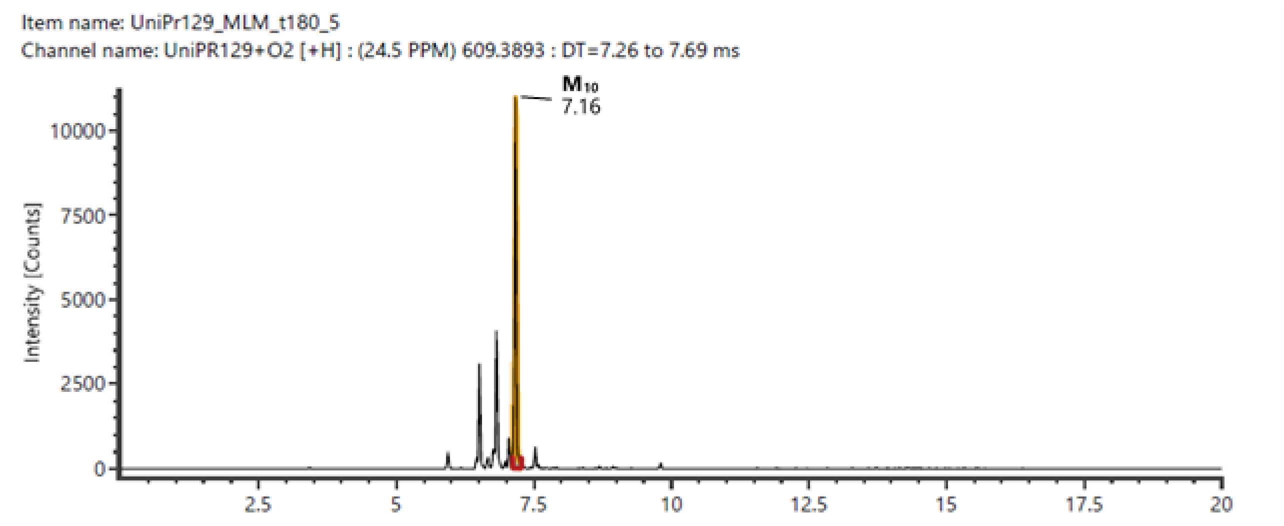

b. Experimental high resolution mass value for metabolite **M10** in ESI⁺.

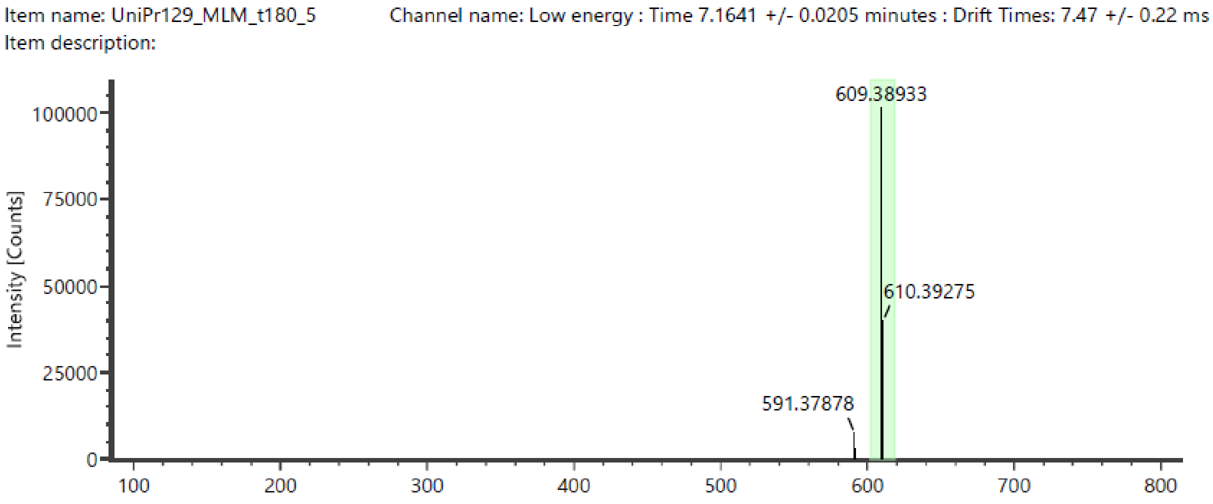

c. High resolution high energy (MS^E) MS spectrum in ESI⁺ of **M10** together with tentative fragmentation pattern. All the ions in the MS/MS spectrum are shared with **M8**; in particular, likewise **M8**, fragment ion at *m/z* = 235.1 could account for a double oxidation on the L-β-homotryptophan portion. However, UNIFI software cannot predict likely sites for oxidation.

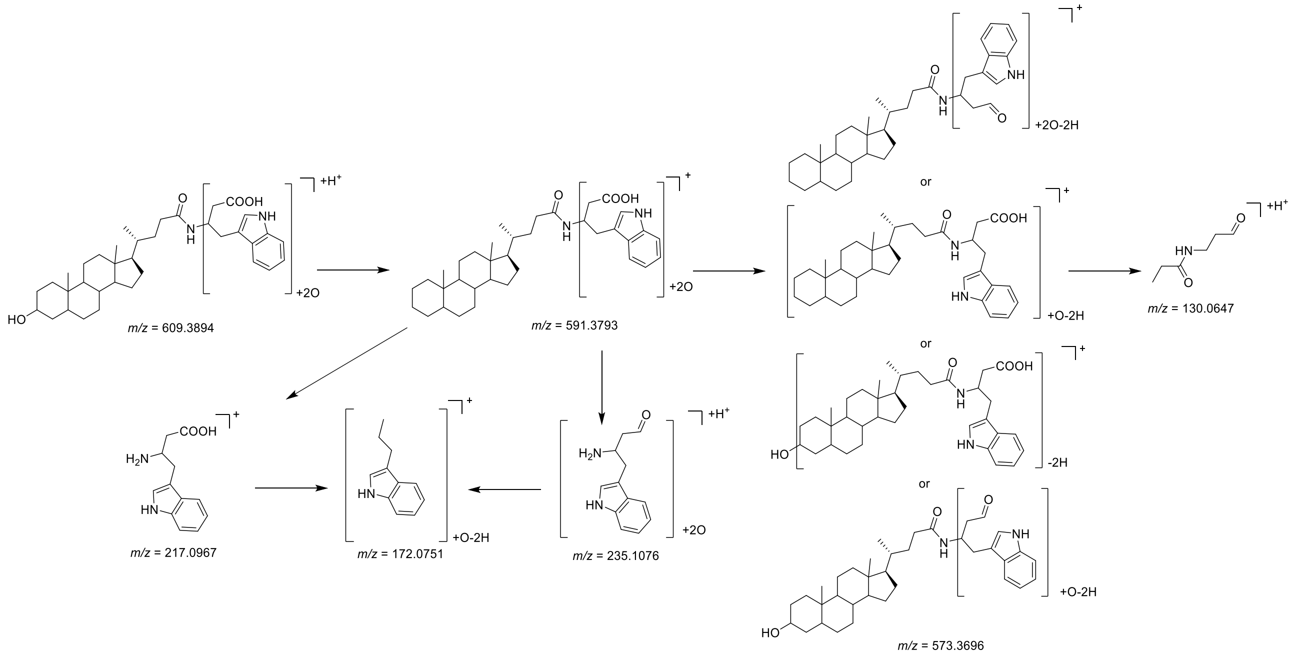

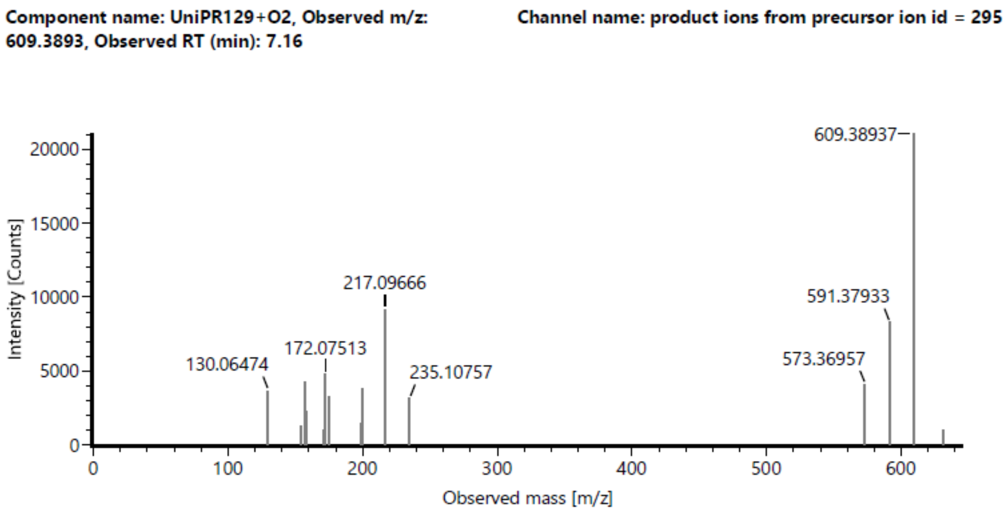

***Figure S12.** a-d. Metabolite **M11** in MLM.*

a. Phase I metabolite **M11** in mouse liver microsomes. Extracted ion chromatogram (XIC) in ESI$^+$ at *m/z* = 591.38 [M+H]$^+$ and RT = 8.06 min.

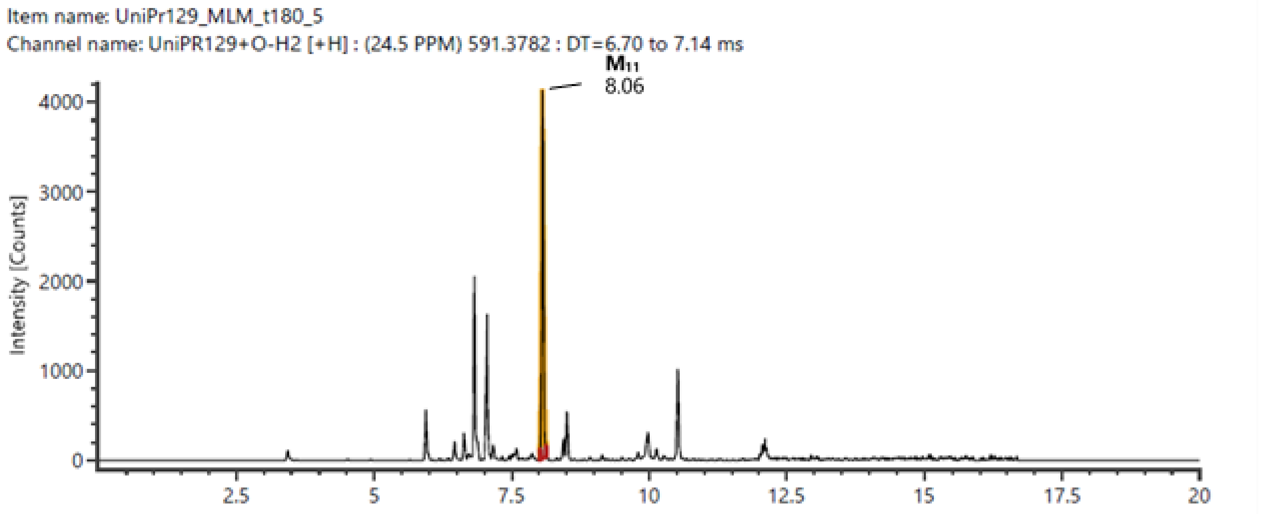

b. Experimental high resolution mass value for metabolite **M11** in ESI+.

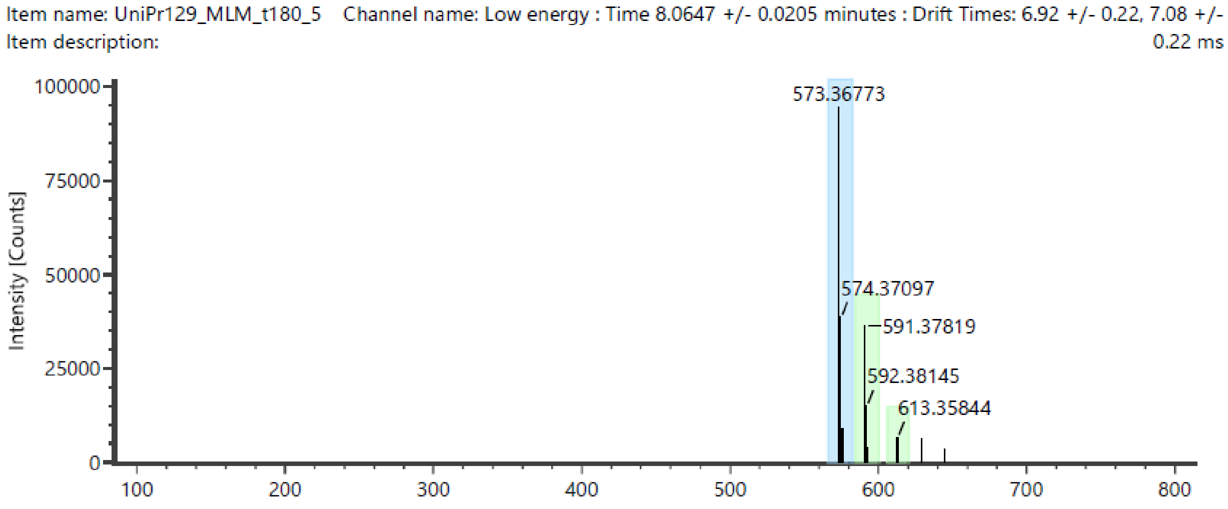

c. High resolution high energy (MS^E) MS spectrum in ESI+ of **M11** together with tentative fragmentation pattern. The three product ions at *m*/*z* = 202.1, 184.1 and 156.1 prompt to exclude the oxidation to occur on L-β-homotryptophan group. Moreover, the low intense fragment ion at *m*/*z* = 337.2 allows to speculate that oxidation involves a C-atom on the A- or B-ring of the steroidal core.

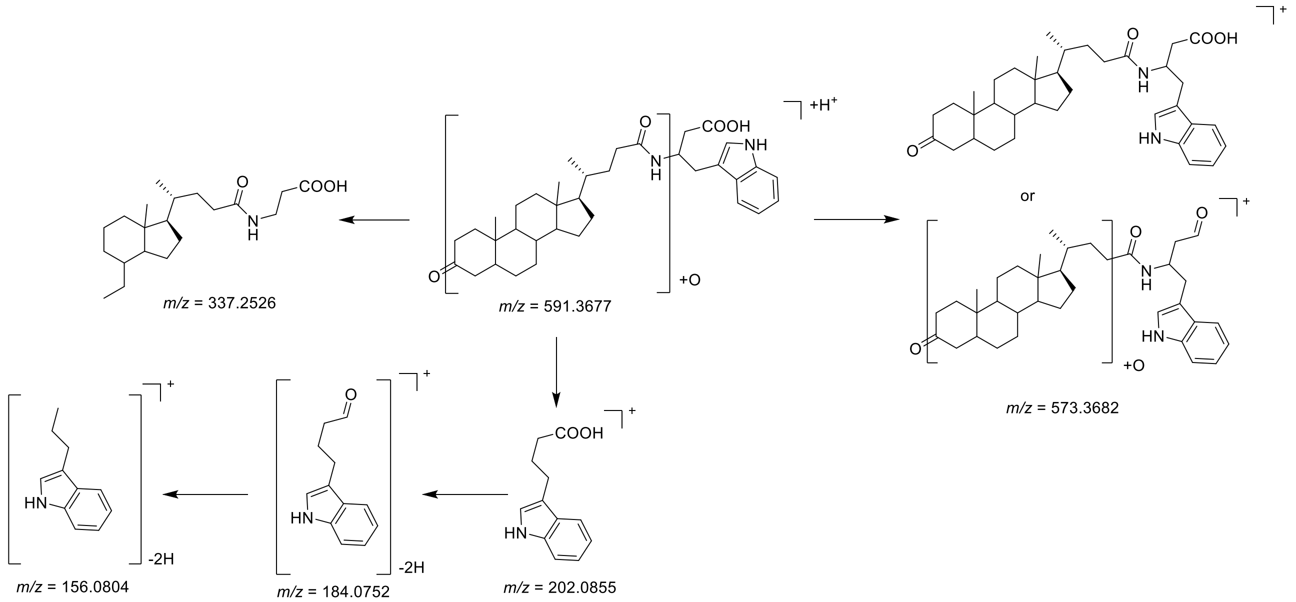

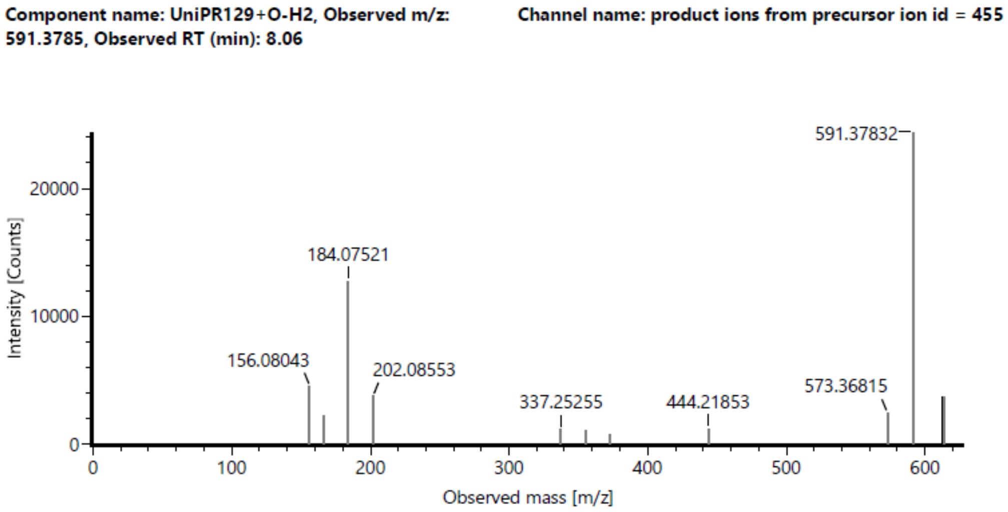

d. Metabolic soft spots on UniPR129 reported with a color-scale: the most probable sites of modification are highlighted in bright green. Based on the MS/MS spectrum of **M11**, UNIFI software returns as the most likely soft spots C1 or C2 of the steroidal core.

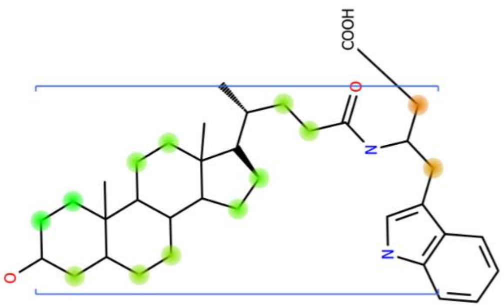

S.7 UniPR129 *In vitro* phase II metabolite profiling

Figure S13. *a-b. Metabolite* ***M12*** *in MLS9 fraction.*

a. Phase II conjugate of UniPR129 with glucuronic acid **M12** in mouse liver microsomes. Upper trace: Extracted ion chromatogram (XIC) in ESI$^-$ at m/z = 575.40 [M-H]$^-$corresponding to parent UniPR129; lower trace: XIC in ESI$^-$ at m/z = 751.42 [M-H]$^-$corresponding to the glucuronide.

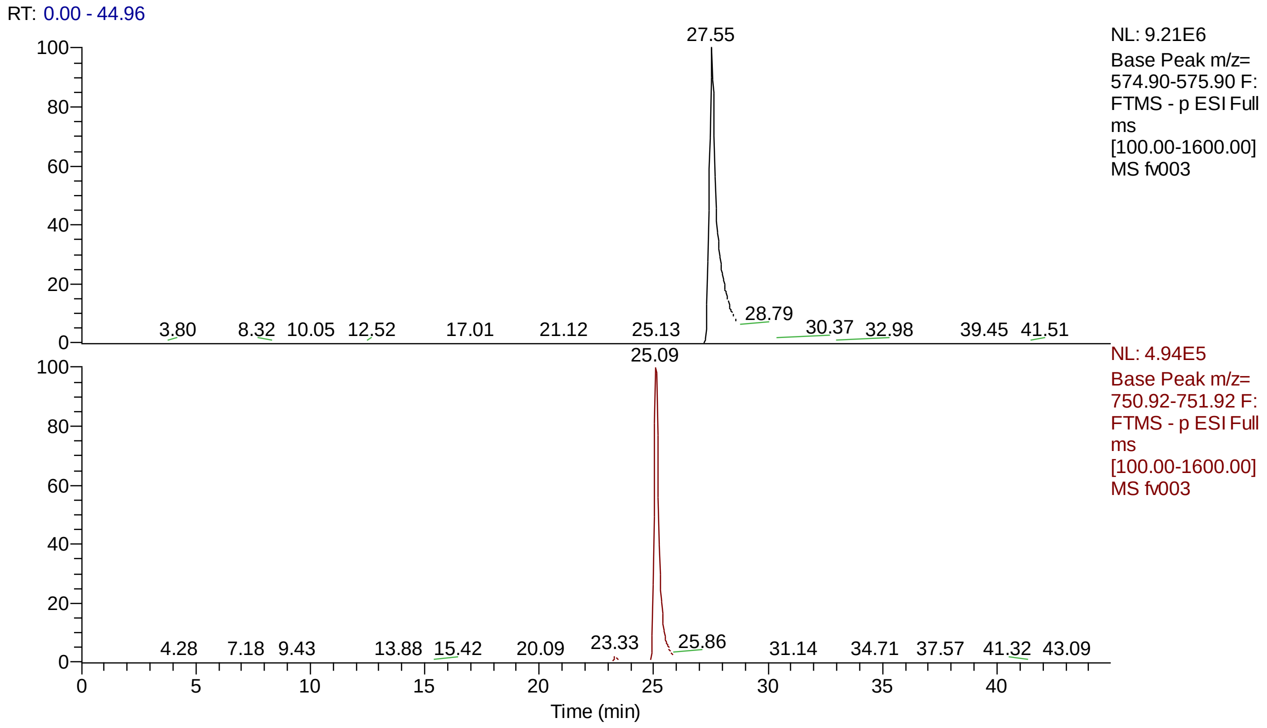

b. Experimental high resolution mass values and isotopic distribution in ESI$^-$ for metabolite **M12** (upper) if compared to calculated value (lower spectrum).

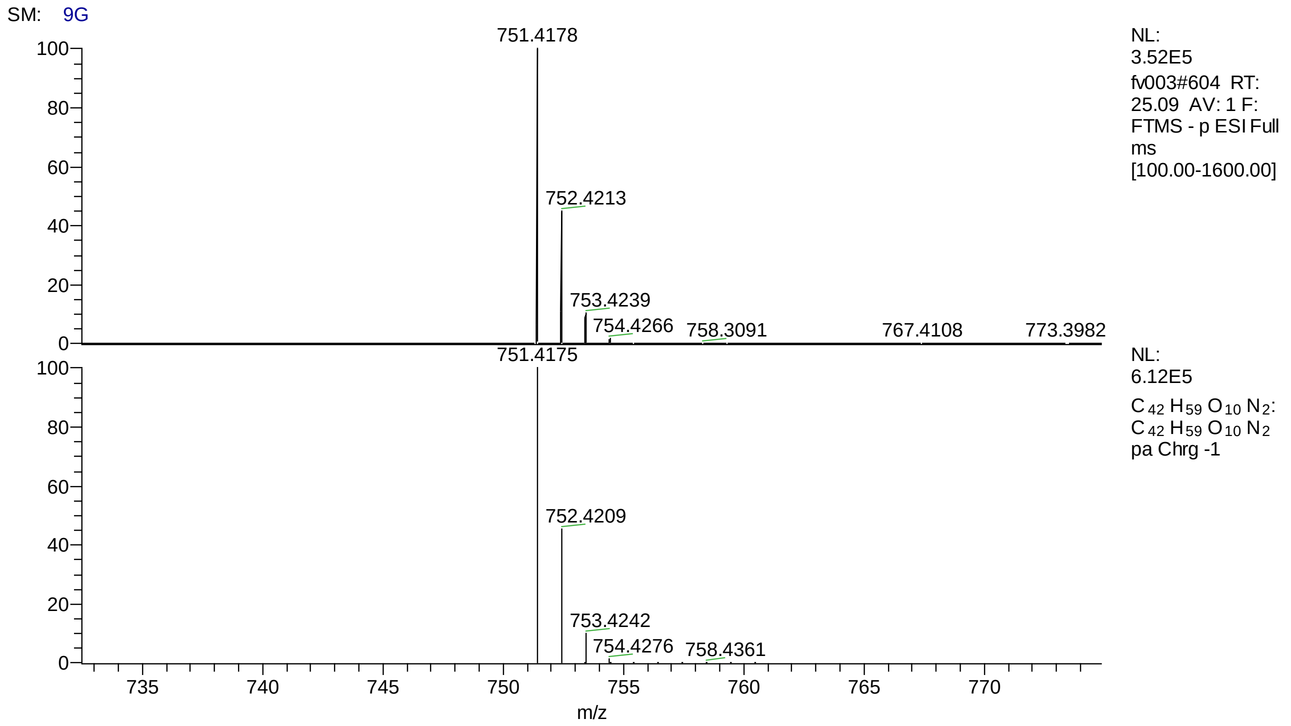

*Figure S14. a-b. Metabolite **M13** in MLS₉ fraction.*

a. Phase II conjugate of UniPR129 with glucuronic acid **M13** in mouse liver microsomes. Upper trace: Extracted ion chromatogram (XIC) in ESI$^-$ at *m/z* = 575.40 [M-H]$^-$ corresponding to parent UniPR129 ; lower trace: XIC in ESI$^-$ at *m/z* = 751.42 [M-H]$^-$ corresponding to the conjugate with glucuronic acid.

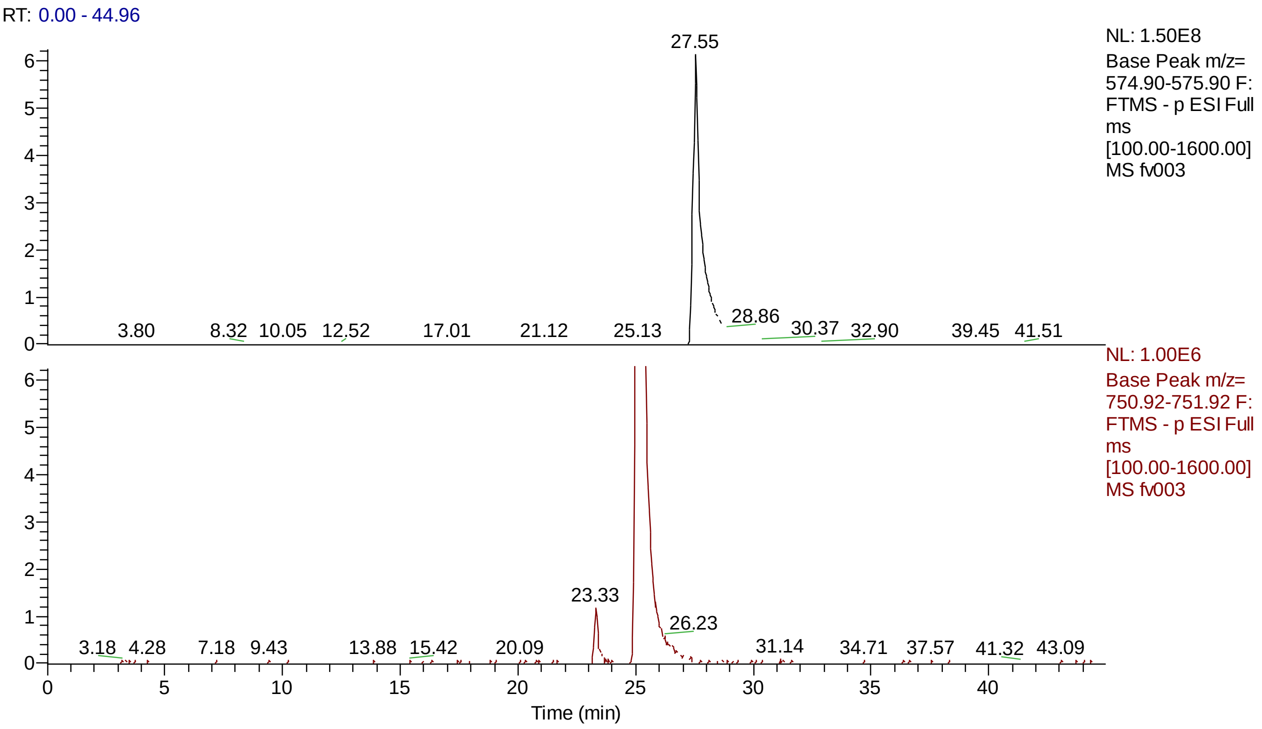

b. Experimental high resolution mass values and isotopic distribution in ESI$^-$ for metabolite **M13** (upper) if compared to calculated value (lower spectrum).

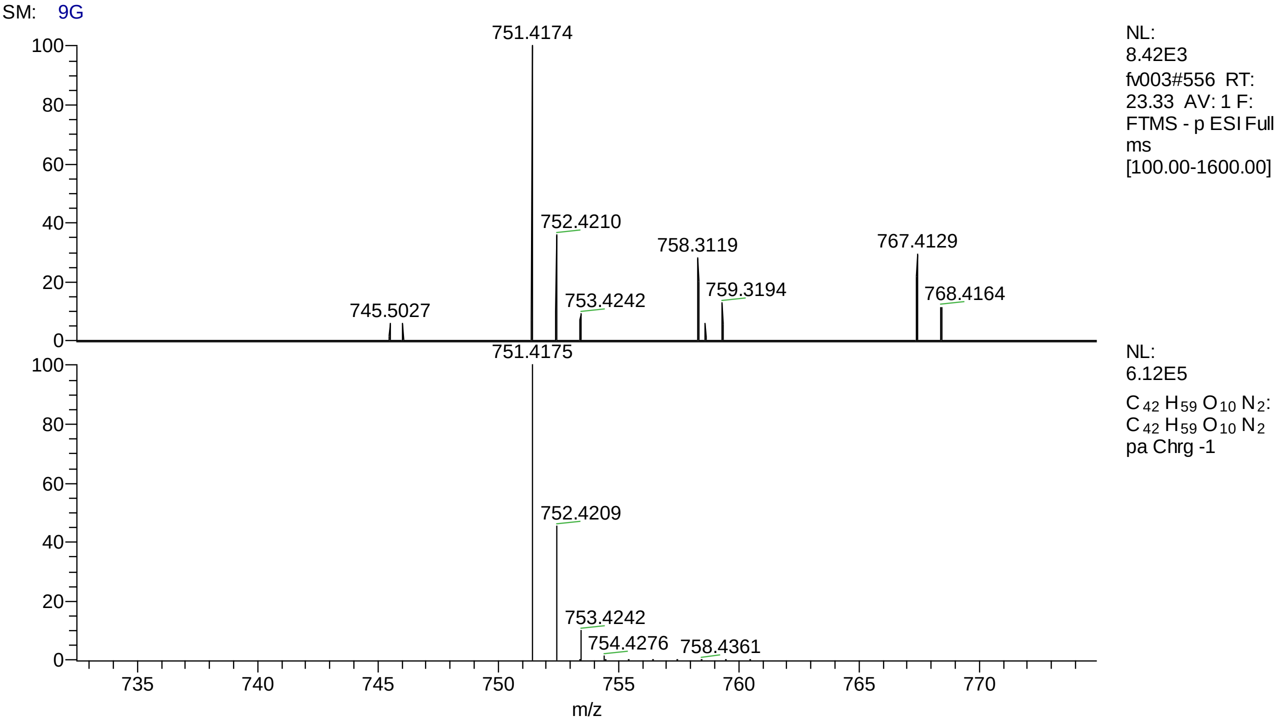

Figure S15. *a-b. Metabolite **M14** in HLS9 fraction.*

a. Phase II conjugate of UniPR129 with active sulphate **M14** in human liver S9 fraction. Upper trace: Extracted ion chromatogram (XIC) in ESI⁻ at *m/z* = 575.40 [M-H]⁻ corresponding to parent UniPR129 ; lower trace: XIC in ESI⁻ at *m/z* = 655.34 [M-H]⁻ corresponding to the conjugate with active sulphate.

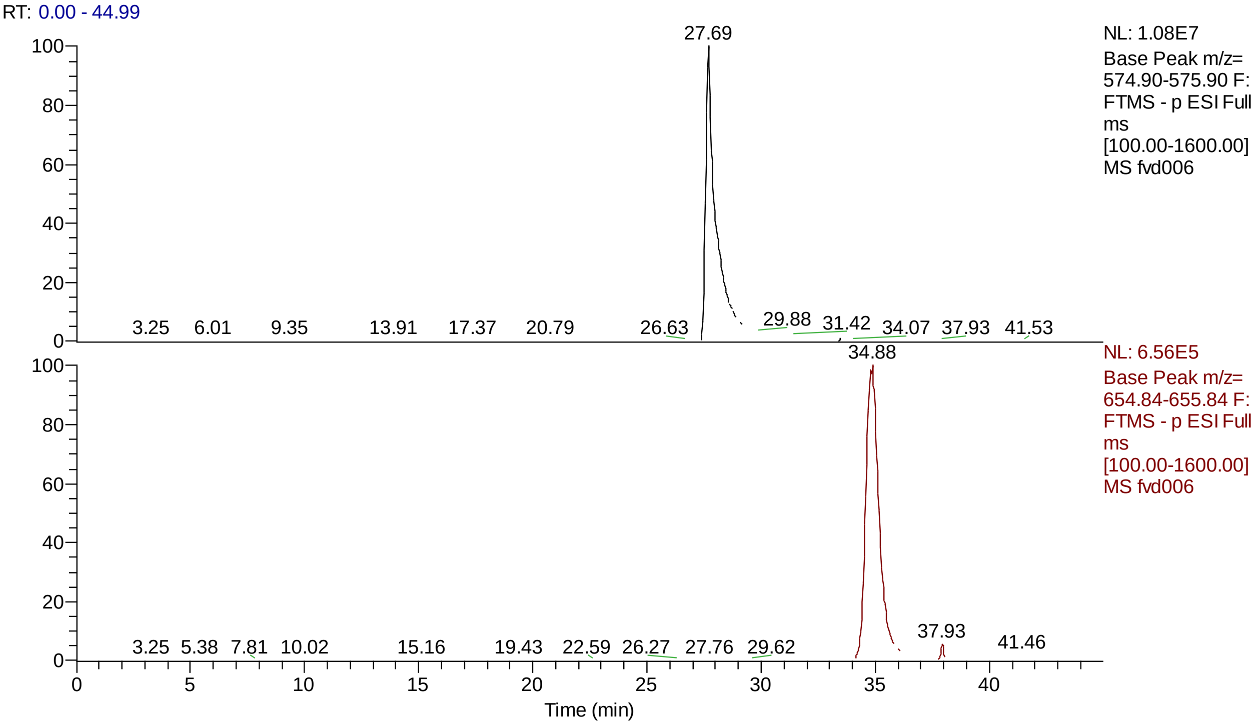

b. Experimental high resolution mass values and isotopic distribution in ESI⁻ for metabolite **M14** (upper) if compared to calculated value (lower spectrum).

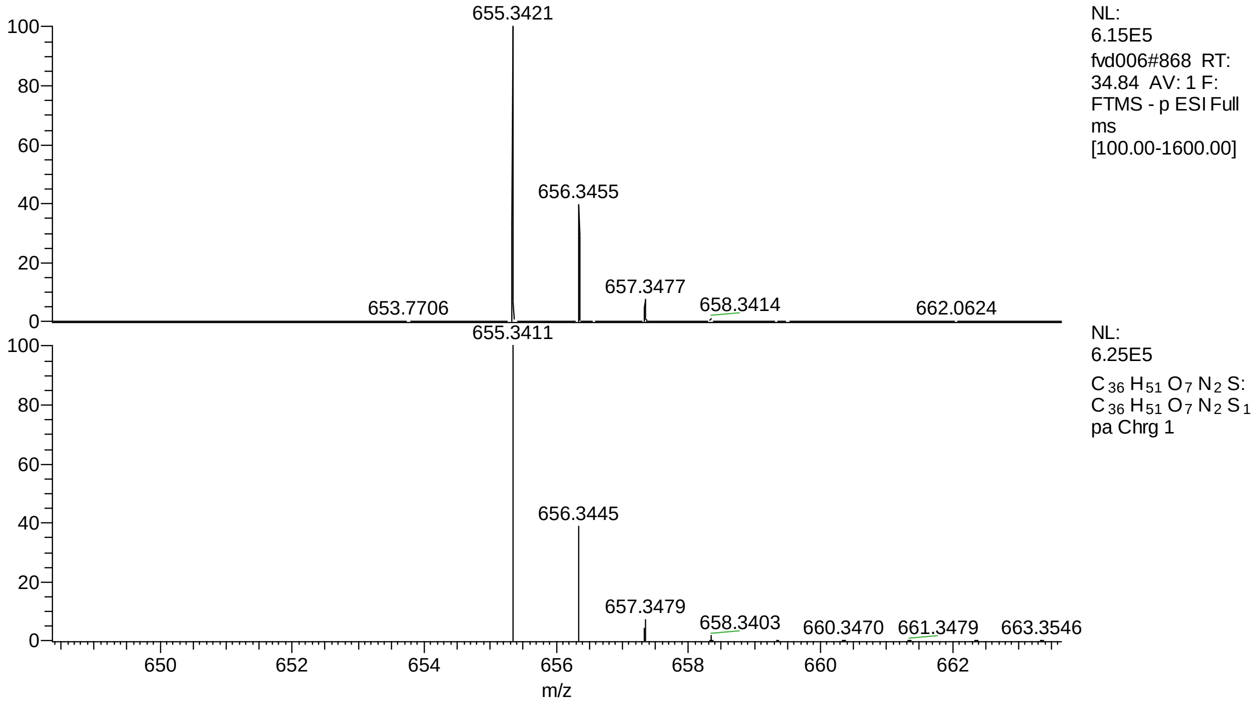

S.8 UniPR500 *In vitro* phase I metabolite profiling

Figure S16. *a-c. Parent compound UniPR500 in MLM.*

a. UniPR500 in mouse liver microsomes. Extracted ion chromatogram (XIC) in ESI$^+$ at *m/z* = 590.39 $[M+H]^+$ and RT = 9.97 min.

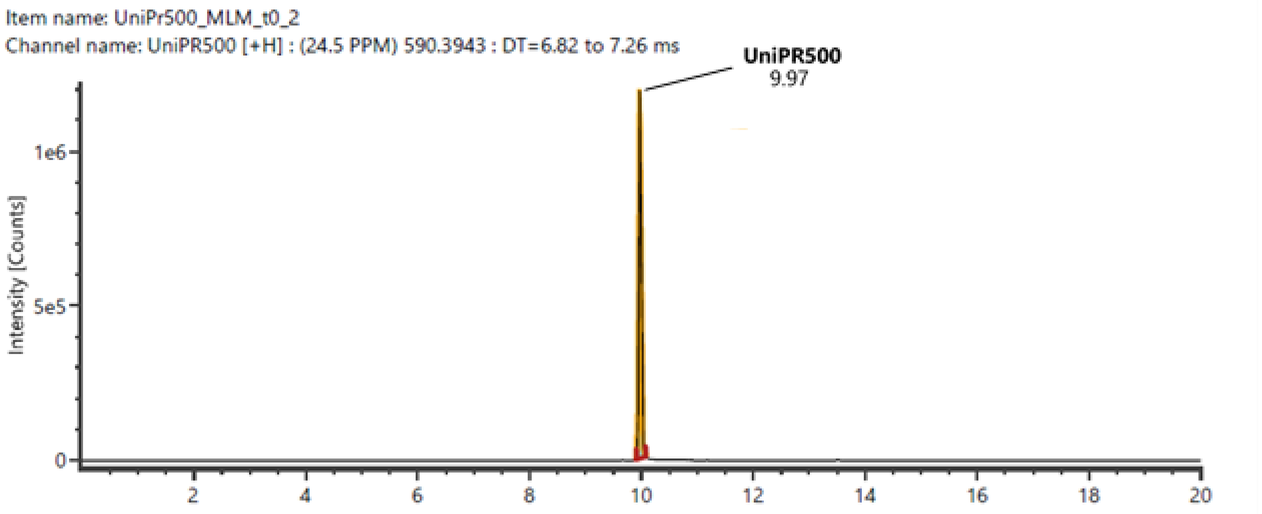

b. Experimental high resolution mass value in ESI$^+$ for UniPR500.

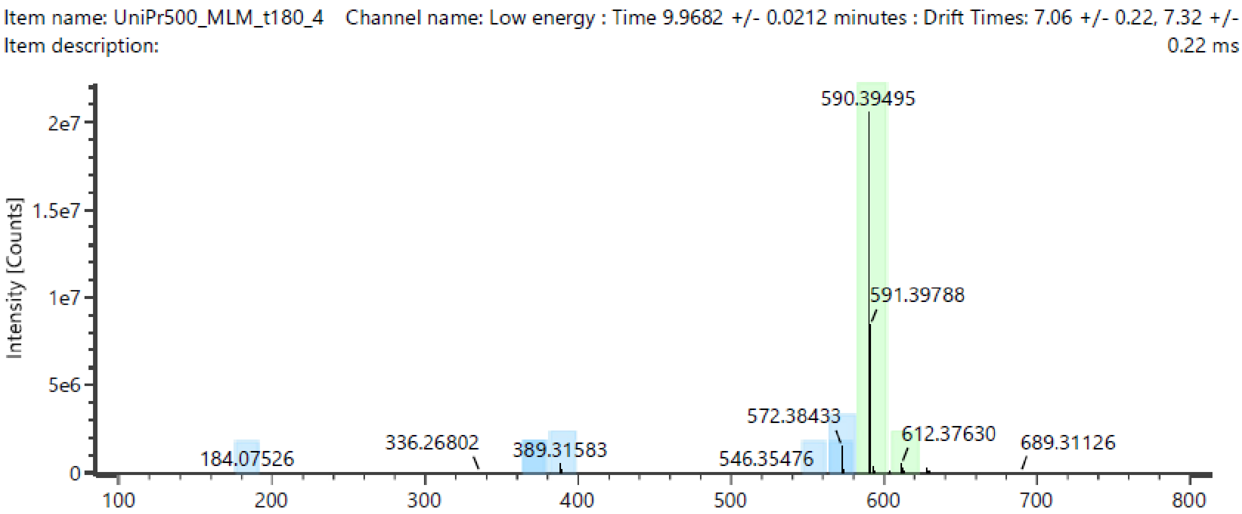

c. High resolution high energy (MS^E) MS spectrum in ESI^+ of UniPR500 together with tentative fragmentation pattern. The most intense signal, other than that of molecular ion, is represented by *m/z* = 572.4 that accounts for loss of water (-18). A peculiar ion fragment at *m/z* = 389.3 is compatible with the loss of the 4-(indol-3-yl)-butanoyl group, liberated as the *m/z* = 202.1 ion. The ion triad *m/z* = 202.1, 184.1 and 156.1, already reported for UniPR129, is found in the MS/MS spectrum of several metabolites of UniPR500.

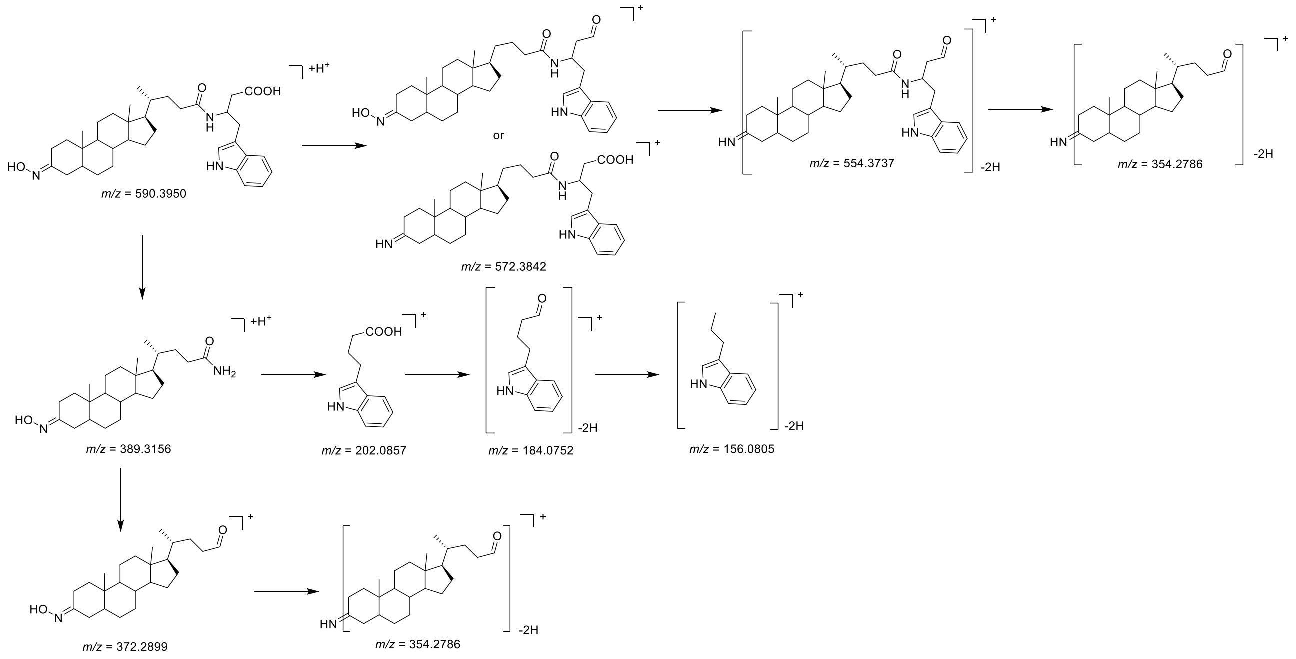

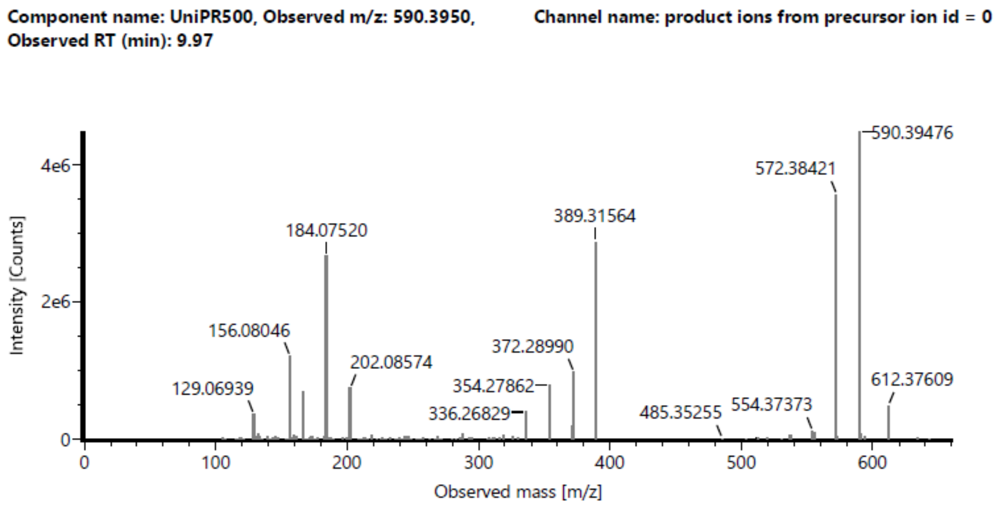

Figure S17. *a-d. Metabolite* ***M1*** *in MLM.*

a. Phase I Metabolite **M1** derived from UniPR500 in mouse liver microsomes. Extracted ion chromatogram (XIC) in ESI$^+$ at m/z = 575.38 [M+H]$^+$ and RT = 10.64 min.

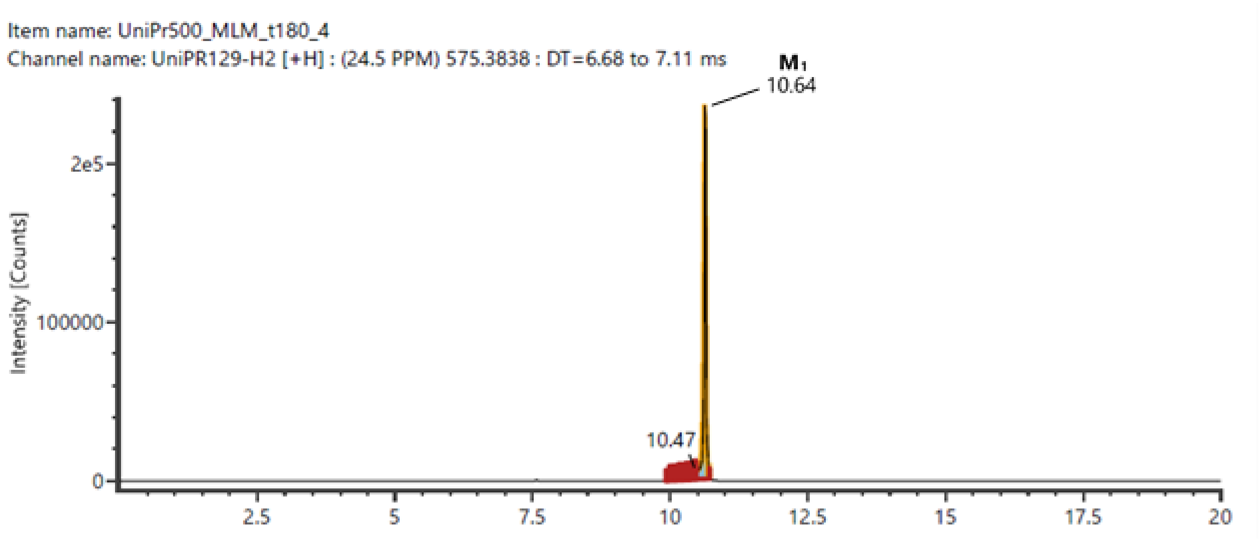

b. Experimental high resolution mass value in ESI$^+$ for metabolite **M1**.

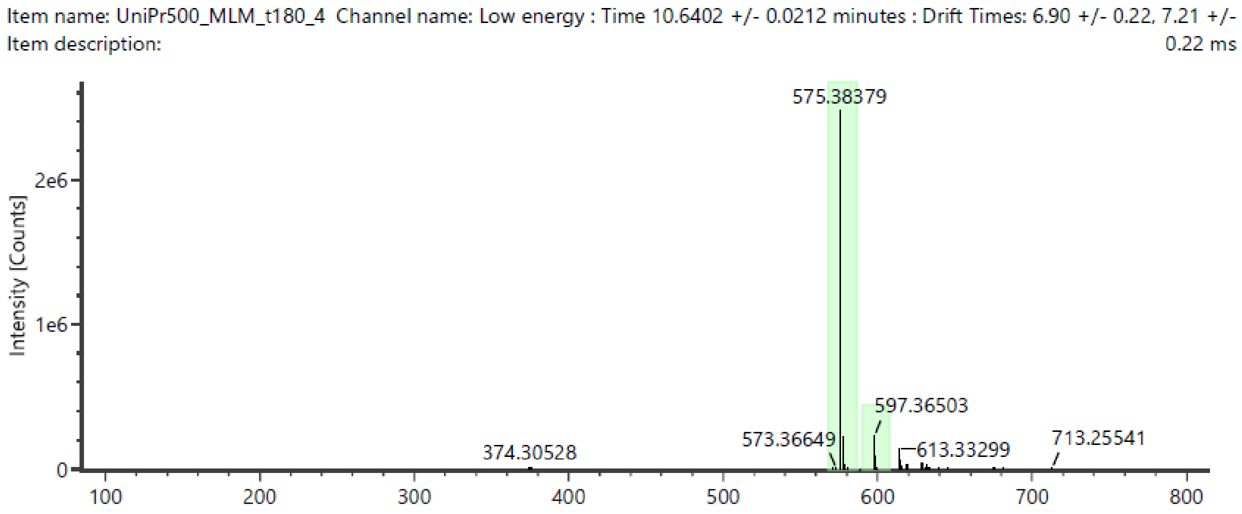

c. High resolution high energy (MS^E) MS spectrum in ESI$^+$ of 3-keto metabolite **M1** together with tentative fragmentation pattern. Considering the exact mass, the RT and the MS/MS spectrum, this metabolite reasonably corresponds to the M1 reported for UniPR129.

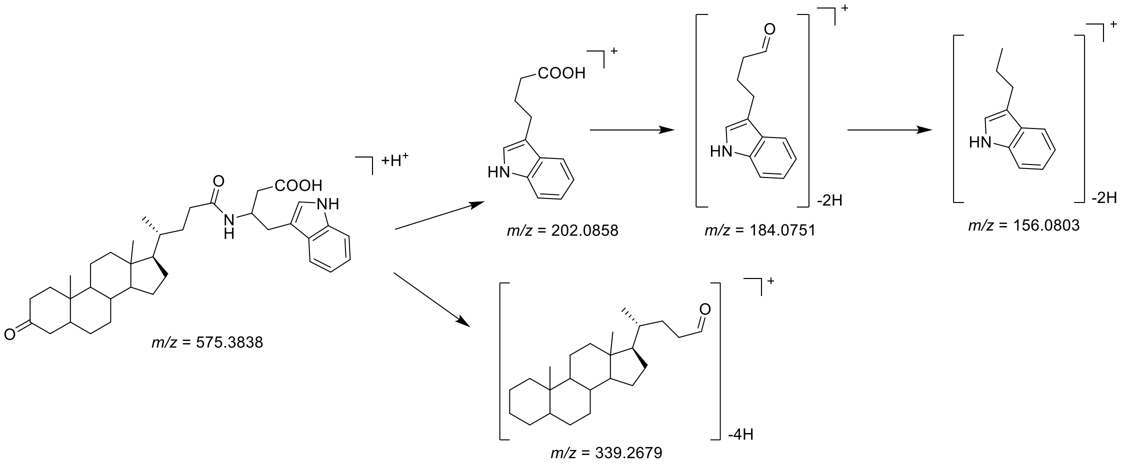

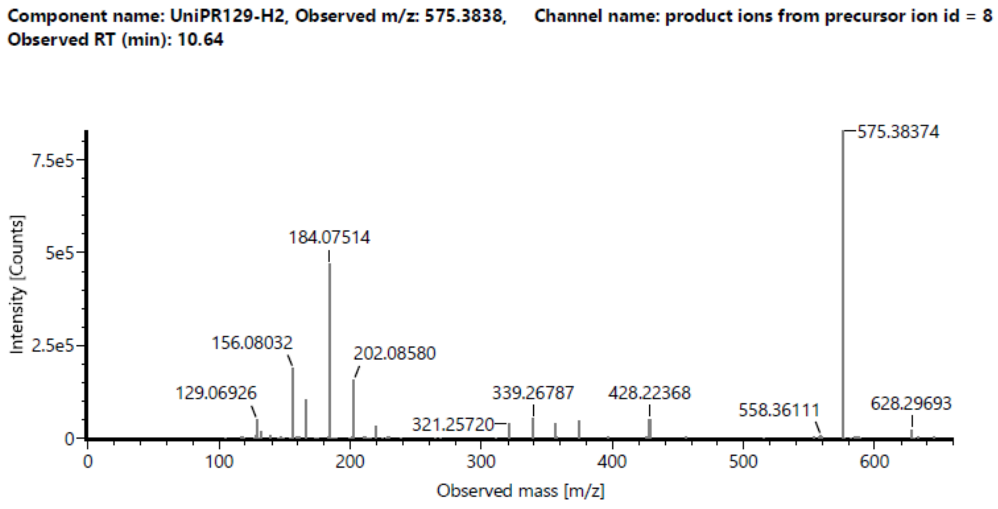

Figure S18. *a-c. UniPR129 derived from UniPR500 in MLM.*

a. UniPR129 derived from UniPR500 in mouse liver microsomal incubations. Extracted ion chromatogram (XIC) in ESI^+ at *m/z* = 577.40 $[M+H]^+$ and RT = 10.52 min.

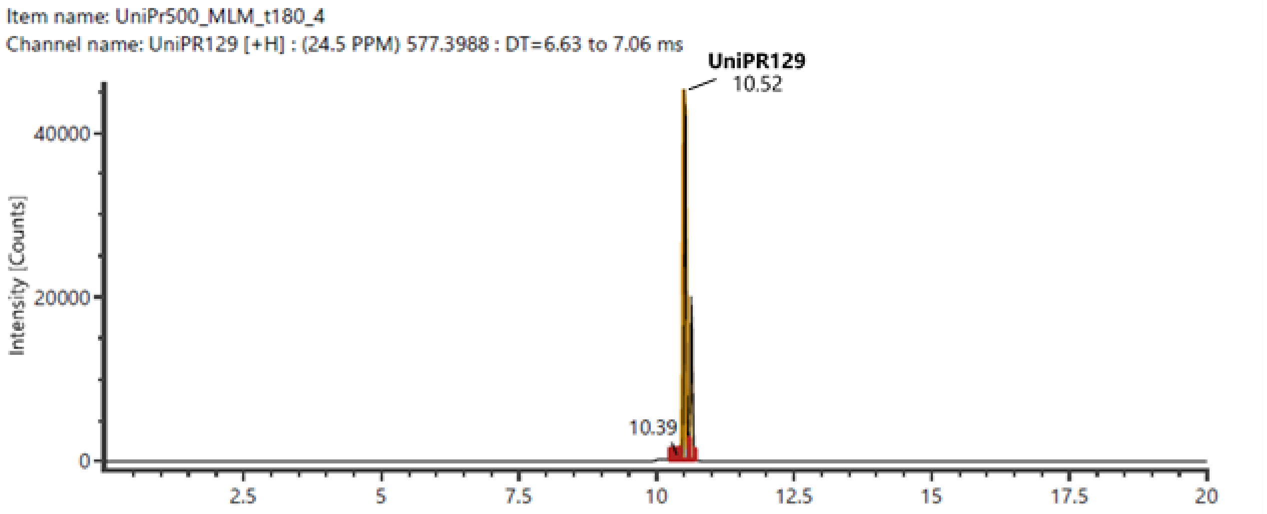

b. Experimental high resolution mass value in ESI^+ for UniPR129.

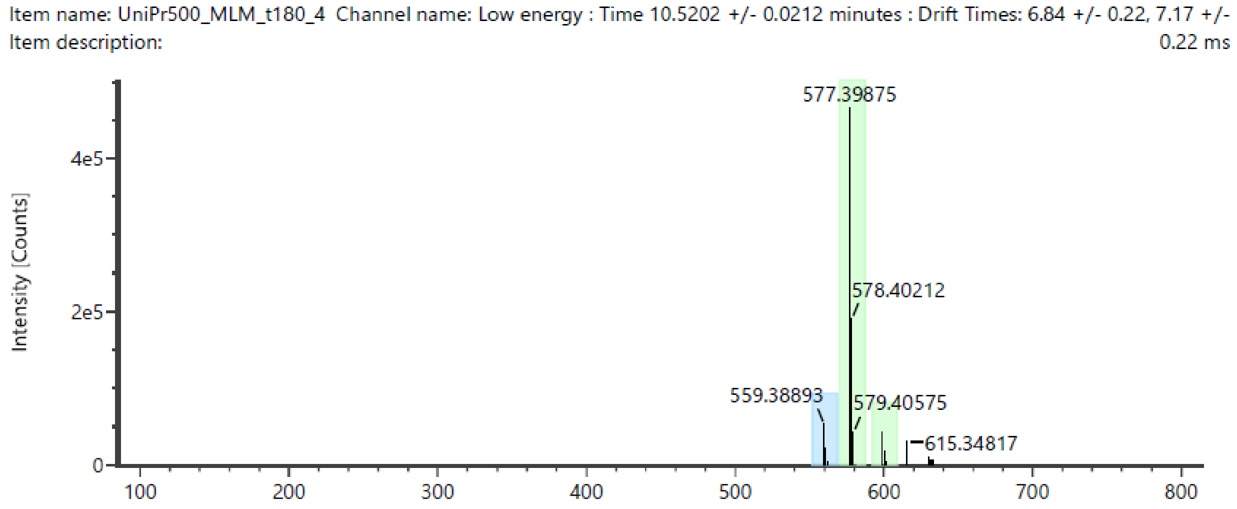

c. High resolution high energy (MS^E) MS spectrum in ESI$^+$ of UniPR129 together with tentative fragmentation pattern.

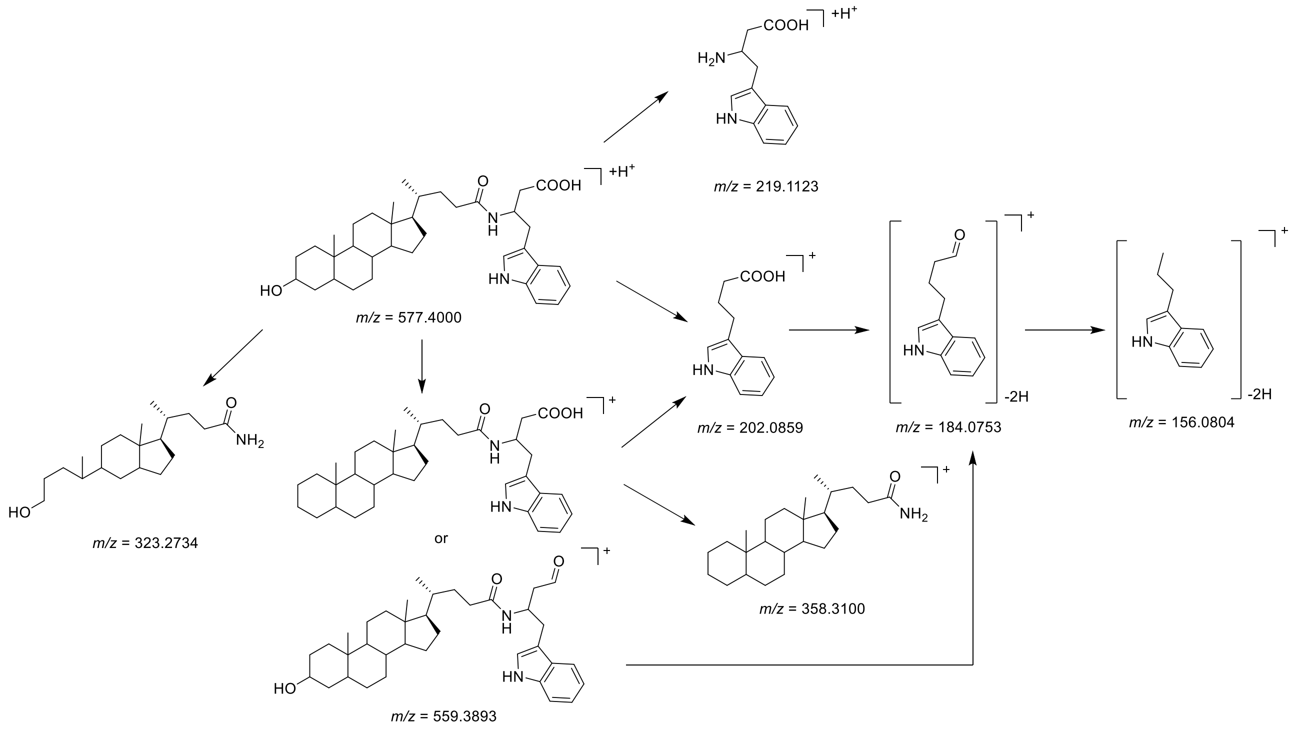

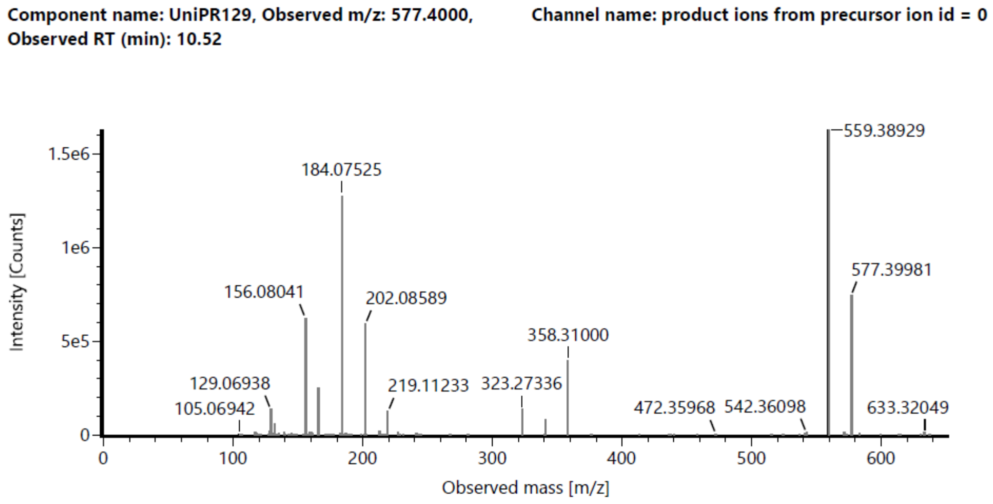

Figure S19. *a-d. Metabolite **M2** in MLM.*

a. Phase I Metabolite **M2** derived from UniPR500 in mouse liver microsomes. Extracted ion chromatogram (XIC) in ESI$^+$ at m/z = 606.39 [M+H]$^+$ and RT = 7.50 min.

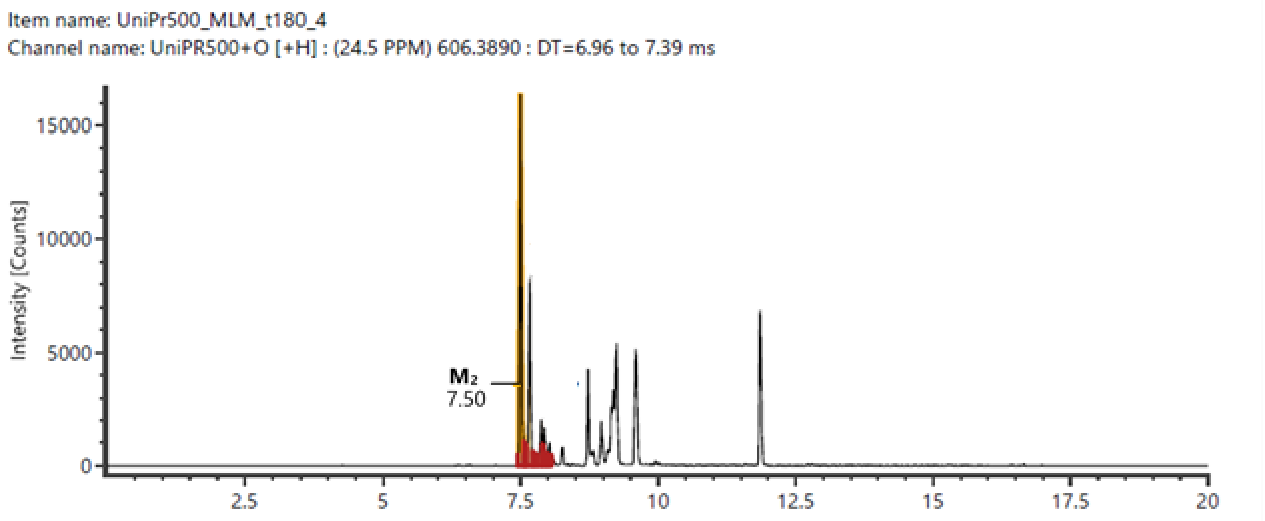

b. Experimental high resolution mass value in ESI$^+$ for **M2**.

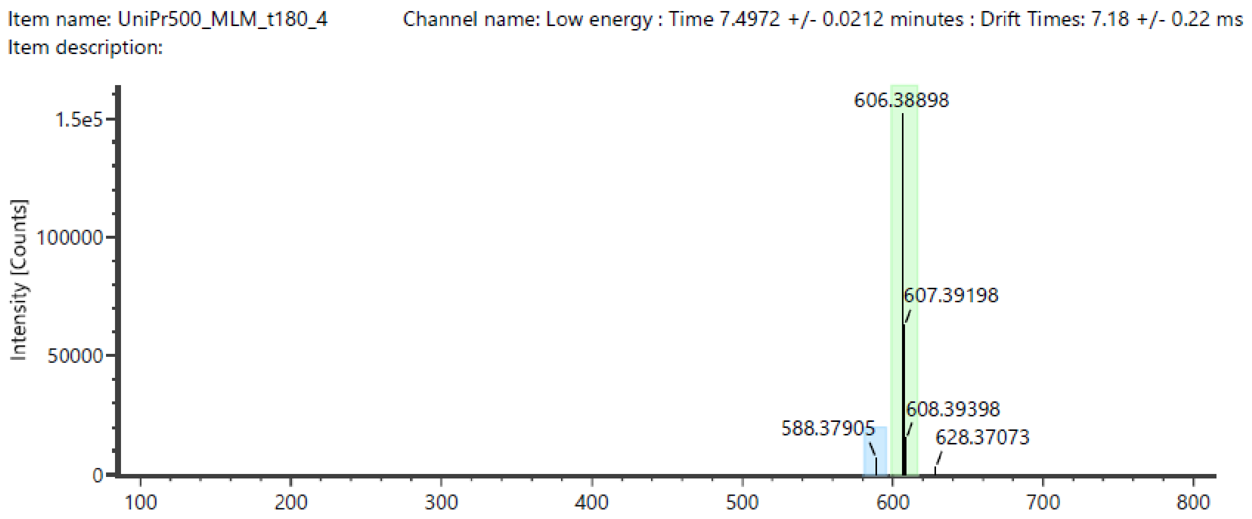

c. High resolution high energy (MS^E) MS spectrum in ESI$^+$ of **M2** together with tentative fragmentation pattern. The most intense fragment ion in the MS/MS spectrum is at *m/z* = 588.4, which could be due to a loss of water (-18); peak at *m/z* = 528.36 is compatible with the loss of the methyl-carboxy group and a water molecule (-60) with respect to *m/z* = 588.4. The further loss of the 4-(indol-3-yl)-butanoyl group could account for the generation of the peculiar fragment at *m/z* = 387.3, which, in turn, liberates the fragment ion at *m/z* = 345.2, probably by losing a hydroxy-ethyl group from the A-ring of the steroidal core. These two product ions suggest oxidation to occur on C-1 or C-2 of the steroid.

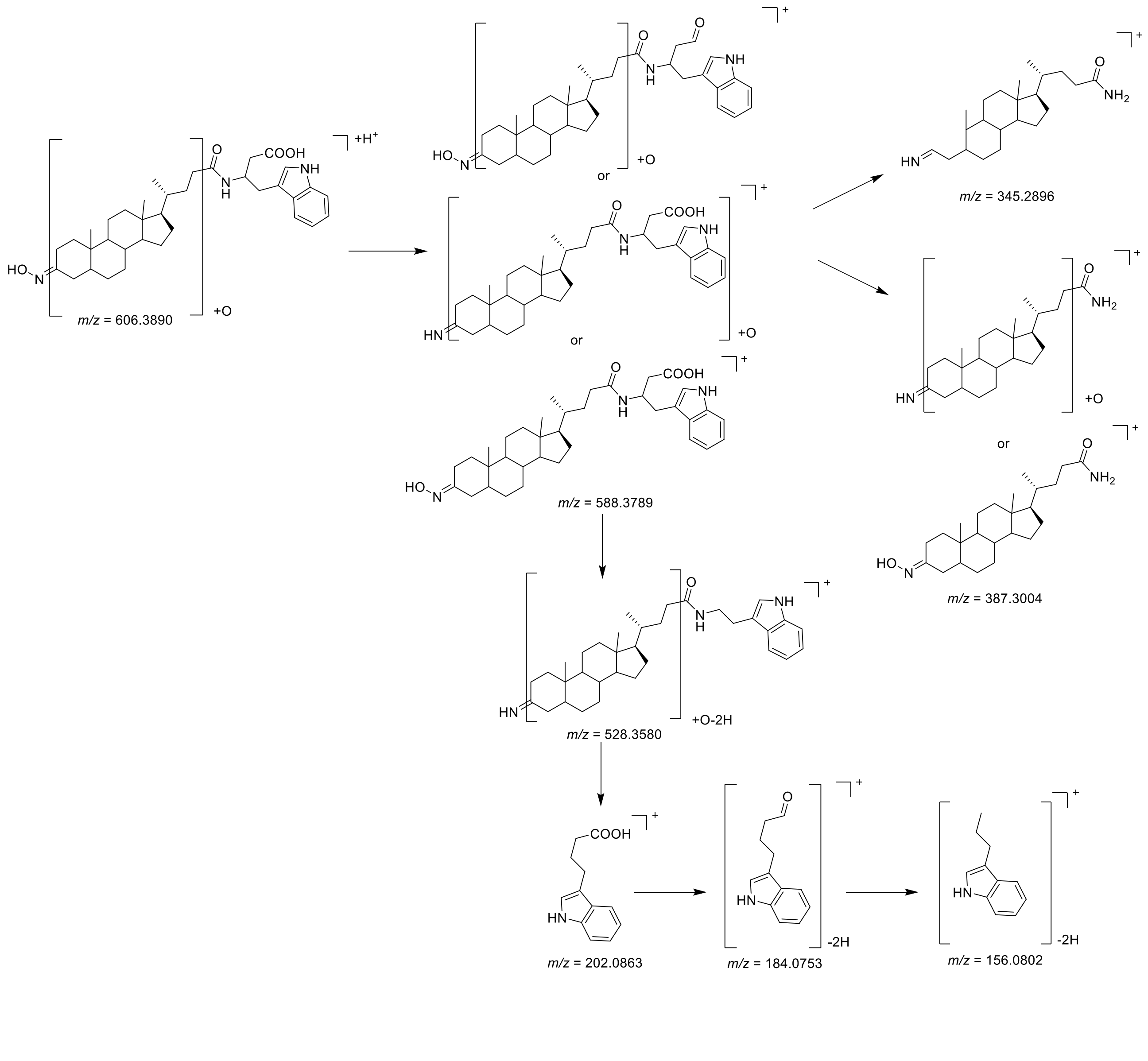

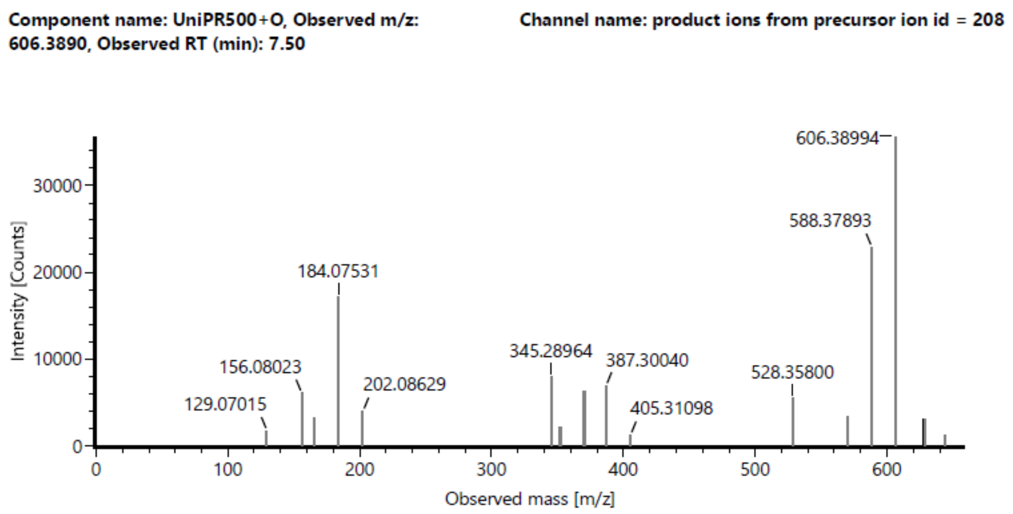

d. Metabolic soft spots on UniPR500 reported with a color-scale: the most probable sites of modification are highlighted in bright green. Based on the MS/MS spectrum of **M2**, UNIFI software returns as the most likely soft spots C-1-23 of the steroidal core.

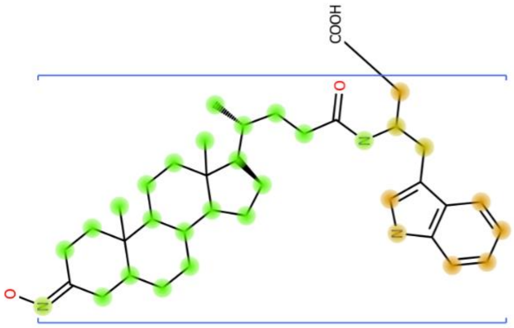

Figure S20. *a-d. Metabolite* ***M3*** *in MLM.*

a. Phase I Metabolite **M3** derived from UniPR500 in mouse liver microsomes. Extracted ion chromatogram (XIC) in ESI$^+$ at m/z = 606.39 [M+H]$^+$ and RT = 7.66 min.

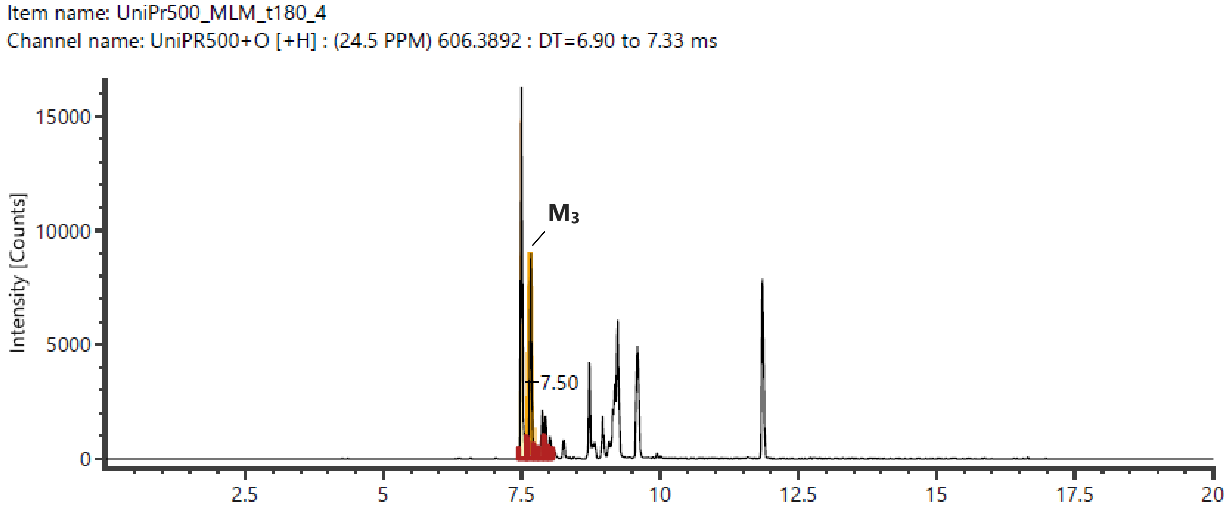

b. Experimental high resolution mass value in ESI$^+$ for **M3.**

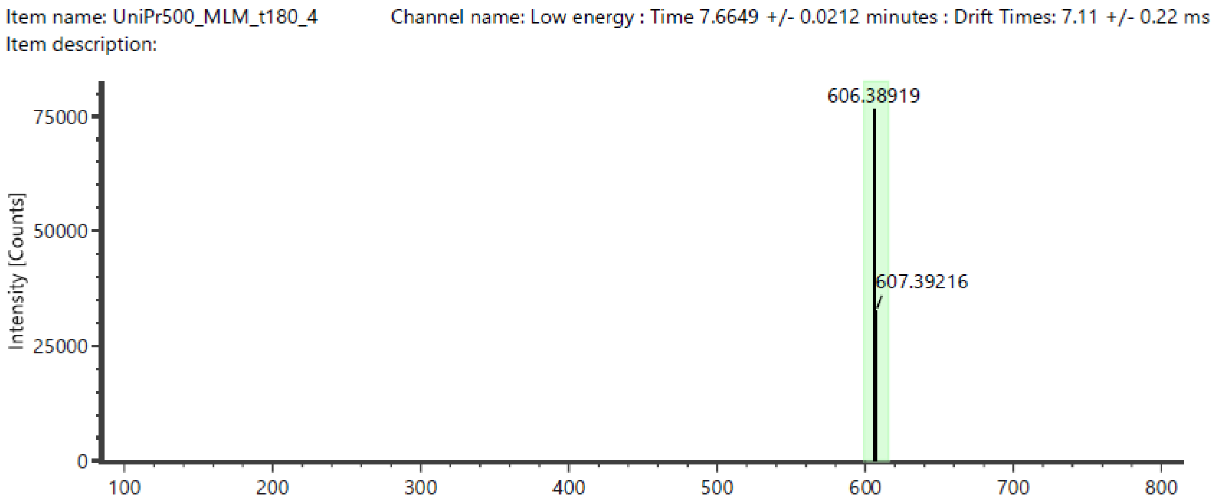

c. High resolution high energy (MS^E) MS spectrum in ESI$^+$ of **M3** together with tentative fragmentation pattern. Fragment ions at *m/z* = 588.4 and 570.4 have already been reported for **M3**. A characteristic peak is found in the MS/MS spectrum at *m/z* = 405.3, which could account for the loss of the 4-(indol-3-yl)butanoxy fragment from 570.4, and suggests oxidation to occur on the steroidal moiety. Another peculiar ion peak is at *m/z* = 352.3, which is compatible with the loss of the amino group and two water molecules from 405.3.

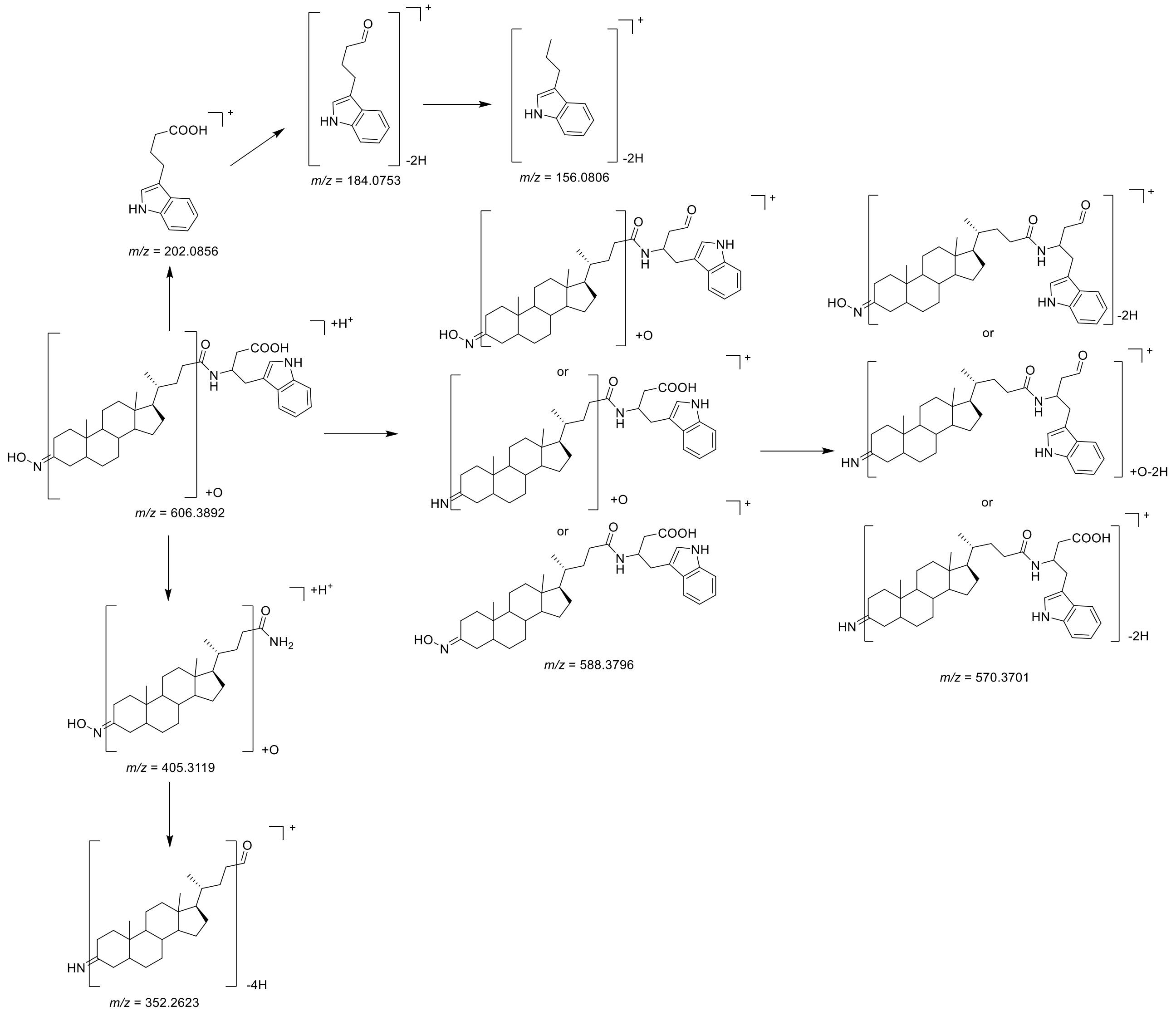

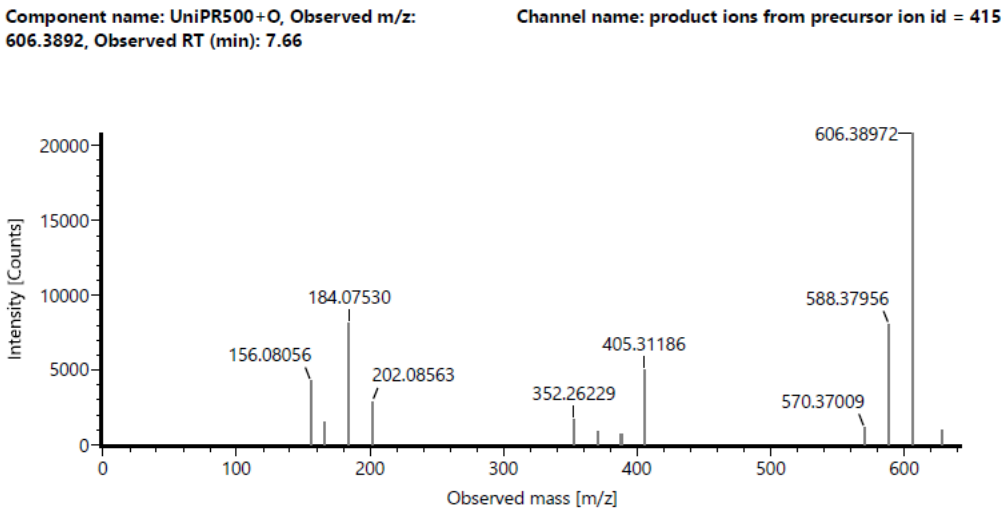

d. Metabolic soft spots on UniPR500 reported with a color-scale: the most probable sites of modification are highlighted in bright green. Based on the MS/MS spectrum of **M3**, UNIFI software returns as the most likely soft spots C-1-23 of the steroidal core, likewise **M2**.

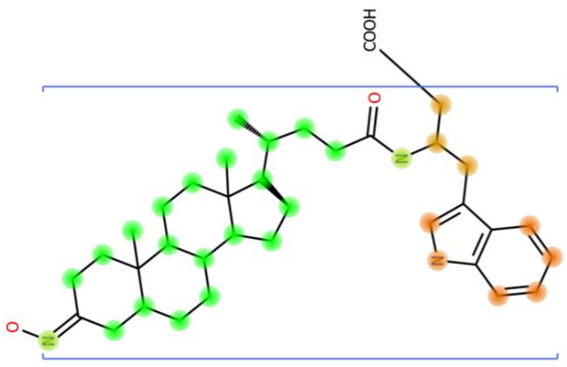

__Figure S21.__ a-d. Metabolite __M4__ in MLM.

a. Phase I Metabolite **M4** derived from UniPR500 in mouse liver microsomes. Extracted ion chromatogram (XIC) in ESI$^+$ at m/z = 606.39 [M+H]$^+$ and RT = 7.93 min.

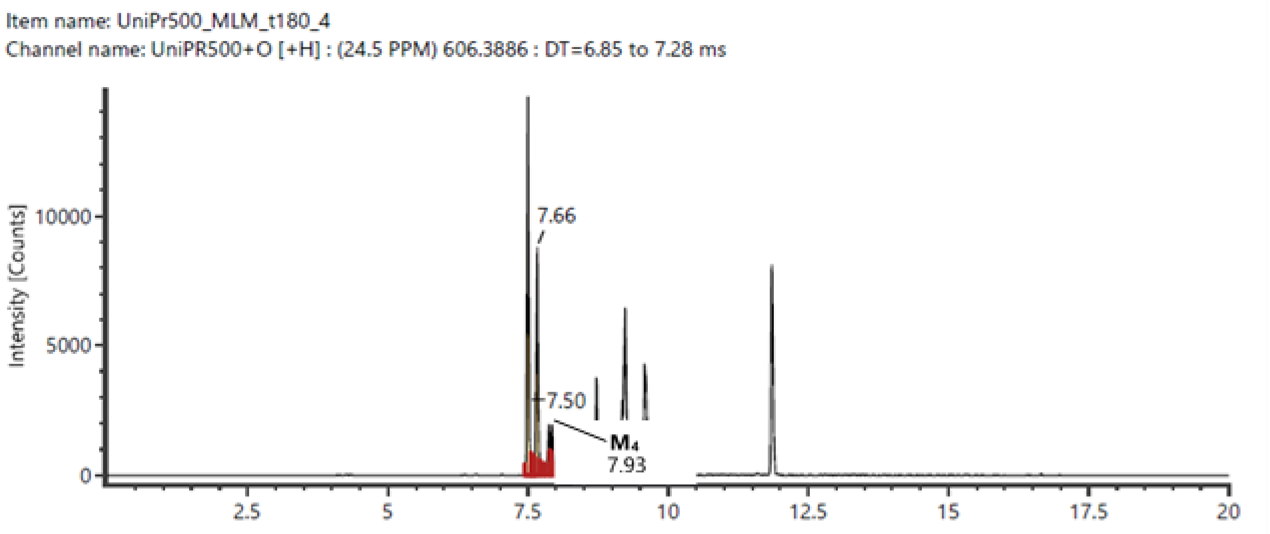

b. Experimental high resolution mass value in ESI$^+$ for **M4**.

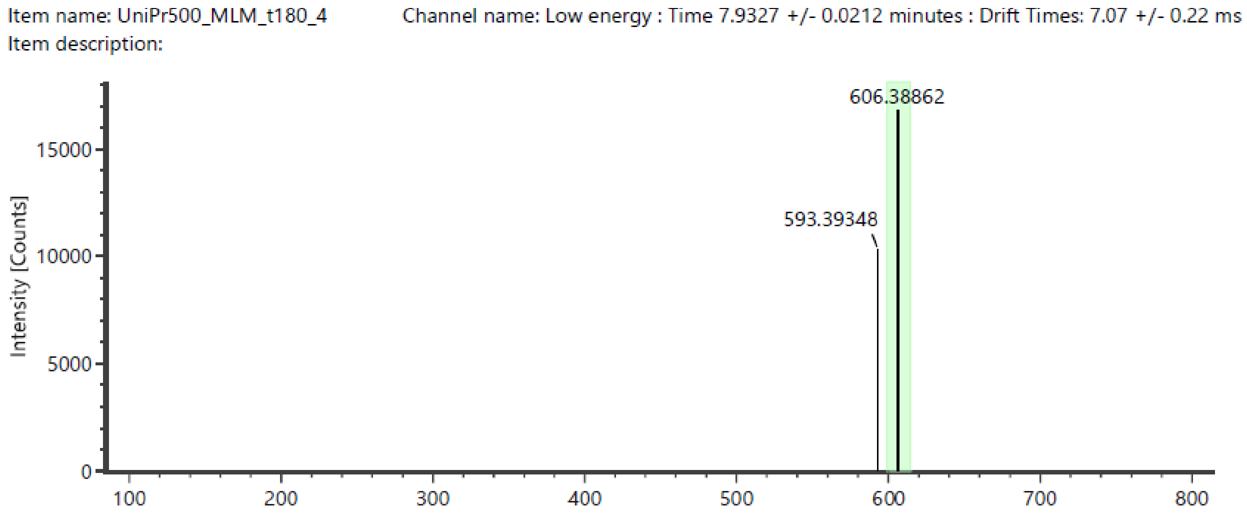

c. High resolution high energy (MS^E) MS spectrum in ESI^+ of **M4** together with tentative fragmentation pattern. The characteristic ion of the MS/MS spectrum is at *m/z* = 557.4, possibly deriving from *m/z* = 570.5 after loss of the oxime moiety (-31) and a water molecule (-18).

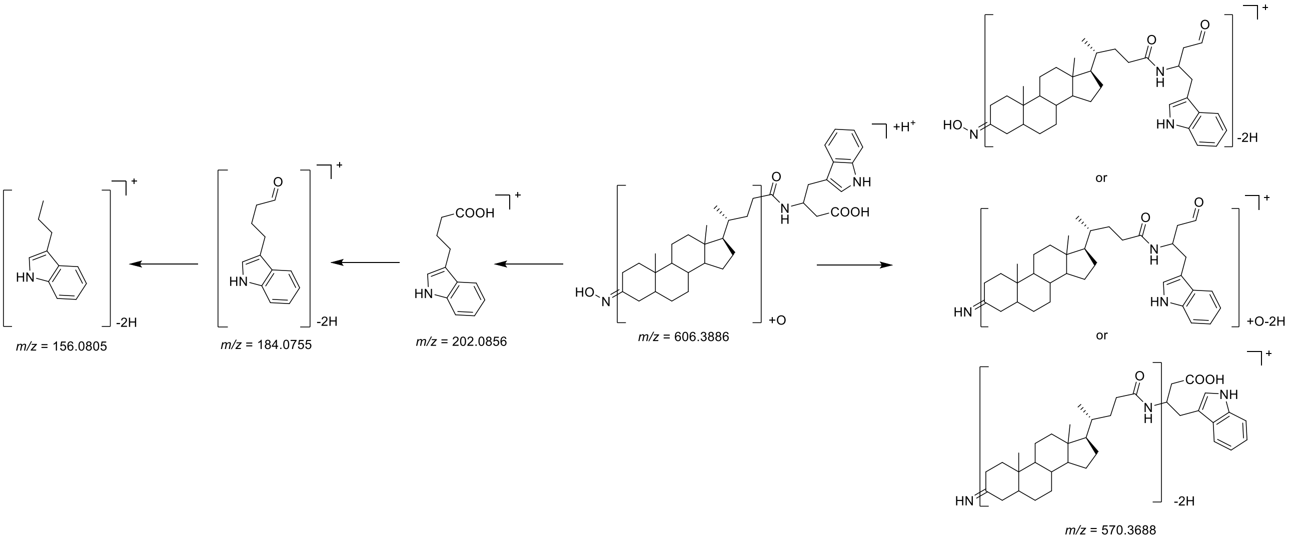

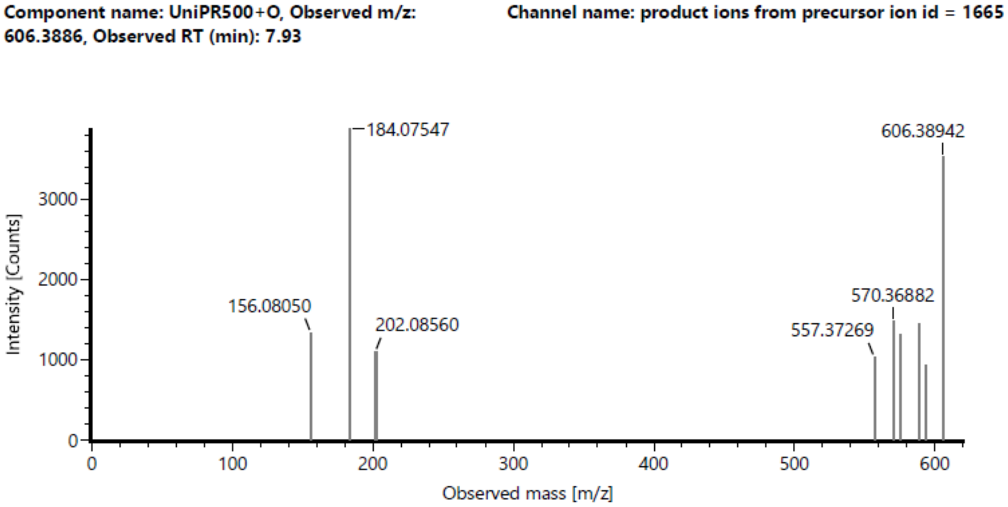

d. Metabolic soft spots on UniPR500 reported with a color-scale: the most probable sites of modification are highlighted in bright green. Based on the MS/MS spectrum of **M4**, UNIFI software returns as the most likely soft spots C-1, C-2 and C-4-23 of the steroidal core.

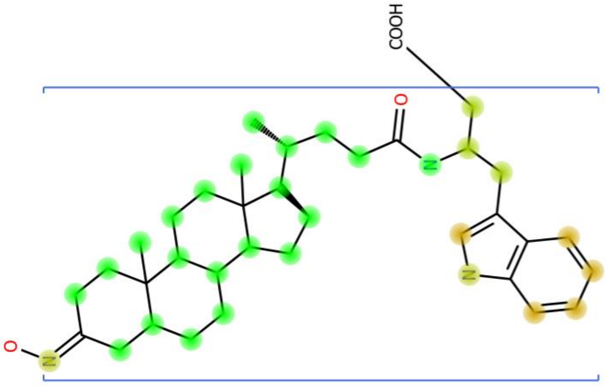

__Figure S22.__ a-d. Metabolite __M5__ in MLM.

a. Phase I Metabolite **M5** derived from UniPR500 in mouse liver microsomes. Extracted ion chromatogram (XIC) in ESI$^+$ at m/z = 606.39 [M+H]$^+$ and RT = 8.72 min.

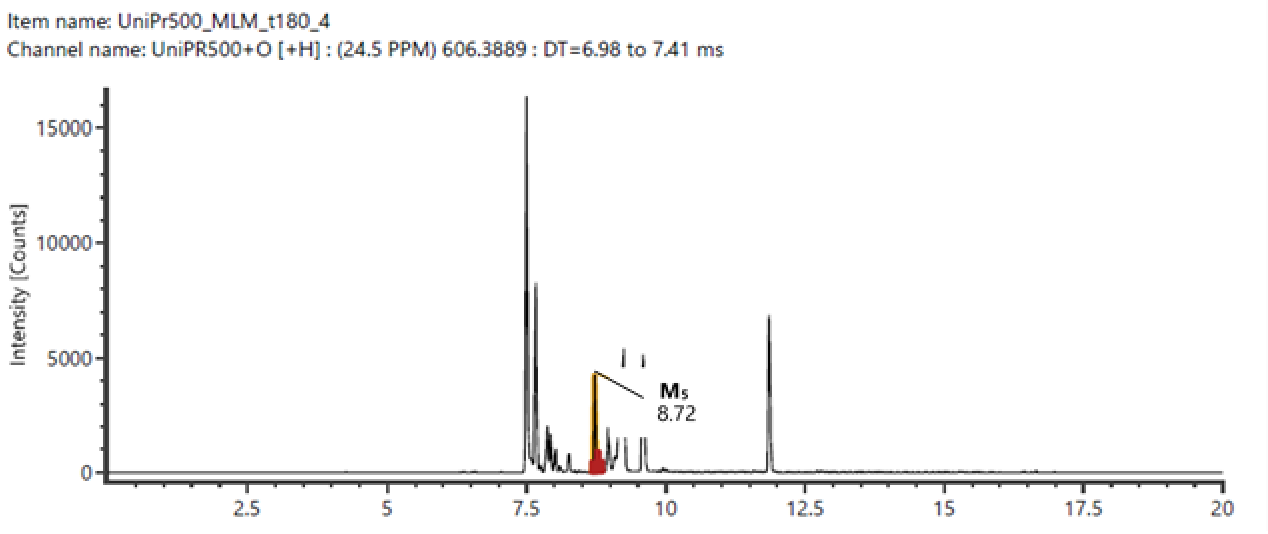

b. Experimental high resolution mass value in ESI$^+$ for **M5**.

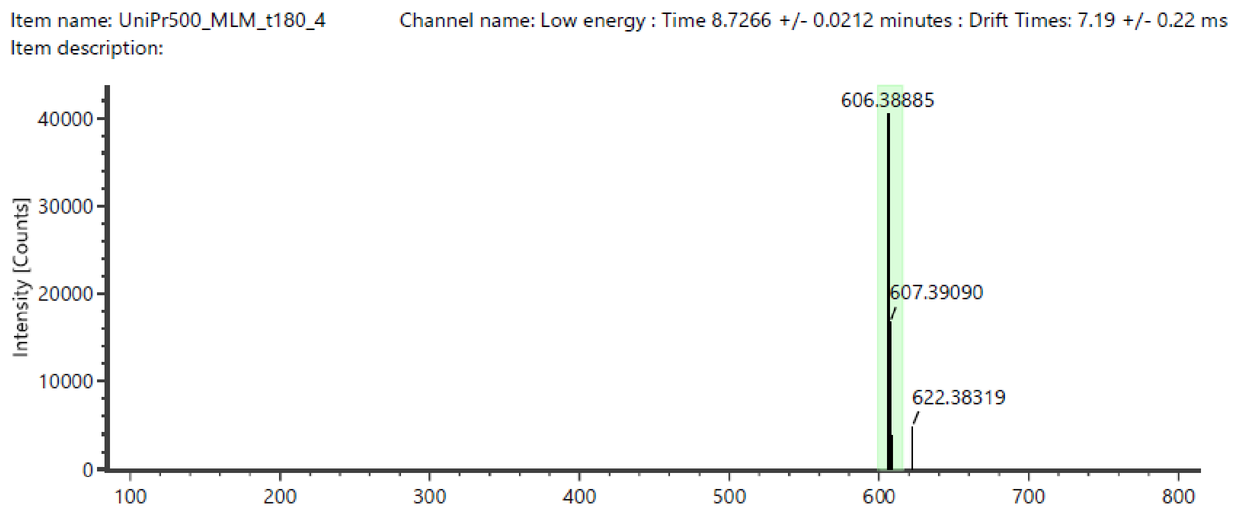

c. High resolution high energy (MS^E) MS spectrum in ESI$^+$ of **M5** together with tentative fragmentation pattern. While ion peaks at *m/z* = 588.4, 389.3, 372.3 and 172.07 have been previously described for M3, two peculiar fragment ions are present in the MS/MS spectrum at *m/z* = 218.1, which is compatible with the 4-(indol-3-yl)butanoic acid carrying and hydroxyl group; and at *m/z* = 200.1, which accounts for a loss of water of the previous one. Those two ion signals, together with the one at *m/z* = 172.07, suggest oxydation to occur on the L-β-homotryptophan.

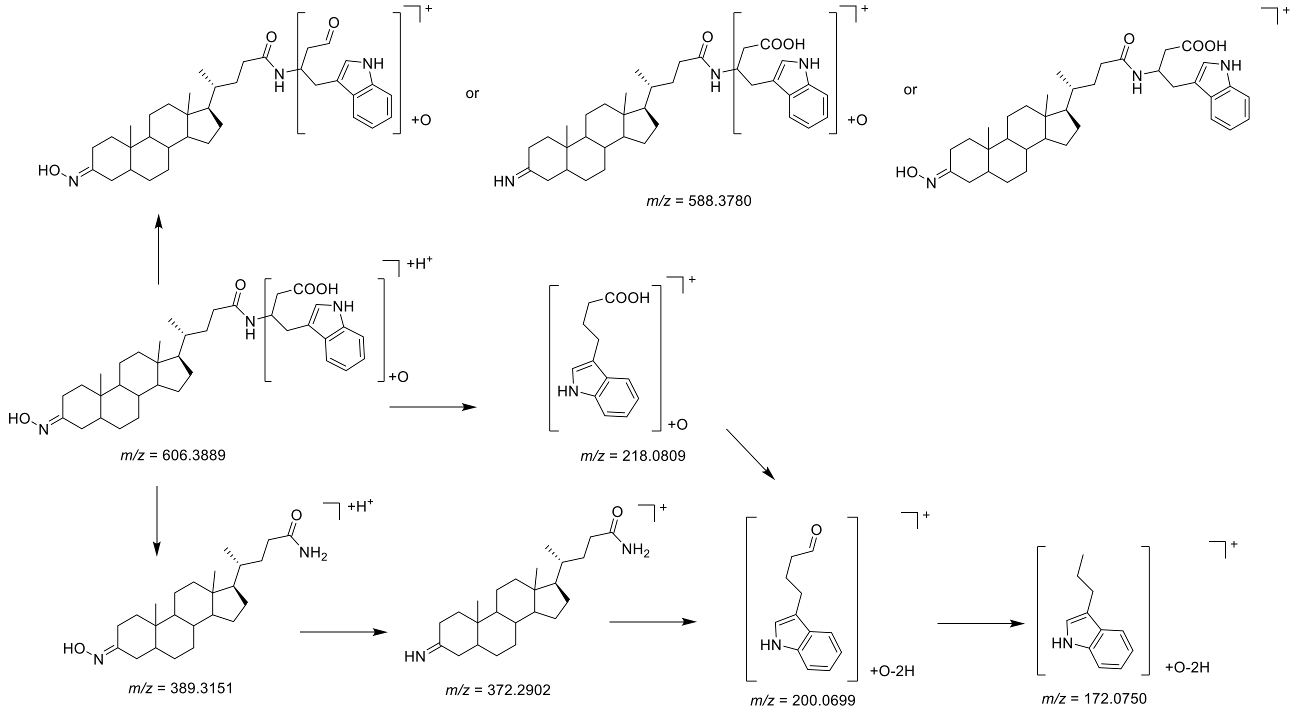

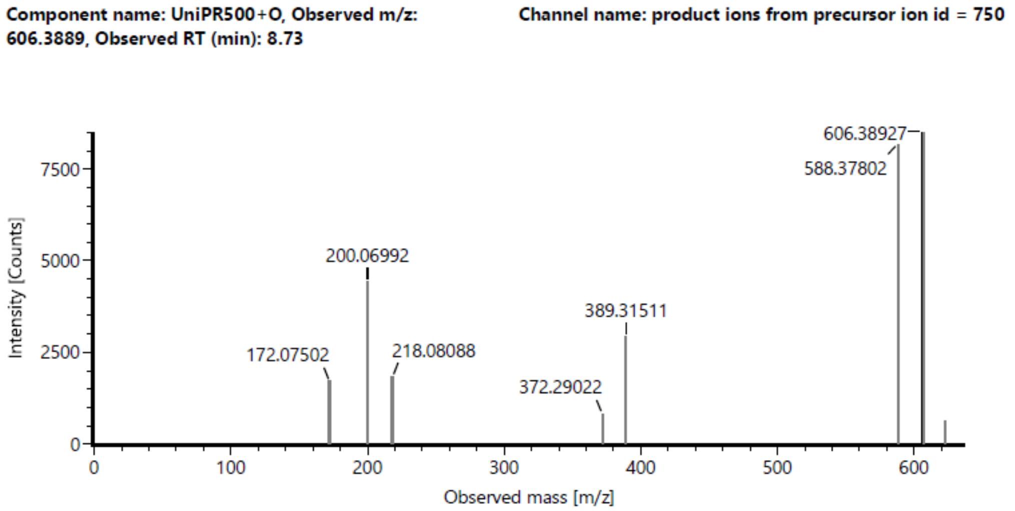

d. Metabolic soft spots on UniPR500 reported with a color-scale: the most probable sites of modification are highlighted in bright green. Based on the MS/MS spectrum of **M5**, UNIFI software returns as the most likely soft spots the C atoms of the propyl-indolic moiety of UniPR500.

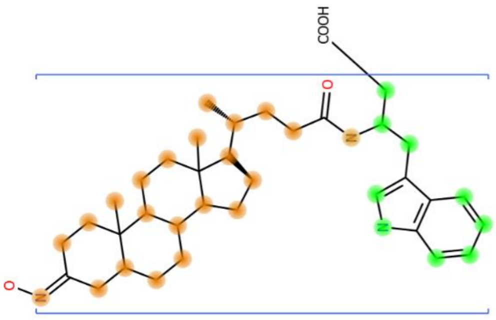

Figure S23. *a-d. Metabolite* ***M6*** *in MLM.*

a. Phase I Metabolite **M6** derived from UniPR500 in mouse liver microsomes. Extracted ion chromatogram (XIC) in ESI$^+$ at m/z = 606.39 [M+H]$^+$ and RT = 9.24 min.

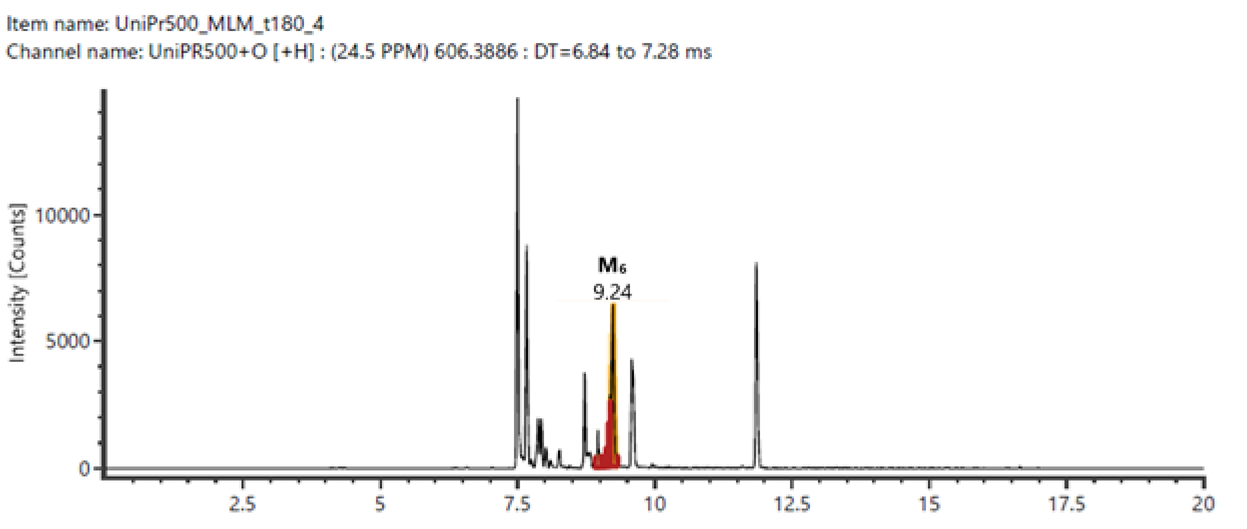

b. Experimental high resolution mass value in ESI⁺ for **M6**.

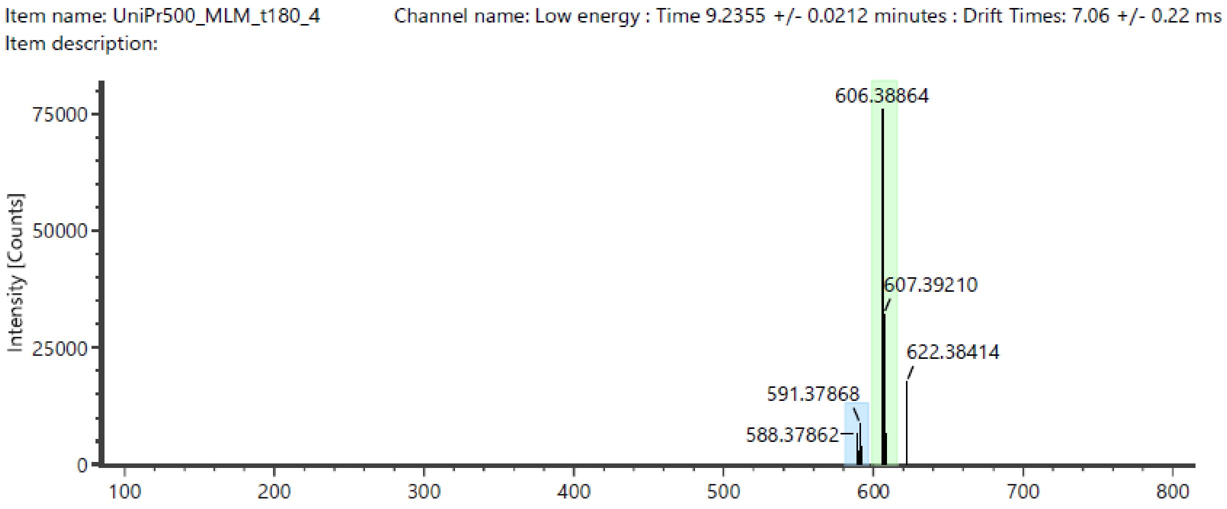

c. High resolution high energy (MS^E) MS spectrum in ESI⁺ of **M6** together with tentative fragmentation pattern. The base peak at *m/z* = 588.4 is compatible with dehydration (-18); fragment ion at *m/z* = 570.4 probably derived from the further loss of water and corresponds to the *m/z* = 575.4 reported for UniPR129 **M7**. Product ions at *m/z* = 389.3, 372.3 and 354.3 are shared with UniPR500, while peaks at *m/z* = 217.1 and 172.1- the latter accounting for oxidation on the 3-propyl-indole - are the same found in the MS/MS spectrum of UniPR129.

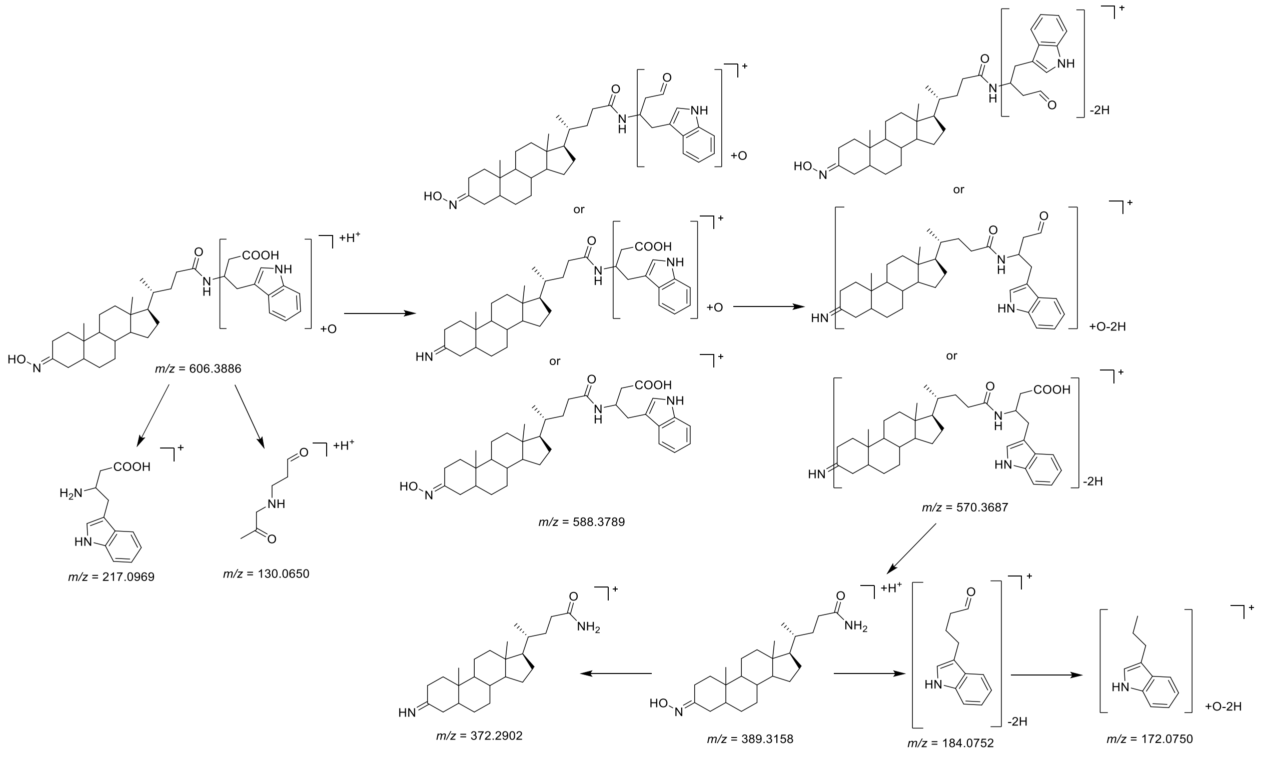

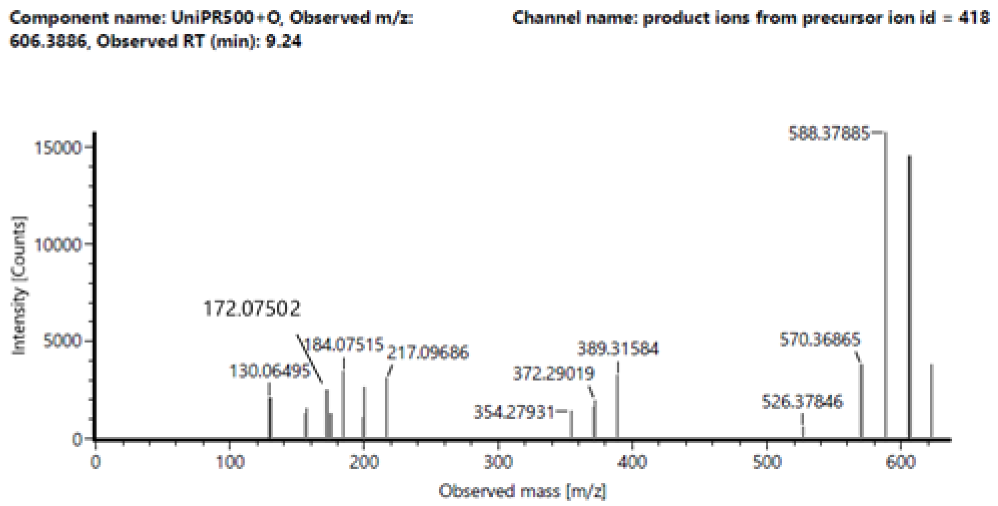

d. Metabolic soft spots on UniPR500 reported with a color-scale: the most probable sites of modification are highlighted in bright green. Based on the MS/MS spectrum of **M6**, UNIFI software returns as the most likely soft spots the propyl-indolic moiety of UniPR129.

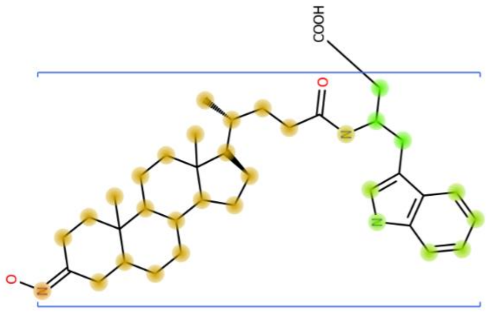

Figure S24. *a-d. Metabolite* ***M7*** *in MLM.*

a. Phase I Metabolite **M7** derived from UniPR500 in mouse liver microsomes. Extracted ion chromatogram (XIC) in ESI$^+$ at *m/z* = 606.39 [M+H]$^+$ and RT = 9.60 min.

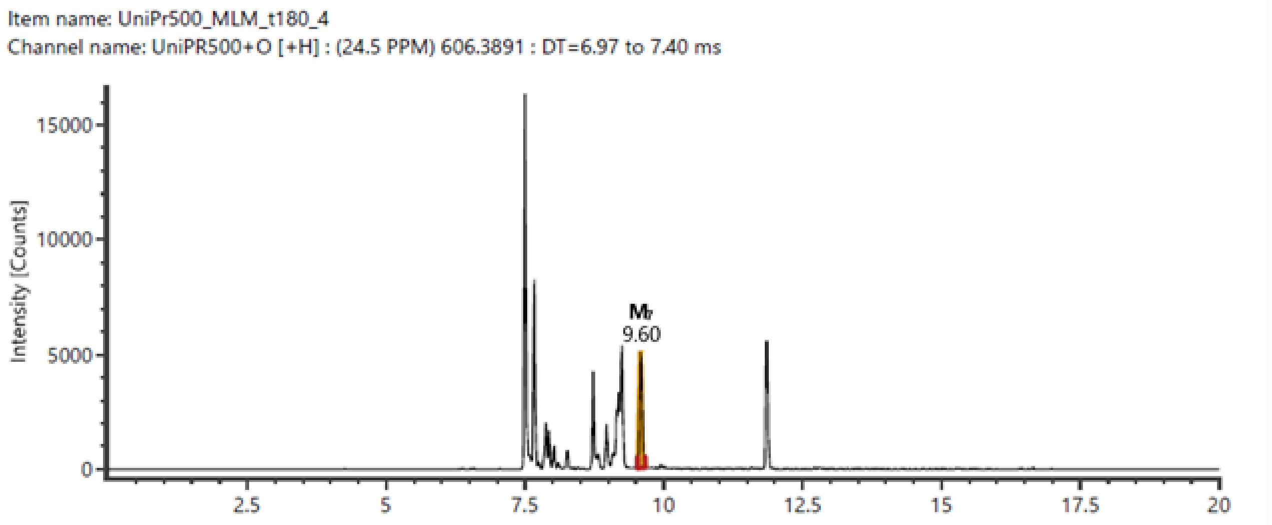

b. Experimental high resolution mass value in ESI$^+$ for **M7**.

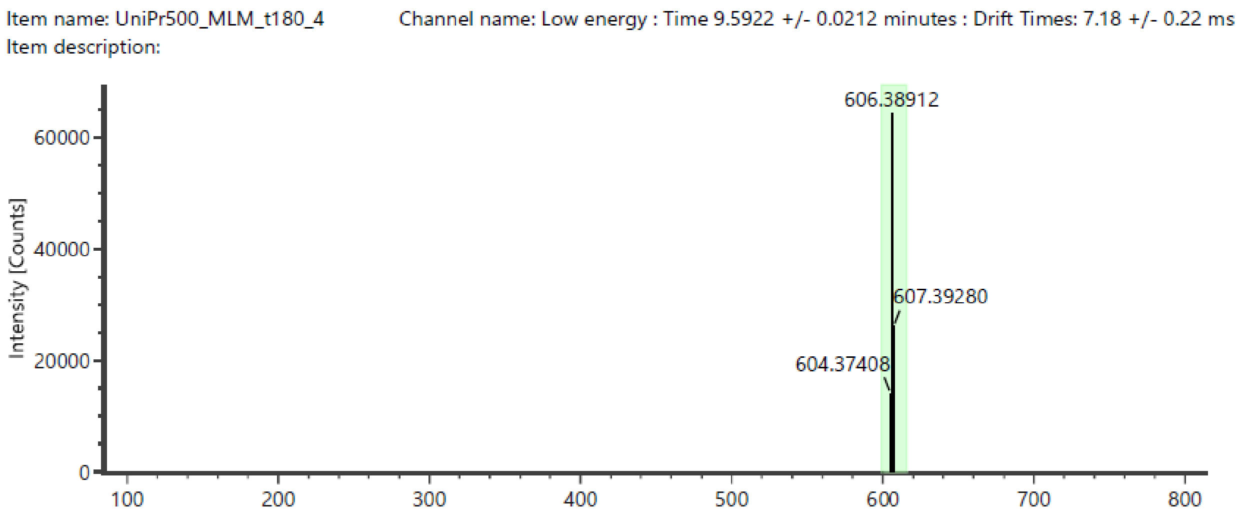

c. High resolution high energy (MS^E) MS spectrum in ESI$^+$ of **M7** together with tentative fragmentation pattern. Ion peaks at *m/z* = 588.4, 570.4, 389.3, 217.1 and 130.1 were also found in **M3** MS/MS spectrum. Peculiar fragment ion at *m/z* = 371.3 derived from *m/z* = 389.3 *via* dehydration; characteristic ion peak at *m/z* = 157.1 is compatible with the loss of a hydroxyl group (-17) from *m/z* = 172.1.

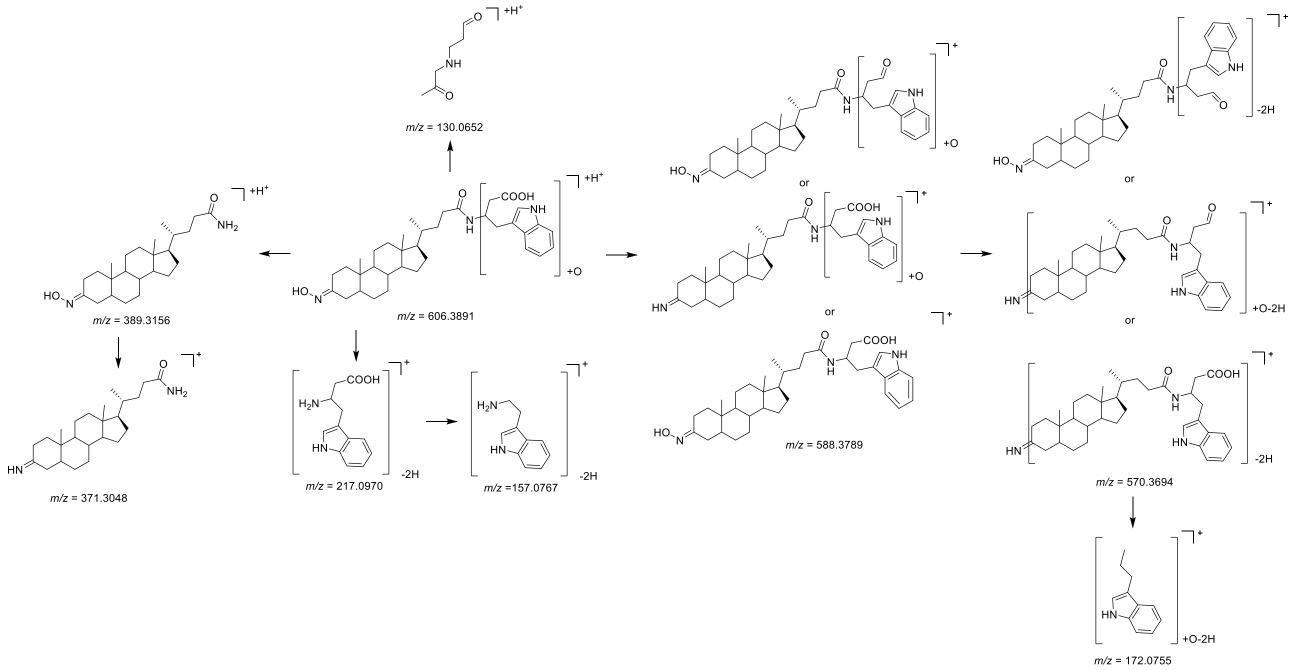

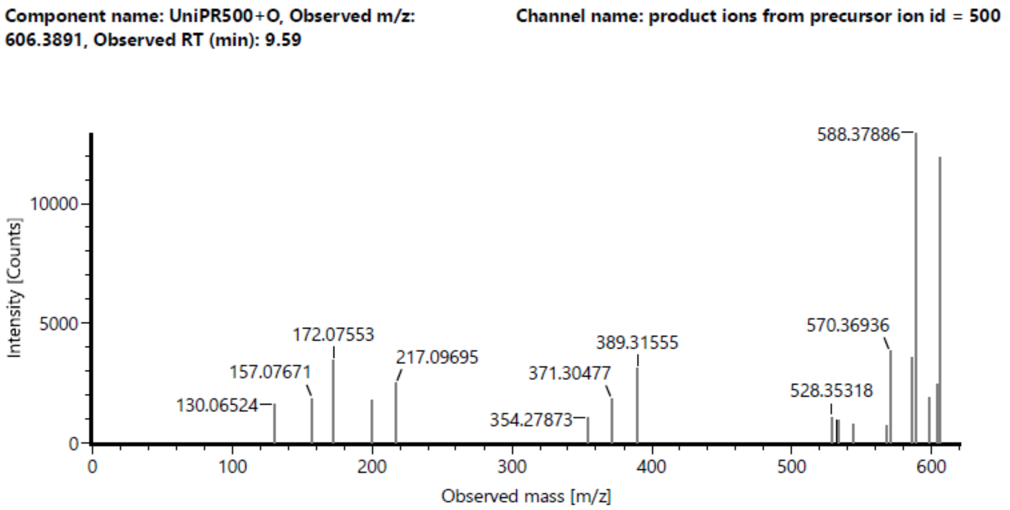

d. Metabolic soft spots on UniPR500 reported with a color-scale: the most probable sites of modification are highlighted in bright green. Based on the MS/MS spectrum of **M7**, UNIFI software returns as the most likely soft spots (i.e. the propyl-indole) found for **M5**.

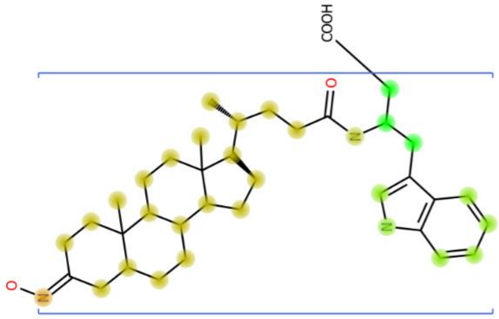

Figure S25. *a-d. Metabolite* ***M8*** *in MLM.*

a. Phase I Metabolite **M8** derived from UniPR500 in mouse liver microsomes. Extracted ion chromatogram (XIC) in ESI$^+$ at *m/z* = 606.39 [M+H]$^+$ and RT = 11.86 min.

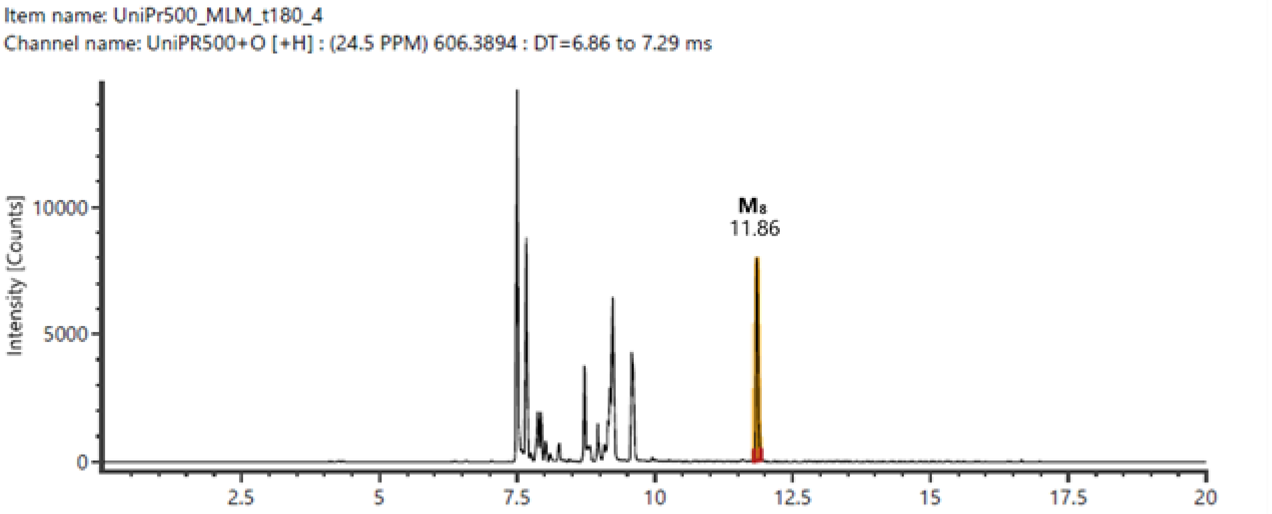

b. Experimental high resolution mass value in ESI+ for **M8**.

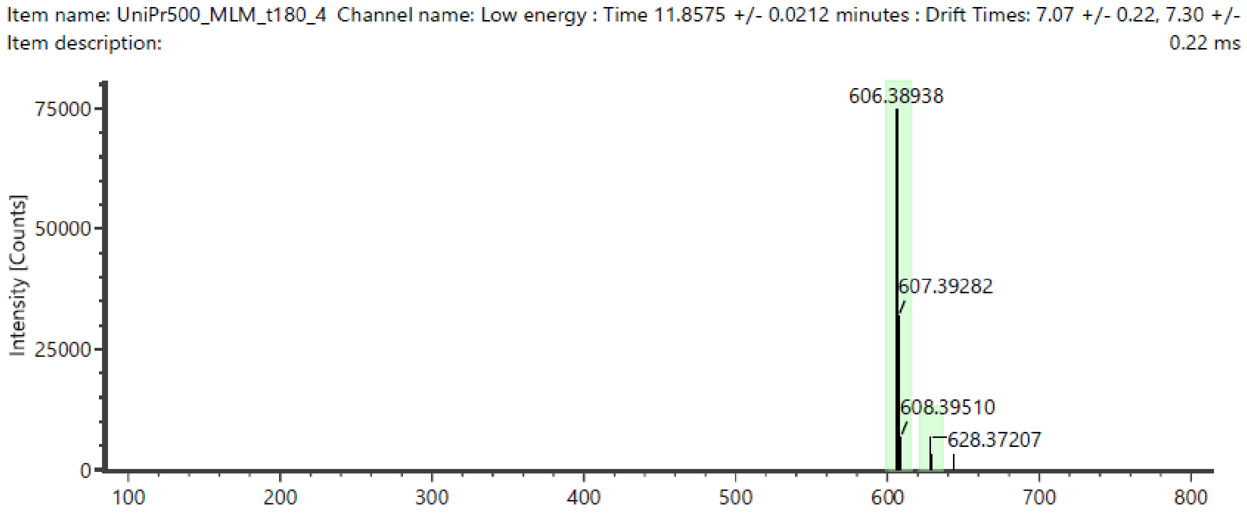

c. High resolution high energy (MS^E) MS spectrum in ESI+ of **M8** together with tentative fragmentation pattern. In the MS/MS spectrum, peak at *m/z* = 405.3 is the same found for **M4**. Characteristic fragment ion at *m/z* = 447.3 is compatible with the breaking of the steroidal core and the concomitant dehydroxylation of the carboxylic group.

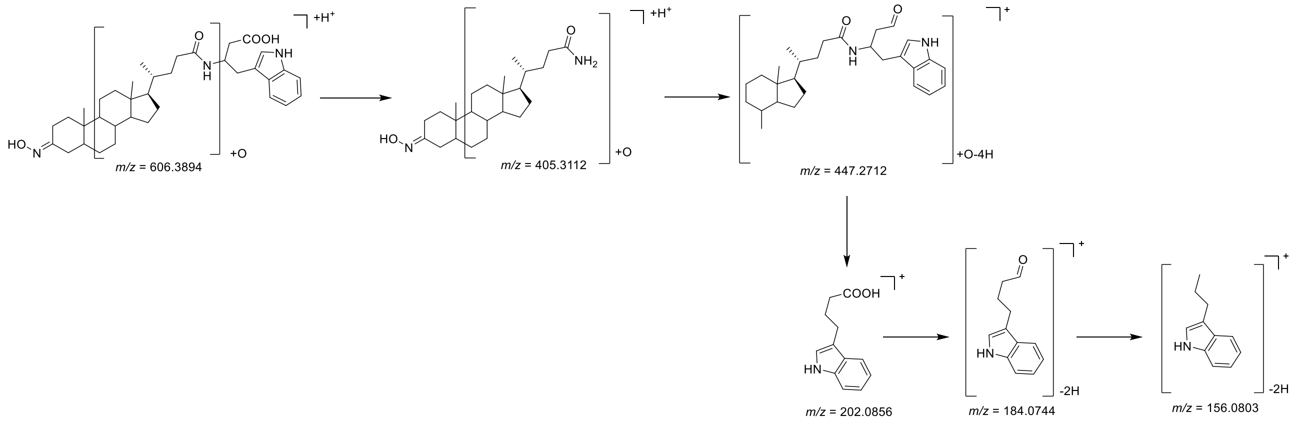

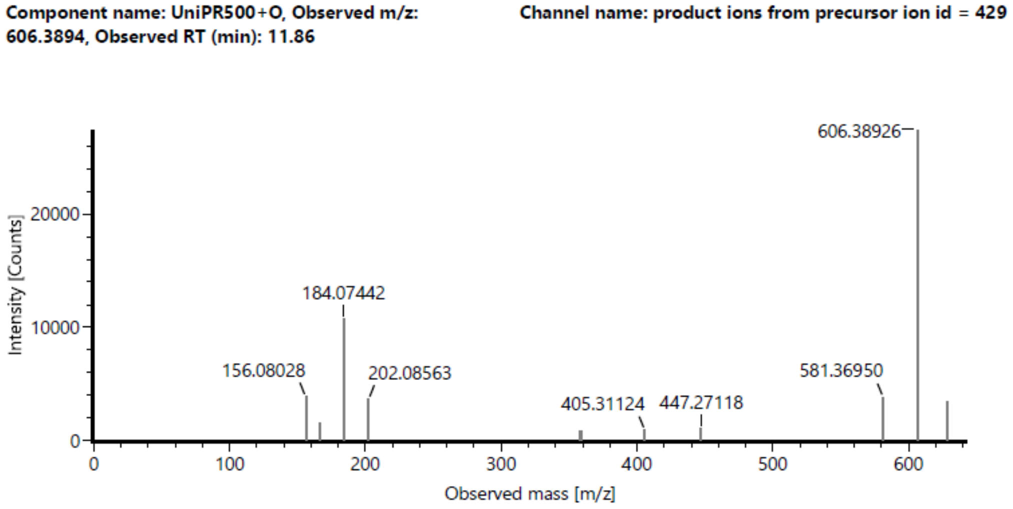

d. Metabolic soft spots on UniPR500 reported with a color-scale: the most probable sites of modification are highlighted in bright green. Based on the MS/MS spectrum of **M8**, UNIFI software returns as the most likely soft spots C-7-23 of the steroidal core.

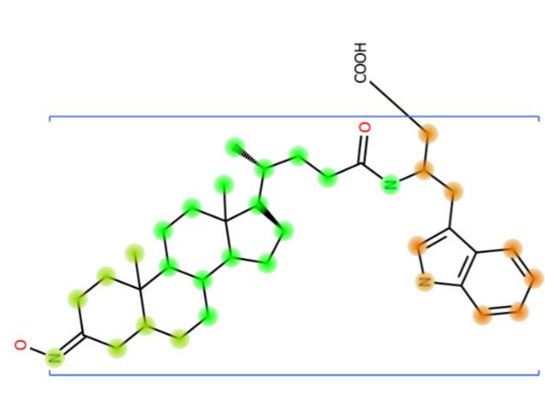

Figure S26. *a-c. Metabolite **M9** in MLM.*

a. Phase I Metabolite **M9** derived from UniPR500 in mouse liver microsomes. Extracted ion chromatogram (XIC) in ESI$^+$ at m/z = 622.38 [M+H]$^+$ and RT = 9.68 min.

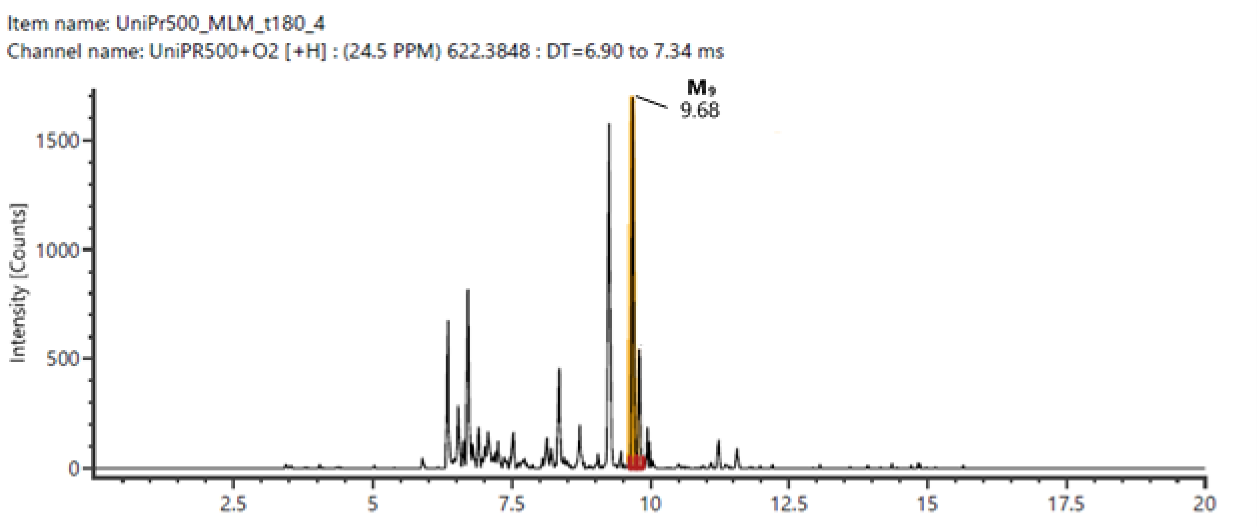

b. Experimental high resolution mass value in ESI$^+$ for **M9**.

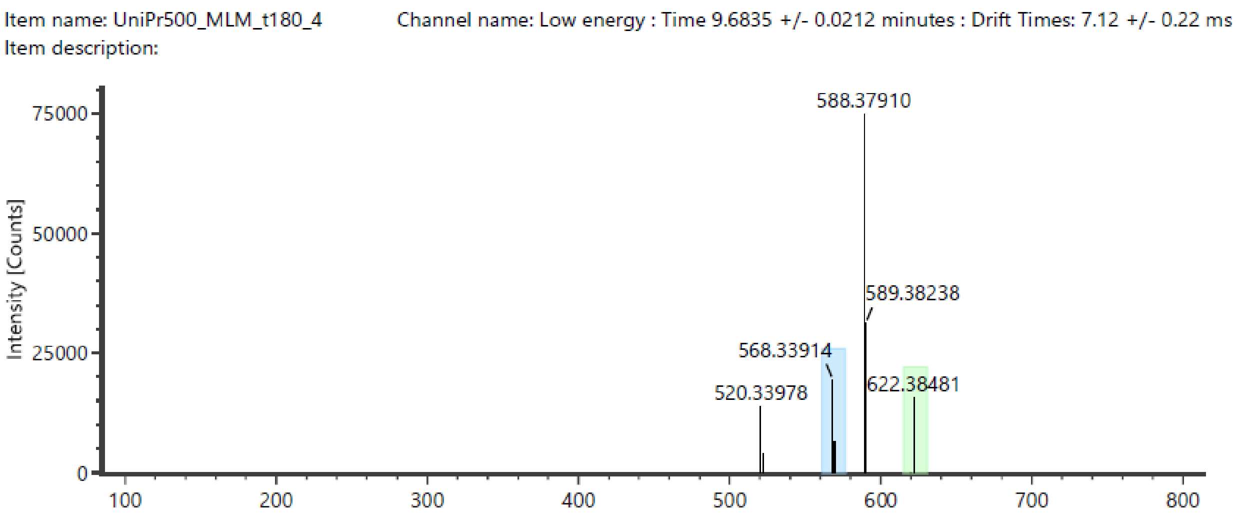

c. High resolution high energy (MS[E]) MS spectrum in ESI[+] of **M9** together with tentative fragmentation pattern. Fragment ions at *m/z* = 588.4 and 570.4 have been already described for **M7**, while *m/z* = 387.3 has been reported for **M2**. Peak at *m/z* = 568.3 could derive from a partial disruption of the steroid, which is further enhanced in the fragment ion at *m/z* = 482.3, which is compatible with a concomitant dehydroxylation of the carboxylic moiety. However, UNIFI software could not predict likely sites for oxidation.

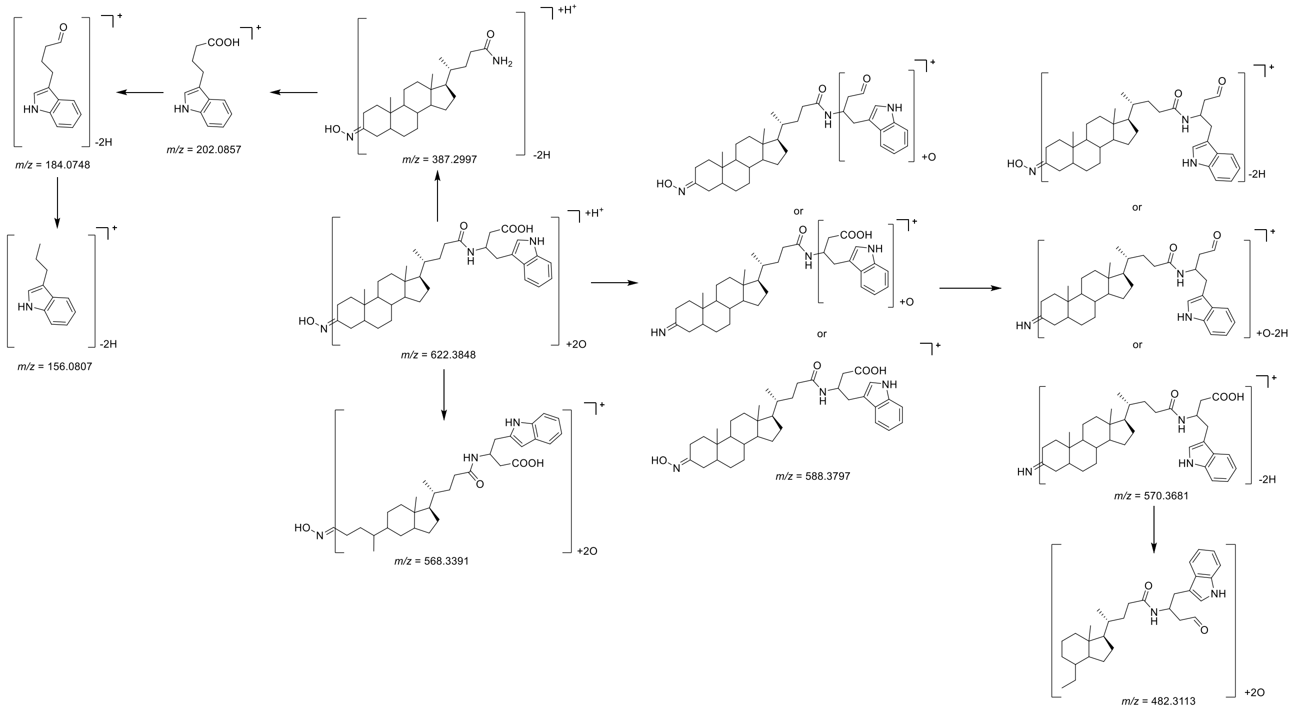

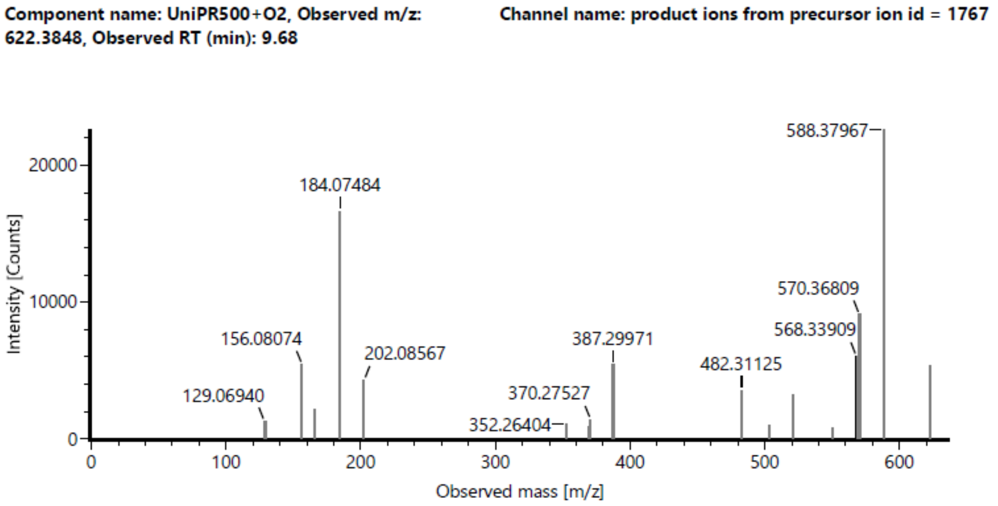

Figure S27. a-d. Metabolite ***M10*** *in MLM.*

a. Phase I Metabolite **M10** derived from UniPR500 in mouse liver microsomes. Extracted ion chromatogram (XIC) in ESI$^+$ at m/z = 591.38 [M+H]$^+$ and RT = 8.06 min.

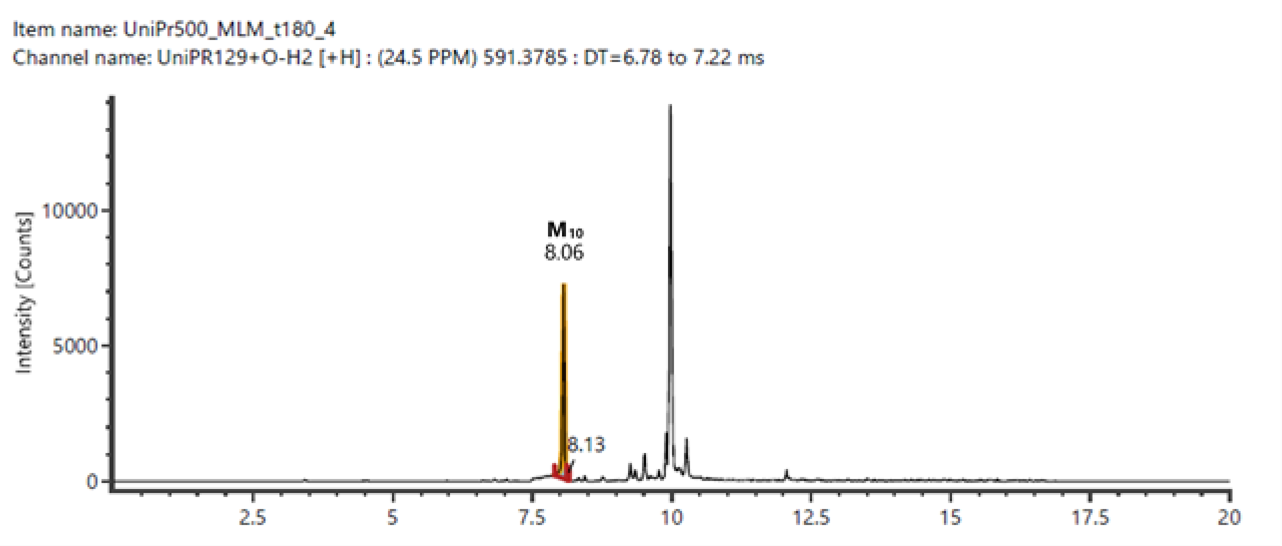

b. Experimental high resolution mass value in ESI$^+$ for **M10**.

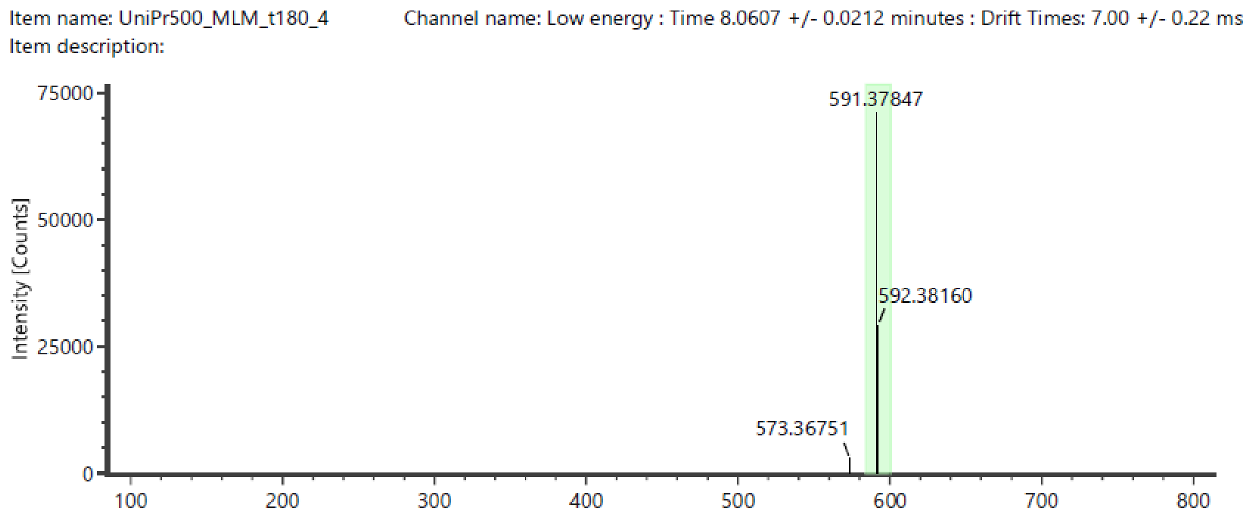

c. High resolution high energy (MS^E) MS spectrum in ESI$^+$ of **M10** together with tentative fragmentation pattern. The three product ions at *m/z* = 202.1, 184.1 and 156.1 prompt to exclude the oxidation to occur on L-β-homotryptophan group. Moreover, the low intense fragment ion at *m/z* = 337.2 allow to speculate that oxidation involves a C-atom on the A- or B-ring of the steroidal core.

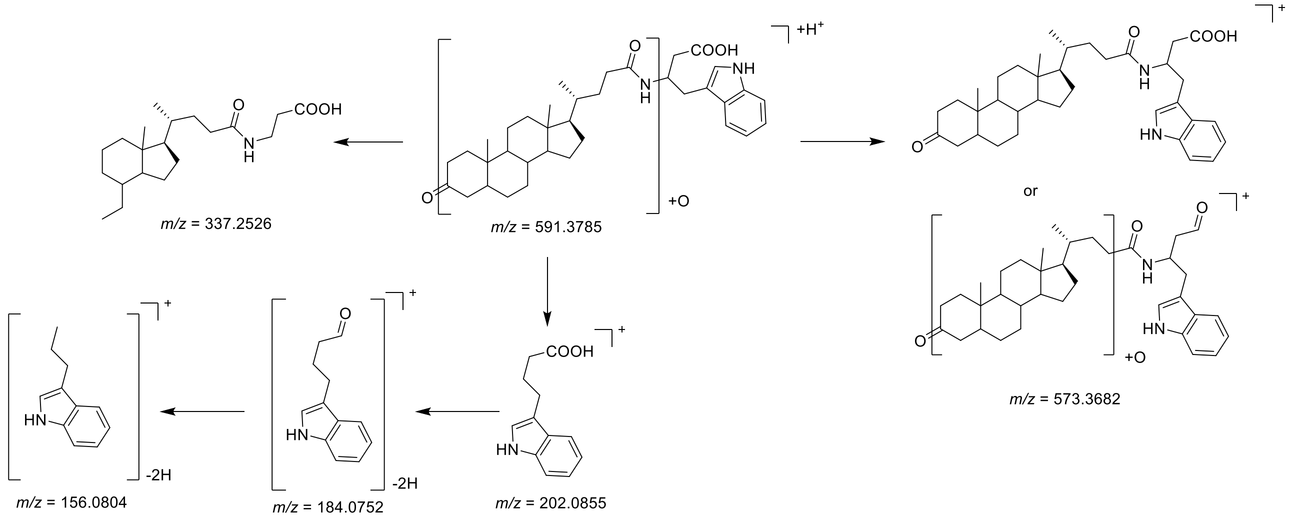

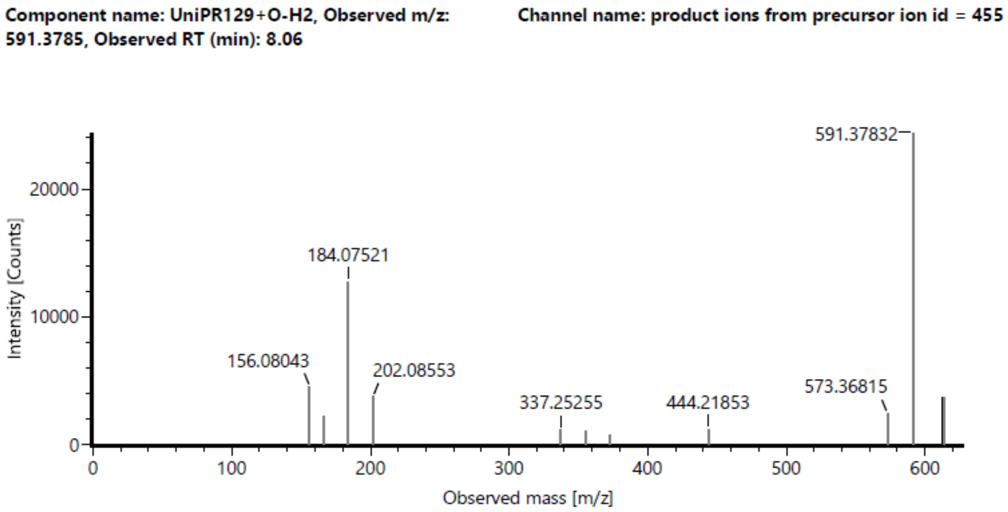

d. Metabolic soft spots on UniPR129 reported with a color-scale: the most probable sites of modification are highlighted in bright green. Based on the MS/MS spectrum of **M10**, UNIFI software returns as the most likely soft spots C1 or C2 of the steroidal core.

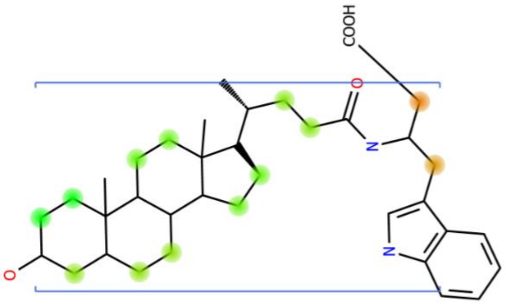

Figure S28. *a-d. Metabolite* ***M11*** *in MLM.*

a. Phase I Metabolite **M11** derived from UniPR500 in mouse liver microsomes. Extracted ion chromatogram (XIC) in ESI$^+$ at m/z = 593.39 [M+H]$^+$ and RT = 7.58 min.

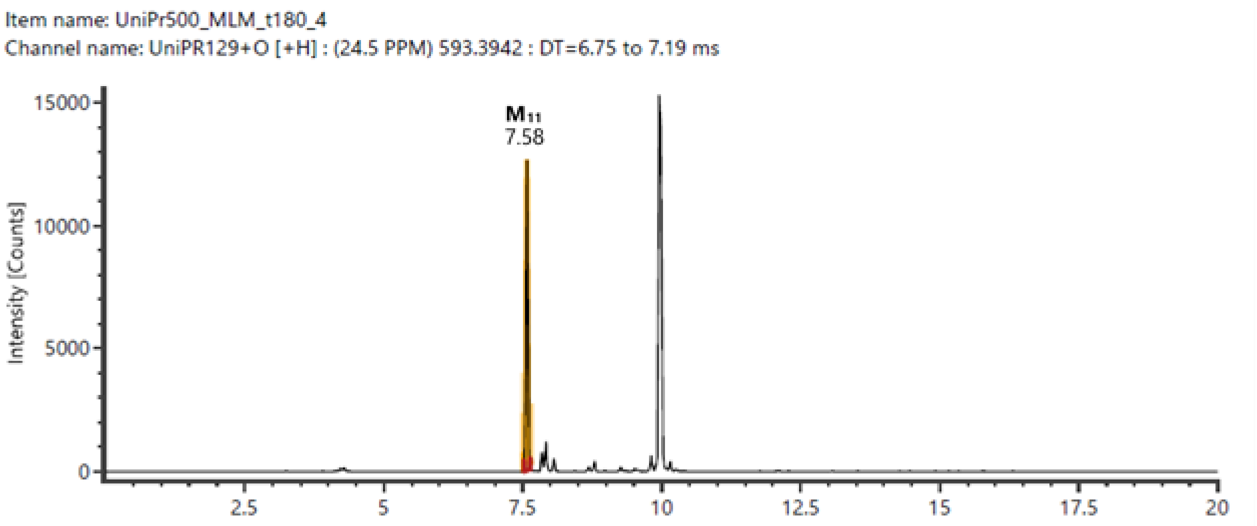

b. Experimental high resolution mass value in ESI+ for **M11**.

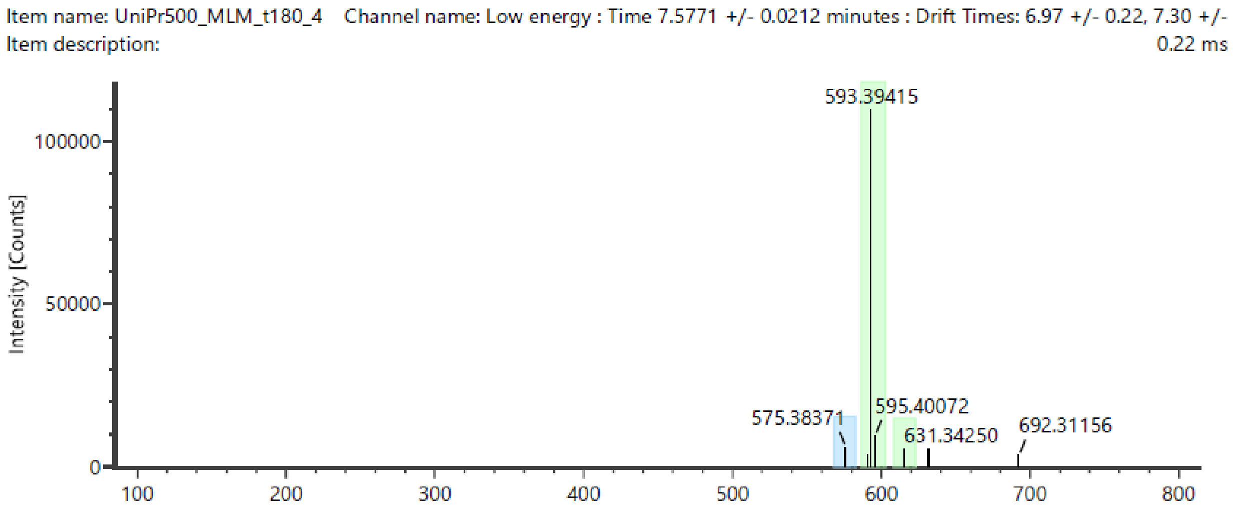

c. High resolution high energy (MS^E) MS spectrum in ESI+ of **M11** together with tentative fragmentation pattern. In the MS/MS spectrum three characteristic fragments can be found: the peak at *m/z* = 575.4, compatible with the loss of water; product ion at *m/z* = 374.3 derived from parent 593.4 by dehydration and loss of the 4-(indol-3-yl)butanoic acid fragment; product ion at *m/z* = 219.1 consisting of the L-β-homotryptophan group. The other fragment ions recall the ones from **M4** fragmentation.

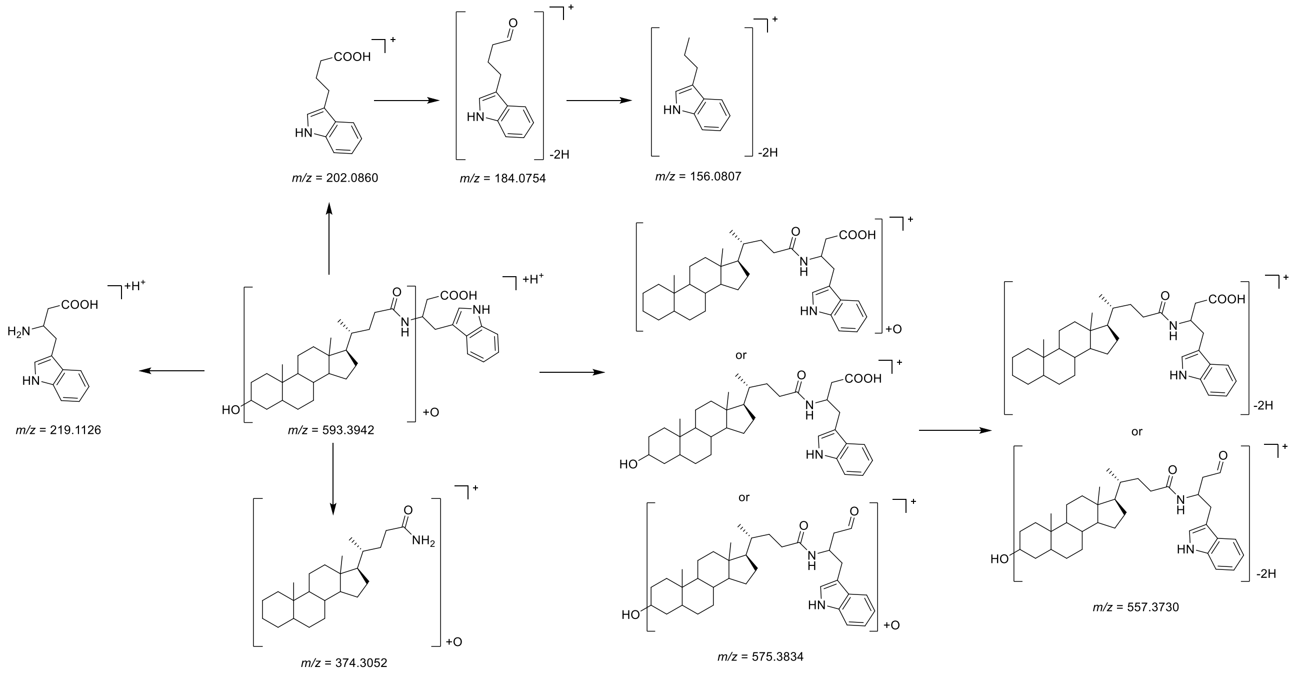

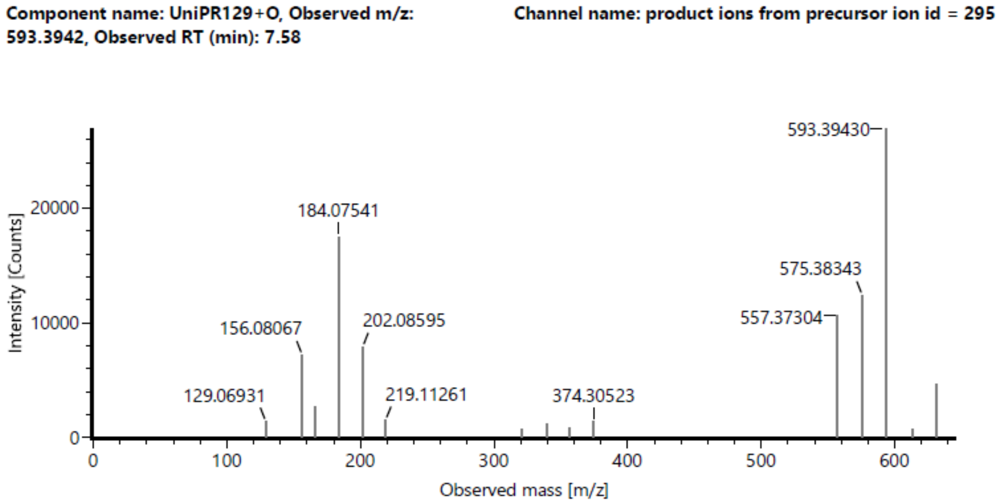

d. Metabolic soft spots on UniPR500 reported with a color-scale: the most probable sites of modification are highlighted in bright green. Based on the MS/MS spectrum of **M11**, UNIFI software returns as the most likely soft spots C-7-23 of the steroidal core.

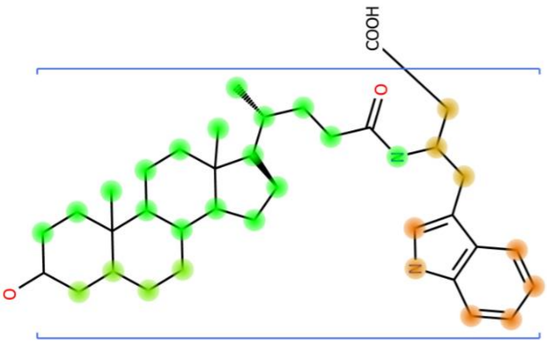

S.9 UniPR500 *In vitro* phase II metabolite profiling

Figure S29. *a-d. Metabolites* ***M12-M14*** *in MLS9 fraction.*

a. Phase II conjugates of UniPR500 with glucuronic acid **M12-M14** in mouse liver microsomes. Upper trace: Extracted ion chromatogram (XIC) in ESI$^-$ at *m/z* = 588.38 [M-H]$^-$ corresponding to parent UniPR500; lower trace: XIC in ESI$^-$ at *m/z* = 764.41 [M-H]$^-$ corresponding to the glucuronides.

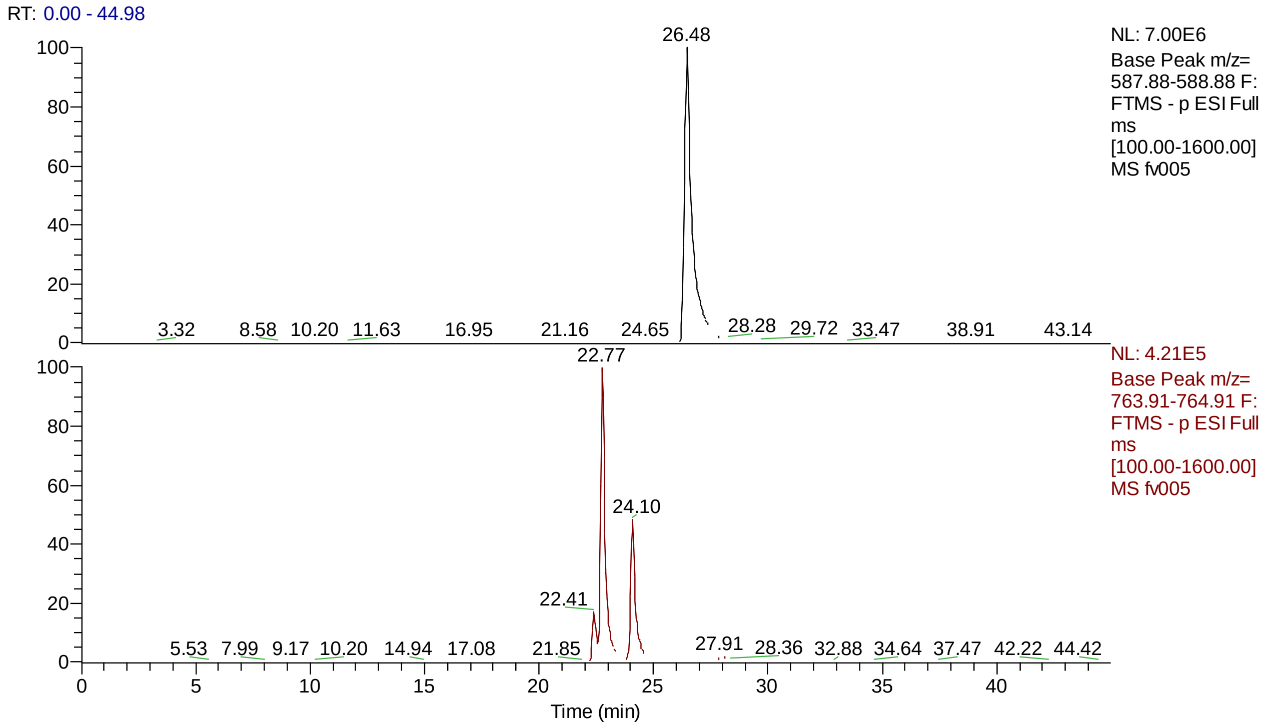

b. Experimental high resolution mass values and isotopic distribution in ESI$^-$ for metabolite **M12** (upper, RT = 22.77 min) if compared to calculated value (lower spectrum).

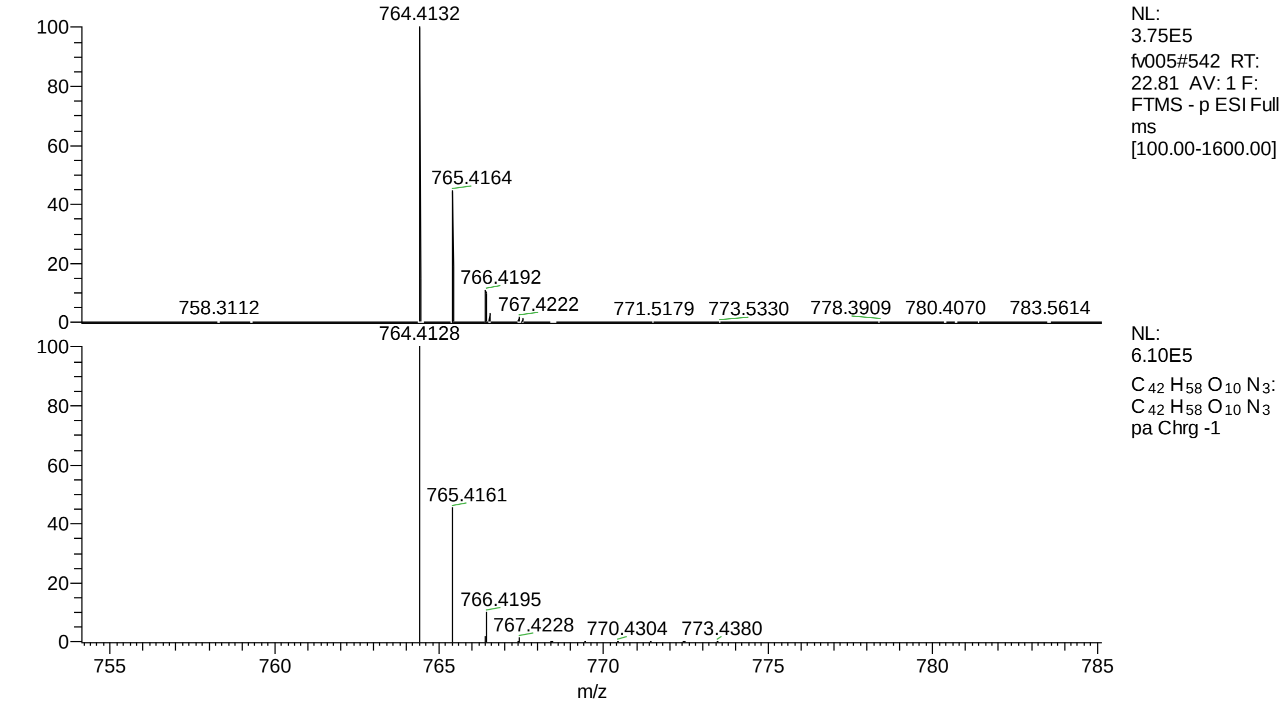

c. Experimental high resolution mass values and isotopic distribution in ESI⁻ for metabolite **M13** (upper, RT = 22.41 min) if compared to calculated value (lower spectrum).

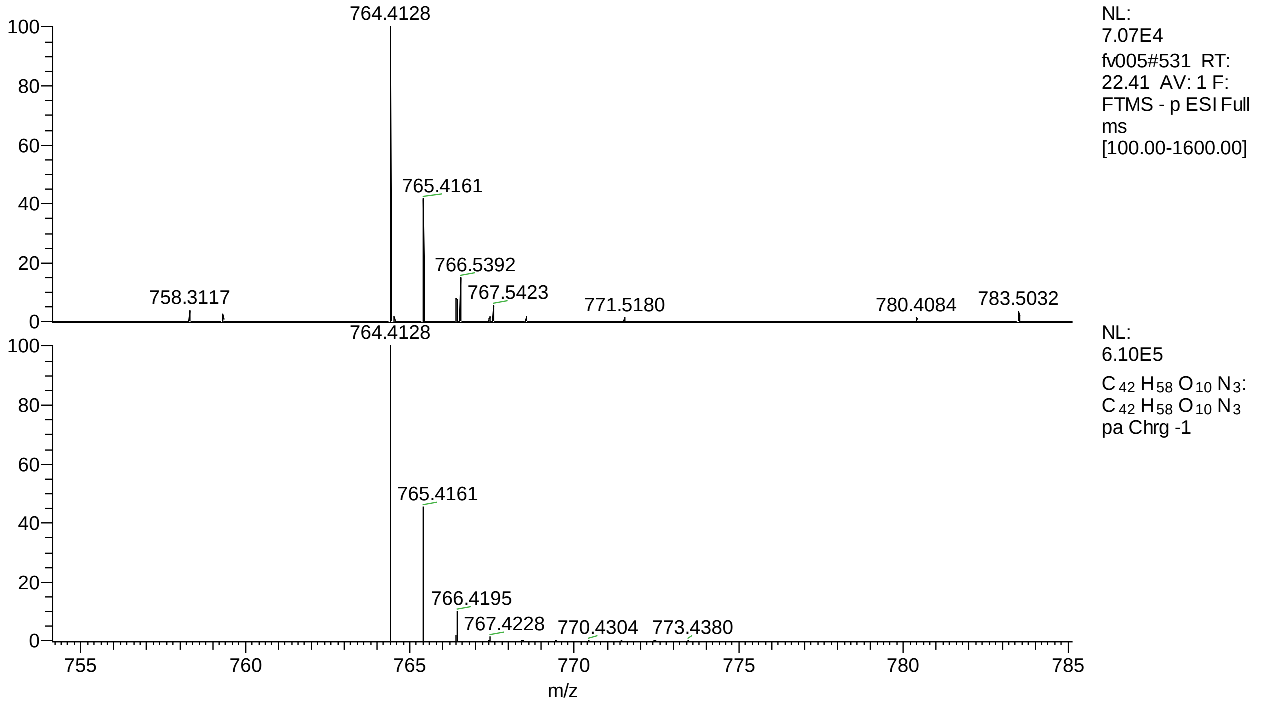

d. Experimental high resolution mass values and isotopic distribution in ESI⁻ for metabolite **M14** (upper, RT = 24.10 min) if compared to calculated value (lower spectrum).

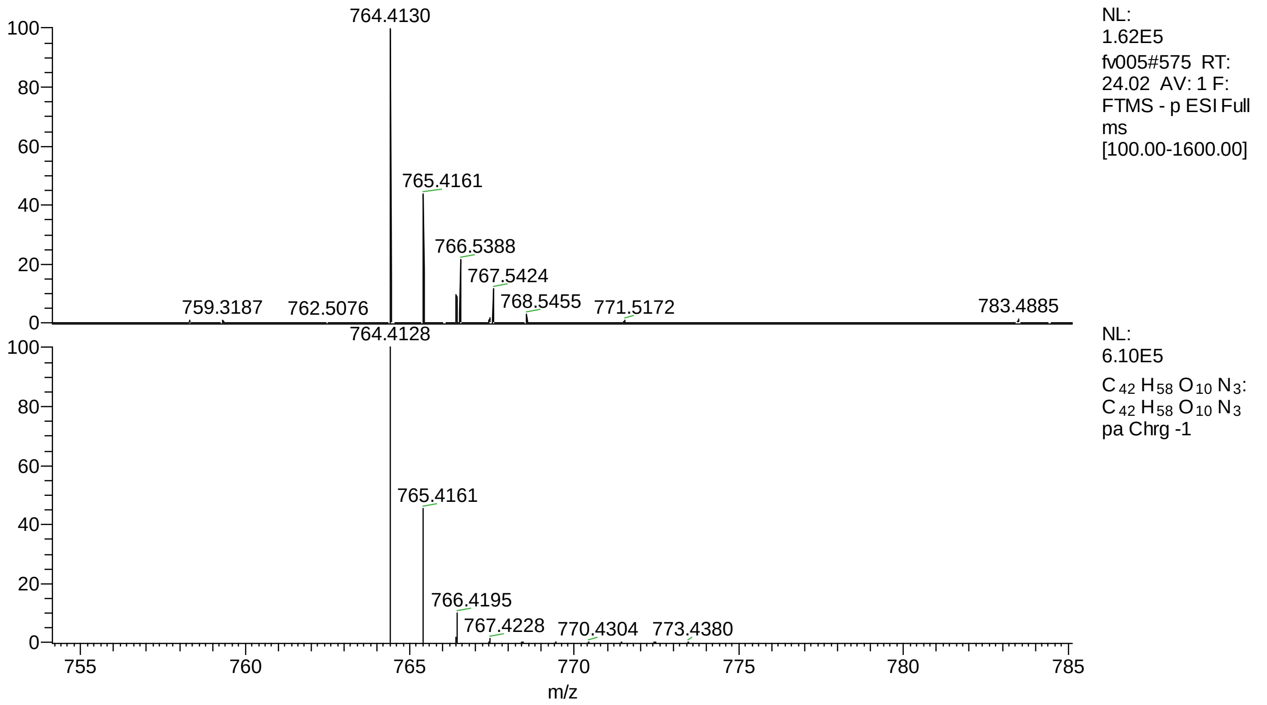

Figure S30. a-b. Metabolite ***M15*** *in HLS9 fraction.*

a. Phase II conjugate of UniPR500 with active sulphate **M15** in human liver S9 fraction. Upper trace: Extracted ion chromatogram (XIC) in ESI$^-$ at *m/z* = 588.38 [M-H]$^-$ corresponding to parent UniPR500; lower trace: XIC in ESI$^-$ at *m/z* = 668.34 [M-H]$^-$ corresponding to the conjugate with active sulphate.

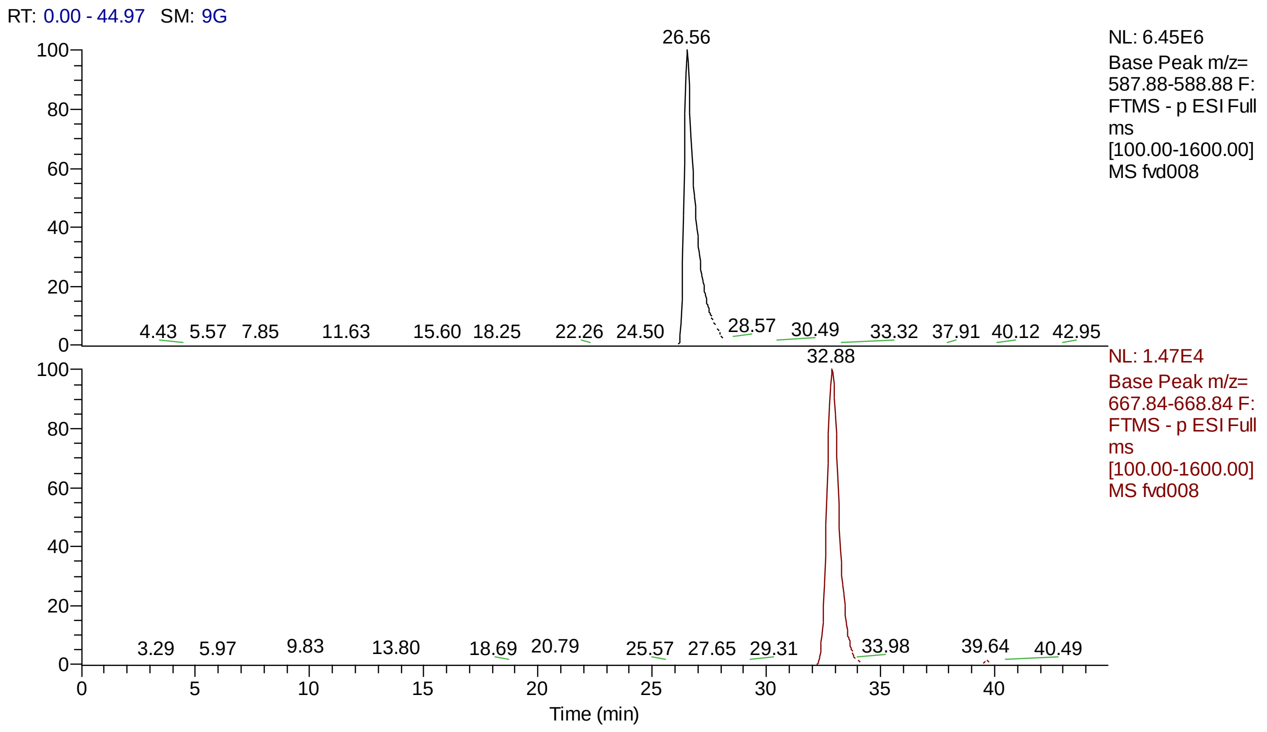

b. Experimental high resolution mass values and isotopic distribution in ESI$^-$ for metabolite **M15** (upper, RT = 32.9 min) if compared to calculated value (lower spectrum).

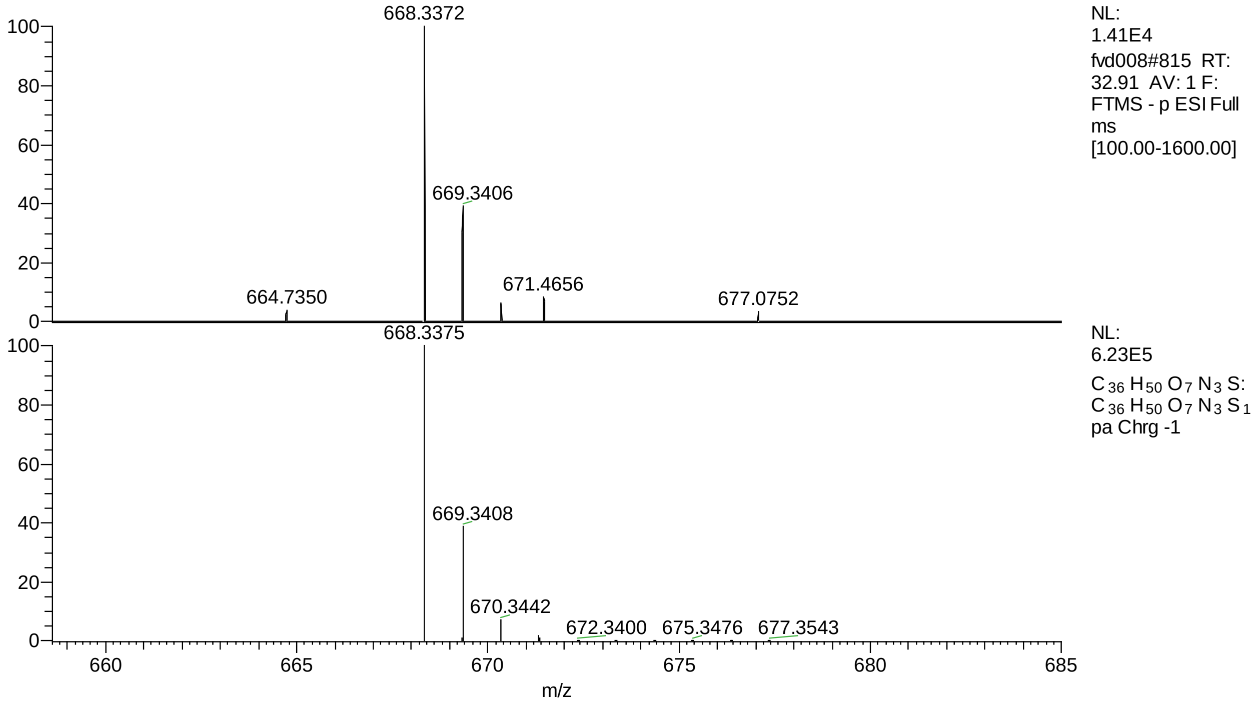

Supplement: Supplementary file 1 [file pharmaceuticals-15-00041-s001.zip › pharmaceuticals-1508891-supplementary.pdf]
